# A new seal from the Late Miocene of the Eastern Paratethys highlights the past regional diversity of true seals (Phocidae)

**DOI:** 10.1186/s13358-025-00372-7

**Published:** 2025-06-12

**Authors:** Pavlo Otriazhyi, Theodor Obadă, Oleksandr Kovalchuk, Davit Vasilyan, Pavel Gol’din

**Affiliations:** 1 Department of Evolutionary Morphology, Schmalhausen Institute of Zoology, National Academy of Sciences of Ukraine, Kyiv, Ukraine; 2 Jurassica Museum, Porrentruy, Switzerland; 3 Department of Geosciences, University of Fribourg, Fribourg, Switzerland; 4 Institute of Zoology, Academy of Sciences of Moldova, Chișinău, Moldova; 5 National Museum of Ethnography and Natural History, Chișinău, Moldova; 6 Department of Palaeontology, National Museum of Natural History, National Academy of Sciences of Ukraine, Kyiv, Ukraine; 7 Department of Palaeozoology, Faculty of Biological Sciences, University of Wrocław, Wrocław, Poland; 8 Department of Biology and Biology Teaching Methodology, Faculty of Natural Sceince and Geography, A.S. Makarenko Sumy State Pedagogical University, Sumy, Ukraine

**Keywords:** Seals, Marine mammals, Miocene, Evolution, Taxonomy, Phylogeny, Palaeoecology

## Abstract

True seals rapidly evolved in many forms in the epicontinental basin of Paratethys during the Miocene. However, most of their nominal taxa so far were proposed based on isolated limb bones, and their taxonomy has long been under discussion*.* Here we describe a new articulated skeleton MCFFM V-150 of a medium-sized seal with pachyosteosclerotic postcranial bones from the Late Miocene of the present-day Moldova and propose a new genus and species for it—*Paratethyphoca libera*. It is distinguished in the presence of a supraorbital process of the frontal bone in its posterior portion, a long snout, a proportionally long humerus (88% of the skull length), a short deltoid crest of the humerus, and a low supraspinatus fossa of the scapula. We also suggest this taxonomic identification for other Paratethyan seals. Phylogenetic analysis placed *Paratethyphoca libera* among other stem Phocinae described so far from the Paratethys; however, its close relationship to a living hooded seal *Cystophora cristata* cannot be ruled out*.* Additionally, MCFFM V-150 showed tooth wear interpreted as a sign of suction prey capture strategy, shared by another Paratethyan seal *Monachopsis pontica* and the living bearded seal *Erignathus barbatus*.

**Supplementary Information:**

The online version contains supplementary material available at 10.1186/s13358-025-00372-7.

## Introduction

The family of true seals (Phocidae) evolved by the latest Oligocene, and their earliest undoubtful fossil remains (*Noriphoca gaudini*) are dated back to around 23–22 Ma, coming from the Mediterranean region (Dewaele et al., 2018; also discussed in: Park et al., 2024). By the Miocene at the latest, seals colonized the Paratethys, a large epicontinental sea in Central Eurasia, recorded from the Upper Badenian deposits (13.82–12.65 Ma) of present-day Slovakia (Koretsky & Holec, 2002; Koretsky & Rahmat, 2015). By the end of the Middle Miocene, the Paratethys has been increasingly isolated from the global ocean and fell apart into the Central and Eastern Paratethys (Kováč et al., 2007; Lazarev et al., 2025; Popov et al., 1993; Vernyhorova et al., 2023). For that interval known in the previous literature as the Sarmatian sensu lato, three regional stratigraphic substages (Volhynian (early) (12.65–12.05 Ma), Bessarabian (middle) (12.05–9.90 Ma), and Khersonian (late Sarmatian *s.l.*) (9.90–7.65 Ma)) are recognized for the Eastern Paratethys (Gradstein et al., 2020; the timeline follows Lazarev et al., 2025), and all of these units have been recently suggested as stratigraphic stages (Lazarev et al., 2025). The isolation of the basins within the Paratethyan domain led to speciation and endemism of local marine mammal fauna during the Volhynian and Bessarabian (Gol’din et al., 2020; Grigorescu, 1976; Koretsky, 2001; Lazarev et al., 2025 and references therein). True seals were among the groups which diversified in the Eastern Paratethys. Some of them had unusual traits like a dwarf body size (*Monachopsis pontica*), a very wide distal end of the femur (*Pontophoca sarmatica*), or extremely pachyosclerotic bones (aforementioned taxa and *Pachyphoca* spp.). Most of the formally described Paratethyan taxa have been based on a limited disarticulated material, so now their revision is pending, especially in the context of recent studies and reviews (e.g., Dewaele et al., 2018; Rule et al., 2024). Otriazhyi et al. (2025) showed that some Paratethyan seals (e.g., *Monachopsis pontica*) have stem or crown positions within the phocine phylogeny, suggesting their significant role in true seal evolution.

The study of Paratethyan seals had a long history and started in the nineteenth century with the descriptions of true seals (Phocidae) from Kerch, Crimea, Ukraine (“*Phoca*”* pontica*; Eichwald, 1850) and Chișinău, Moldova (“*Phoca*”* maeotica*; Nordmann, 1860). The former taxon is currently referred to as *Monachopsis pontica*, recently redescribed, and it was among the smallest seals ever reported (Koretsky, 2001; Otriazhyi et al., 2025). The latter one was redescribed as *Cryptophoca maeotica* (Koretsky, 2001; Koretsky & Ray, 1994), whose humeri were slightly larger (mean length—106 mm; Koretsky, 2001) than those in the smallest modern seals (*Pusa caspica* and *Pusa sibirica*, ca. 70–90 mm; personal observation by PO).

Simionescu (1925) described a medium-sized seal as *Phoca bessarabica* on several postcranial fragments from the Bessarabian limestone deposits in Chișinău (Moldova)*.* These fossils were considered as belonging to the largest of Eastern Paratethyan seals for a while, until Koretsky and Rahmat (2013) described seals of the same size, distinct in pachyosclerotic skeletal features, from the Bessarabian deposits of Ukraine. The new genus, *Pachyphoca*, included two species, a smaller (*Pachyphoca ukrainica*) whose holotype is an isolated humerus, and a larger one (*Pp. chapskii*), the holotype of which is an isolated incomplete femur. *Pachyphoca chapskii* had a size comparable to that in *Ph. bessarabica*. Rule et al. (2024) suggested all these taxa as *nomina dubia* because of their fragmentary nature, supposing isolated limb elements as non-diagnostic by definition.

Here we describe a new articulated specimen MCFFM V-150 of an equally large seal which was found in the Bessarabian limestones near Mîrzeşti (Republic of Moldova) in 2014. It includes a skull and postcranial (interestingly, pachyosclerotic) elements, including a scapula, both humeri, a partial femur and some other limb bones, and therefore combines an undoubtedly diagnostic skull and limb elements which can be compared with previous findings. We compare this newly found material with putatively related Paratethyan seals, review their morphology in detail and, as a result, introduce a new taxon *Paratethyphoca libera* based on the specimen MCFFM V-150. In addition, we assess the phylogenetic context of the newly described taxon and other Paratethyan seals and analyze its palaeoecology based on anatomical traits and tooth wear.

## Methods

**Fig. 1:**
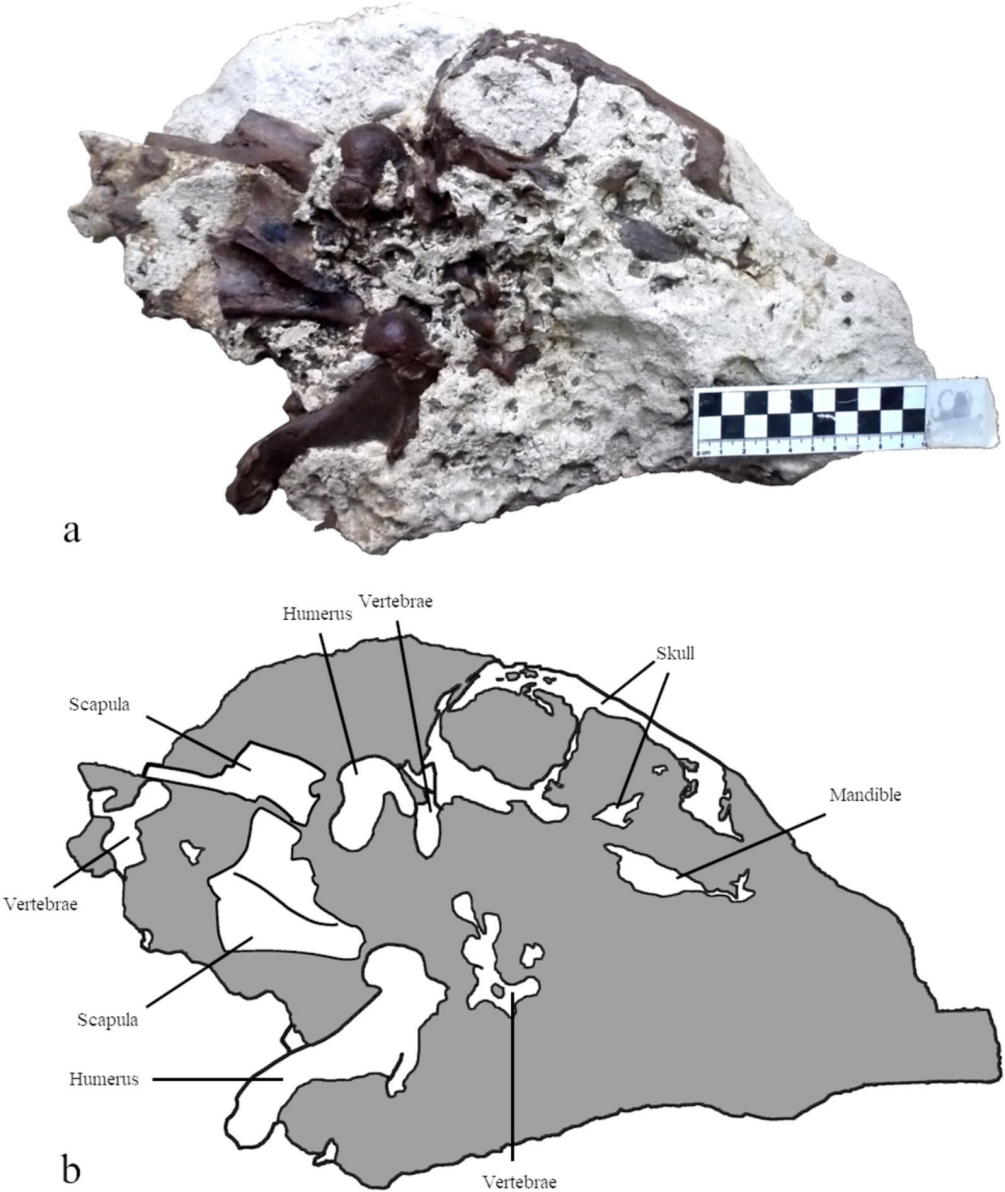
Skull and forelimbs of MCFFM V-150 *Paratethyphoca libera* in the matrix limestone (**a**) and their outline (**b**). Bones are shown in white on the outline, and the matrix is in grey

**Fig. 2:**
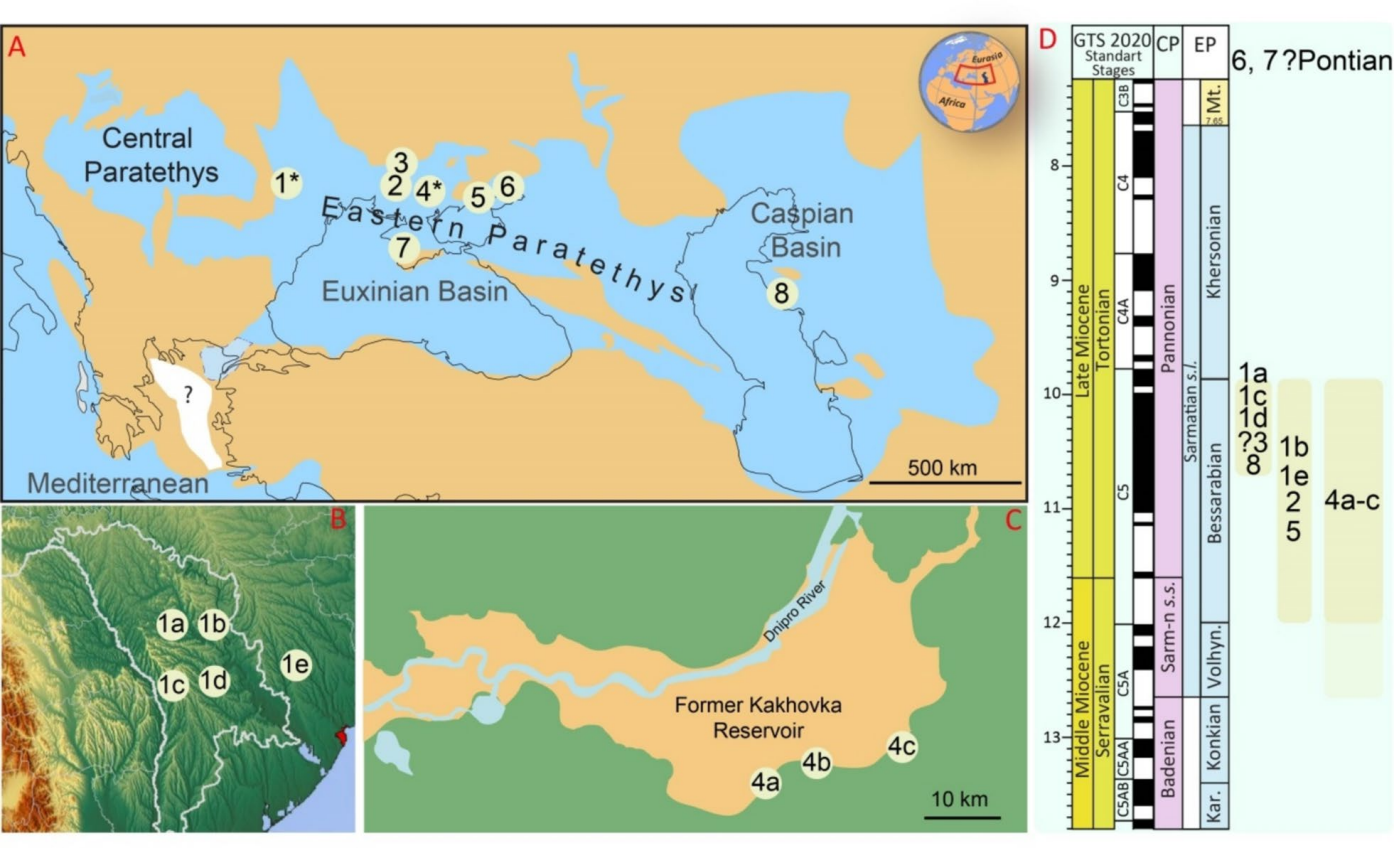
Geographic (**a–c**) and stratigraphic (**d**) distribution of *Paratethyphoca* gen. nov. and morphologically similar forms. Localities: 1*: *1a*—Verejeni (Moldova), *1b*—Mîrzeşti (Moldova), *1c*—Lăpuşna (Moldova), *1 d*—Chișinău (Hulbocica and Pruncul) (Moldova), *1e*—Vyshneve (Ukraine); *2*—Zolota Balka (Ukraine); *3*—Zhovtokamianka (Ukraine); 4*—left bank of the Kakhovka Reservoir (Ukraine): *4a*—Zlatopil, *4b*—Skelky, *4c*—Lysa Hora; *5*—Hnylozubovo (Ukraine); *6*—Khomutove (Ukraine); *7*—Kulykivka (Ukraine); *8*—Karagiye (Kazakhstan). **a** The map of Paratethys modified after Lazarev et al. (2025), **b, c** © Google.com, **d** redrawn from Lazarev et al. (2025)

This paper focuses on MCFFM V-150 (Fig. 1), an articulated skeleton discovered near Mîrzeşti, Orhei district, Republic of Moldova (Fig. 2). It includes a partially preserved skull, partial vertebral column, ribs, humeri, radius, distal ulna, metacarpals, femoral diaphysis, distal fibula, tarsal bones, metatarsals, and phalanges. The LSID for this publication is: urn:Isid:zoombank.org:pub:7BD 1BA75-66B F-4B43-B9BF-77C99CF2334 Comparative materials include both extinct and modern taxa (Table S1). They are stored in institutions of Austria, Denmark, Hungary, Moldova, Romania, Sakartvelo (Georgia), Slovakia, Switzerland, and Ukraine (the list of collections and specimens is provided in the Supplementary Table S1 and Table S2).

The specimens were surface scanned to obtain their volumetric models with a texture. The scans were made using the 3D surface scanner Artec Space Spider. Scans were processed by the Artec Studio 17 software under the Global registration and Sharp fusion algorithms to obtain 3D models, and Artec Studio 17 software texturing tools were used to add the texture to the models. In addition to processed materials, we used the photos of modern seal bones from the Idaho Virtual Museum (virtual.imnh.iri.isu.edu).

For measurements of the mandible, we used a modified scheme from Valenzuela-Toro et al. (2024) (Table S3). For manus and pes bones, we used their greatest length only (see Table S4). Seal humeri were measured according to the scheme by Ericson and Storå (1999), with the addition of the length of the humeral deltoid crest (see Table S5). Physical measurements were made using electronic callipers. For the pictures of bones from the Idaho Virtual Museum, we used Fiji software (Schindelin et al., 2012), and the tool in Artec Studio software was used for 3D model measuring.

The anatomical nomenclature follows that of Miocene seals (Dewaele et al., 2018; Koretsky, 2001) and the anatomy of domestic dogs (Budras et al., 2007; Nomina Anatomica Veterinaria, 2005). Lowercase letters refer to teeth in the lower jaw, whereas uppercase letters refer to teeth in the upper jaw.

For phylogenetic analysis, we used the character-taxon matrix from Otriazhyi et al. (2025), which in turn was modified from Dewaele et al. (2017, 2018), and Koretsky (2001). We also added two new characters (91, the presence or absence of the supraorbital process of the frontal, and 92, the presence or absence of the chin prominence of the mandible). We coded and added the skeleton of MCFFM V-150 and the holotype humerus NMNHU-P 64–701 of *Pachyphoca ukrainica.* For a supplementary analysis, the ilium of Phocidae NMNHU-P OF 1207 was added (see Phylogeny section below). In total, the morphological matrix included 35 taxa and 92 morphological characters (see the Supplementary File S1). In addition, we used matrices of four genes from Fulton and Strobeck (2010; FLVCR1, PNOC, RAG1, RAG2: Table S6 and supplementary materials).

Two approaches were used in the phylogenetic analysis: maximum parsimony and total evidence analysis with fossilised birth–death. The maximum parsimony analysis was made using the TNT 1.6 software (Goloboff & Morales, 2023). For the phylogenetic position of modern taxa, we used a constrained molecular tree from Fulton and Strobeck (2010), with the addition of the South American sea lion *Otaria flavescens* as an outgroup. The Wagner tree setting was run with 10,000 iterations, 10 trees saved from each, and implied weighting (k = 3). The results were outputted as frequency differences—GC. We used the STATS.RUN module to obtain the consistency index (CI) and retention index (RI).

The total evidence analysis (Ronquist et al., 2012) with fossilised birth–death (Heath et al., 2014) was run on BEAST 2.5 (Bouckaert et al., 2019) using packages sampled-ancestor 1.1.3 and morph-models 1.0.2. The Lewis Mk with no Gamma variation was used as the site model for morphological data, and four categories for the Gamma variation model with estimated *Shape* parameters were used for molecular data. The Relaxed Clock Log Normal model was chosen for both datasets (Drummond et al., 2006). For the tree prior distribution, we used a fossilised birth–death model with a starting value of 0.05 for the diversification rate. Also, we estimated the probability of an individual being sampled at present (Rho). The chain length was set at 15 million generations. The log was made every 1,000 trees. Two runs of the analysis were made. We visually checked the convergence of two runs in Tracer (Rambaut et al., 2018) for all parameters. The trees were summarized in TreeAnnotator 2.7.7.

For 3D morphometry, we used the generalized Procrustes analysis that was made using 3D slicer software (Fedorov et al., 2012) and its module SlicerMorph (Rolfe et al., 2021). Two groups of landmarks were placed on the humerus: the first included 19 true landmarks marking the main anatomical features of the bone (Table S7 and Fig. S1), whereas the second group included 15 semilandmarks equally spread on the curve from the greater tubercle through the deltoid crest and to the trochlea (Fig. S1). For the femur, three groups of landmarks were placed (Table S8 and Fig. S1): 19 true landmarks marking the main anatomical features, and two sets of 15 equally spaced semilandmarks each. One of the semilandmark sets was placed on the lateral curve from the distal edge of the greater trochanter to the distal end of the lateral epicondyle. Another set was placed on the medial edge of the femur from the narrowest point of the diaphysis to the distal end of the medial epicondyle. In the final analysis, in order to include the holotype of *Pachyphoca chapskii* NMNHU-P OF 1210 (a femur) in the analysis, we excluded four landmarks (##16–19) describing the shape of the femoral head. The measured material is listed in Table S9.

DNFO/RH ratio (the distance from the nasal foramen to the orbit/height of the rostrum at the rostral edge of the nasal).

## Geological settings of the studied material (Fig. 2)

Locality: Mîrzeşti, Republic of Moldova (1b in Fig. 2).

Approximate coordinates: 47.4988, 28.9999. The precise location of the outcrop from where the material described herein was collected is unknown.

Age/Stratigraphy: 12.05–9.90 Ma, late Middle—early Late Miocene, Bessarabian. The specimen was found on the Bessarabian shelly limestones yielding *Potamides taitboutii*, *Obsoletiformes* sp., and *Donax* sp.

Collection: MCFFM.

Referred specimens: MCFFM V-150 (Table S2).

References: authors’ data/observations presented in this study.

Locality: Hulbocica (Vesterniceni, Chișinău), Republic of Moldova (1 d in Fig. 2).

Approximate coordinates: 47.0556, 28.8575.

Age/Stratigraphy: 10.7–9.9 Ma, Late Miocene, Upper Bessarabian sensu Lazarev et al. (2025), based on the presence of the following taxa reported in Simonov and Zheru (1961): *Sarmatimactra fabreana*, *Sarmatimactra podolica*, and *Plicatiformes fittoni*.

Collection: MNEIN.

Referred specimens: MNEIN FN 144/n/a, FN 144/230b, 144–231, 144–132, 144–134, 144–135, 144–136 (Table S2).

References: Simonov and Zheru (1961); Dewaele et al. (2022).

Locality: Pruncul (Chișinău), Republic of Moldova (1 d in Fig. 2).

Approximate coordinates: 47.064280, 28.765024.

Age/Stratigraphy: 10.7–9.9 Ma, Late Miocene, Upper Bessarabian sensu Lazarev et al. (2025) based on the presence of *Sarmatimactra fabreana* according to Lungu and Rzebik-Kowalska (2011).

Collection: MNEIN.

Referred specimens: MNEIN FN 54 294 (Table S2).

References: Lungu and Rzebik-Kowalska (2011).

Locality: Lăpușna, Republic of Moldova (1c in Fig. 2).

Coordinates: 46.884616, 28.395203.

Age/Stratigraphy: 10.7–9.9 Ma, Late Miocene, Upper Bessarabian sensu Lazarev et al. (2025) based on the presence of *Plicatiformes fittoni* found in the layer with the studied bone (own observation and collection from the field). The section is composed of *Congeria*-bearing sandy-clayey deposits overlaid by sandy clays and sandstones with vertebrate remains and the shells of *Modiolus* sp. and *Sarmatimactra fabreana* (Vangengeim et al., 2006).

Collection: MNEIN.

Referred specimens: MNEIN n/a (Table S2).

References: Vangengeim et al., (2006); authors’ data/observations presented in this study.

Locality: Verejeni, Republic of Moldova (1a in Fig. 2).

Coordinates: 47.550837, 28.439765.

Age/Stratigraphy: 10.7–9.9 Ma, Late Miocene, Upper Bessarabian sensu Lazarev et al. (2025) based on the presence of *Barbotella hoernesi* (own observations).

Collection: MNEIN.

Referred specimens: MNEIN n/a.

References: authors’ data/observations presented in this study.

Locality: Lysa Hora (= Lysa Gora), Kakhovka Reservoir, Ukraine (4c in Fig. 2).

Approximate coordinates: 47.469465, 35.269913.

Age/Stratigraphy: uncertain, presumably 12.65–9.90 Ma, late Middle—early Late Miocene, from Volhynian to Bessarabian. The remains have been found along the coastline of the Kakhovka Reservoir. Our field observations in 2021 suggest that the base of the coastline is made of Volhynian age in situ clays. It is covered by brown loess-like sediments of aeolian-delluvial origin which includes reworks/transported blocks of Bessarabian age. Bessarabian age was earlier suggested for the sediments at the base of the section, covered by Khersonian sediments (Gol’din et al., 2012). The surface finds along the coastline most probably belong to the reworked Bessarabian rocks (Rekovets et al., 2014), although such an assumption remains speculative and additional finds with biostratigraphically relevant forms (mollusks, foraminifera) will be necessary to clarify this issue.

Collection: ZKM.

Referred specimens: ZKM P-245 (Table S2).

References: Gol’din et al. (2012); Rekovets et al. (2014); authors’ data/observations presented in this study.

Locality: Zlatopil, Kakhovka Reservoir, Ukraine (4a in Fig. 2).

Approximate coordinates: 47.412730, 35.018267.

Collection: ZKM.

Referred specimens: ZKM P-492, P-4950 (Table S2).

Comment: same as for Lysa Hora.

References: Gol’din et al. (2012); authors’ data/observations presented in this study.

Locality: Skelky, Kakhovka Reservoir, Ukraine (4b in Fig. 2).

Approximate coordinates: 47.449069, 35.119528.

Collection: ZKM.

Referred specimens: ZKM P-612 (Table S2).

Comment: same as for Lysa Hora.

References: Gol’din et al. (2012); authors’ data/observations presented in this study.

Locality: Zhovtokamianka (= Zheltokamenka), Ukraine (3 in Fig. 2).

Coordinates: 47.796441, 33.827005.

Age/Stratigraphy: probably 10.7–9.9 Ma, Late Miocene. Upper Bessarabian sensu Lazarev et al. (2025) based on the presence of the following taxa reported in Karlov (1940): *Sarmatimactra fabreana*, *Plicatiformes fittoni*. The age of the site needs confirmation since the superposition of the marine bivalve fauna to layer(s) with vertebrate remains has not been documented. In addition, the available bones have two different colors (brown and grey), suggesting the possible presence of two different fossiliferous layers.

Collection: NMNHU-P.

Referred specimens: NMNHU-P OF 1207, 1208, OF 1209, OF 1210, OF 1211, OF 1212, OF 1213, OF 1214, OF 1219; NMNHU-P 64–707, 64–711, 64–712 (Table S2).

References: Karlov (1940); Pidoplichko (1956).

Locality: Zolota balka (= Zolotaya Balka), Ukraine (2 in Fig. 2).

Approximate coordinates: 47.380719, 33.968992.

Age/Stratigraphy: Bessarabian age (12.05–9.90 Ma) has been suggested by Koretsky and Rahmat (2013). Since there are no publications presenting the geological description of the outcrop with biostratigraphically important faunal elements, this age should be adopted, although it requires a critical revision by visiting the outcrop.

Collection: NMNHU-P.

Referred specimens: NMNHU-P 64–348, 64–354, 64–471, 64–473, 64–477, 64–478, 64–479, 64–481, 64–482, 64–702, 64–703, 64–705, 64–712, 64–713 (Table S2).

References: Koretsky and Rahmat (2013).

Locality: Khomutove (= Homutovo), Ukraine (6 in Fig. 2).

Approximate coordinates: 47.254836, 38.137350.

Age/Stratigraphy: Pontian age has been suggested by Koretsky and Rahmat (2013), which corresponds to 6.1– ~ 5.3 Ma following Matoshko et al. (2023). Since there are no publications related to the geological description of the outcrop with biostratigraphically important groups, this age requires a critical revision. Considering the Bessarabian occurrences of other finds, this age stands out and it should be imperatively revised. As for now, the age of the specimens from this locality is considered by us as unknown.

Collection: NMNHU-P.

Referred specimens: NMNHU-P 64–701, 64–710, 64–383 (Table S2).

References: Koretsky and Rahmat (2013).

Locality: Kulykivka (= Kulikovka), Ukraine (7 in Fig. 2).

Approximate coordinates: 45.216353, 33.608905.

Age/Stratigraphy: Pontian age (6.1- ~ 5.3 Ma) has been suggested by Koretsky (2001). However, considering the Bessarabian occurrences of other finds, this age stands out and it should be imperatively revised. As for now, the age of the specimens from this locality is considered by us as unknown.

Collection: ONU, NMNHU-P.

Referred specimens: NMNHU-P 64–140 (Table S2).

References: Koretsky (2001).

Locality: Vyshneve (= Kirovo), Ukraine (1e in Fig. 2).

Approximate coordinates: 47.196000, 30.086000.

Age/Stratigraphy: Bessarabian age (12.05–9.90 Ma) has been suggested by Koretsky and Rahmat (2013). Since there are no publications related to the geological description of the outcrop with biostratigraphically important faunal elements, this age should be adopted as referred to, however, requiring a critical revision by visiting the outcrop.

Collection: ONU, NMNHU-P.

Referred specimens: ONU 3721 (2, 5, 7, 8, 10), ONU 3720 (1, 2, 3, 6, 11, 14, 16, 17), ONU 3719 (1, 2, 6, 10, 11, 12, 16) (Table S2).

References: Koretsky (2001).

## Results

### Systematic paleontology

Class Mammalia Linnaeus, 1758

Order Carnivora Bowditch, 1821

Family Phocidae Gray, 1821

Subfamily Phocinae Gray, 1821

Genus ***Paratethyphoca***, gen. nov.

urn:lsid:zoobank.org:act:C31D0DE8-78A2-479A-B1AB-5FC02DD67BB4

### Type species

*Paratethyphoca libera*, sp. nov.

urn:lsid:zoobank.org:act:1067DD76-C4D8-446F-BF3B-48B68736113B

### Diagnosis

Same as for the type species.

### Etymology

The genus name derives from Greek words—“Paratethys”, the name of the epicontinental sea where the animal lived, and “Phoca” meaning “a seal”. The species name derives from the Latin word meaning “free”, which is the sign of the freedom of taxonomic thought supported by the newly found specimen described herein.

**Species** ***Paratethyphoca libera*** sp. nov.

**Holotype: MCFFM V-150**

**Type Locality:** Mîrzeşti, Moldova (approximate coordinates 47.49885 N, 28.99998 E).

**Type Horizon:** Bessarabian shelly limestones.

**Age/Stratigraphy:** 12.0–9.9 Ma, Bessarabian (late Middle—early Late Miocene) of Eastern Paratethys.

### Diagnosis

*Paratethyphoca libera* belongs to Phocinae in having (1) an ascending process of the premaxilla outside the nasal cavity and visible laterally, (2) a great development of the humeral supinator, and (3) a trochlea of humerus larger than a capitulum. It differs from all Monachinae in a pointed (rather than flattened) distal end of the styloid process of the ulna, and the metatarsal III longer than half of the metatarsal I.

*Paratethyphoca libera* differs from all Phocinae in the presence of a supraorbital process of the frontal bone in the posterior portion of the bone, a unique autapomorphic feature (other Phocinae lack it, or, in the case of *Cystophora cristata*, have it in the anterior portion of the frontal). It further differs from all Phocinae in a proportionally long humerus (as long as 88% of the skull length).

*Paratethyphoca libera* also differs from most Phocinae but *Monachopsis pontica* in its long snout (as long as the orbits). It differs from all crown Phocinae except *Erignathus barbatus* in having a well-developed chin prominence of the mandible (also shared by *Monachopsis pontica*). It is distinguished from all Phocinae except some of the Paratethyan seals (*e.g.*, NMNHU-P 64–707, ONU 3721, ZKM P-612), in a shallow supraspinous fossa of the scapula (20% of the bone length from the glenoid fossa to the dorsal margin), and further differs from NMNHU-P 64–707 in a convex cranial scapular edge.

*Paratethyphoca libera* also differs from most Phocinae in several features of its humerus: a humeral lesser tubercle below the level of the humeral head (also shared by *Devinophoca* spp., *Praepusa* spp., *Cryptophoca maeotica* NMNHU-P 64–530, *Pontophoca sarmatica* NMNHU-P 64–1713/10*, Pachyphoca ukrainica* NMNHU-P 64–701, *Phoca bessarabica* AICUPM SF-3); a smooth distal termination of the deltoid crest (contrary to a sharp distal termination) (shared by *Pachyphoca ukrainica*, *Pontophoca sarmatica*, and *Devinophoca* spp.), and it further differs from other Paratethyan seals (except for *Pachyphoca ukrainica* and *Pontophoca sarmatica*) in a deltoid crest distally reaching only half of the bone length.

### Description

#### Skull (Fig. 3)

**Fig. 3:**
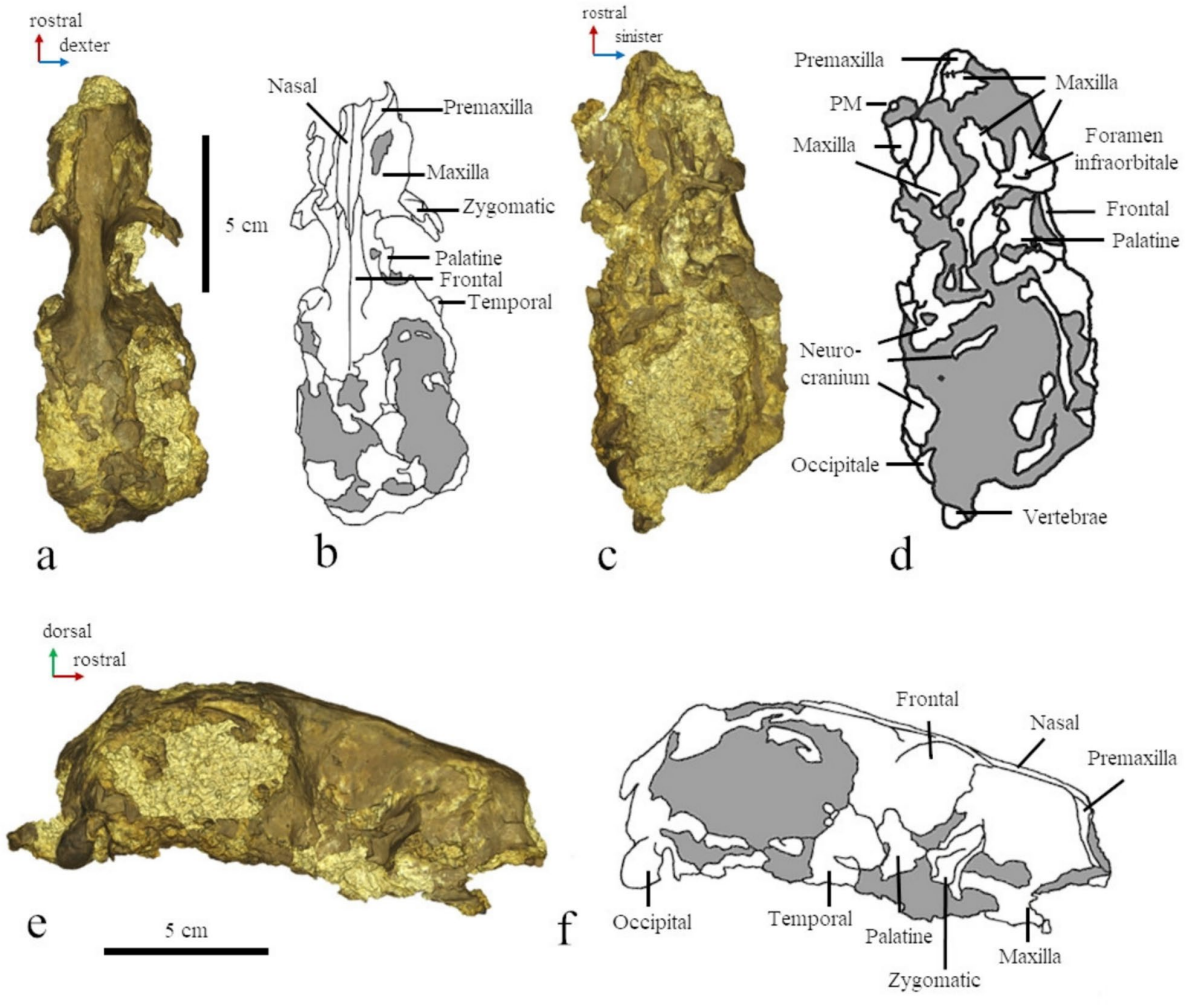
The skull of MCFFM V-150 *Paratethyphoca libera*. **a** Dorsal view (3D model); **b** dorsal view (outline with labeled bones; the matrix is shown in grey); **c** ventral view (3D model); **d** ventral view (outline with labeled bones; the matrix is shown in grey); **e** right lateral view (3D model); **f** right lateral view (outline with labeled bones; the matrix is shown in grey)

MCFFM V-150 preserved the nasal, frontal, and maxillary bones, with partially preserved palatal and alveolar processes. Also, it includes the nasal process of the premaxilla, an anterior fragment of the jugal, and the damaged neurocranium. The parietal, lateral occipital (mostly the occipital condyles), squamosal (mostly the mandibular fossa) and tympanic bullae are also partially preserved.

The length of the skull (from the base of the occipital condyle to the nasal cavity) is 128 mm. The DNFO/RH ratio (distance from the nasal foramen to orbit/height of the rostrum at the rostral edge of the nasal) equals 130%. The snout, measured from the orbit to the nasal cavity, is approximately as long as the orbit.

The nasal has a pointed posterior edge. It transversely expands both posteriorly and in its anteriormost part. The posterior end of the nasal is located posteriorly to the maxilla-frontal suture. The nasal is 11.3 times longer than its medial width (the length is 42.0 mm, and the width of the nasal is 3.8 mm).

The premaxilla-nasal suture is short (7.1 mm). The palatal process of the maxilla is slightly concave ventrally. The maxilla has a small antorbital process.

The interorbital minimum width (7.2 mm) is about 10% of the braincase width, and it is located in the anterior half of the interorbital region. The frontal has a transversely short supraorbital process, and its maximum width is 14.6 mm.

#### Mandible (Fig. 4)

**Fig. 4:**
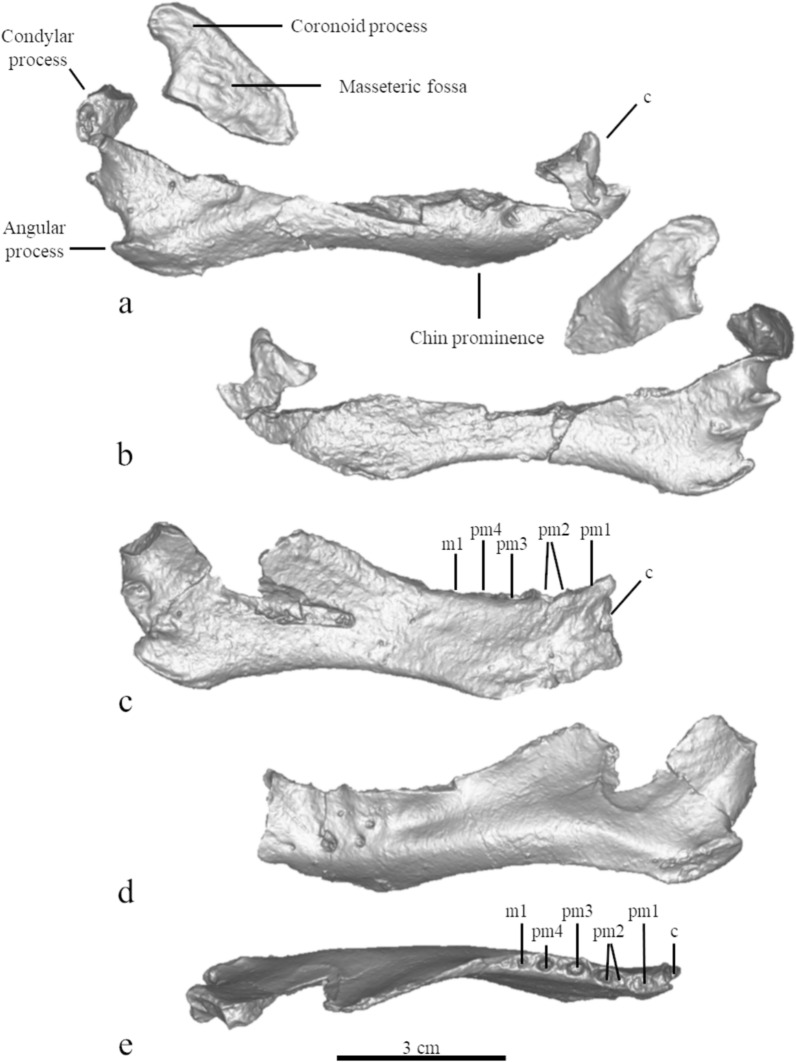
The mandible of MCFFM V-150 *Paratethyphoca libera.* Right ramus of the mandible in lateral (**a**) and medial (**b**) view; left ramus of the mandible, in medial (**c**), lateral (**d**) and labial (**e**) view

The bone has a well-developed chin prominence, anteriorly to which the height of the mandible becomes lower and then expands again. Hence, the ventral margin of the mandible is concave between the chin and the angular process from the lateral view. The masseteric fossa has two longitudinal ridges. The condylar and angular processes are well developed, and both are about 3.5% of the bone length. The minimum width of the coronoid process is 10% of the bone length, and its dorsal edge is rounded (rather than pointed). The mandibular foramen is long and narrow, and it anteriorly reaches the mid-point between the angular process and the chin prominence.

Based on the alveoli, the mandible has a single-rooted pm1. The pm2-pm4 are double-rooted. The m1 has a smaller diameter (2.6 mm) compared to that in the pm4 (3.2 mm), although its alveolus is poorly preserved. The anteriormost mental foramen is located beneath the level of the alveolus of the pm1, the second mental foramen is positioned under the anterior part of the pm2, and the third one is situated under the pm3 (Fig. 4 and Table S3).

#### Dentition (Fig. 5)

**Fig. 5:**
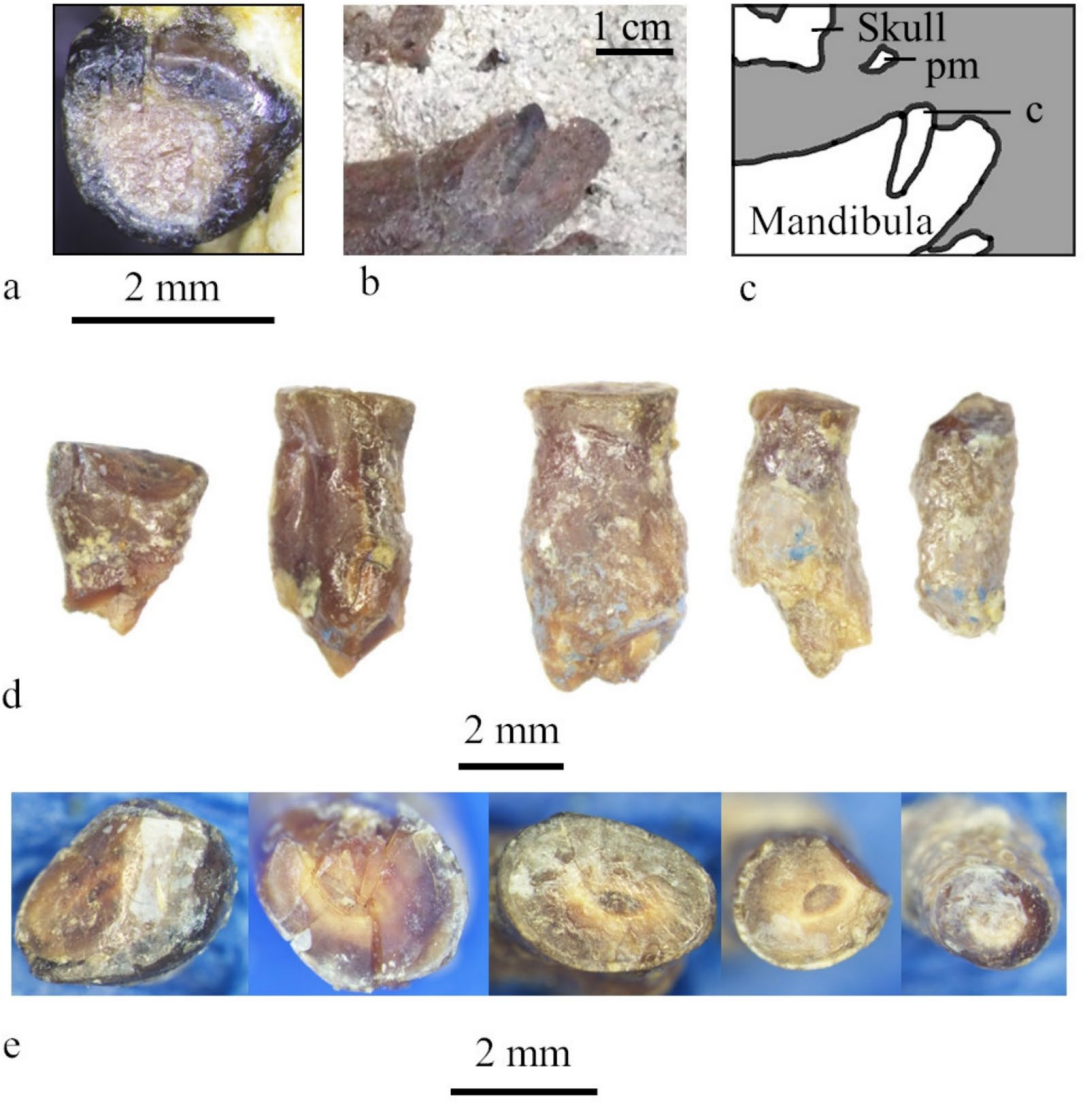
Dentition of MCFFM V-150 *Paratethyphoca libera.* **a** The maxillar premolar (PM1 or PM2), occlusal view; **b, c** left ramus of the mandible of MCFFM V-150 with the canine in the matrix, medial view; photo (**b**) and bones outline (**c**), the matrix shown in grey; **d** roots of lower postcanine teeth found in the matrix near the mandible, lateral view; **e** the same roots of lower postcanine teeth, occlusal view.

Four fragmentary mandibular teeth were found in the matrix, and only one tooth (probably PM1 or PM2) was found intact in the maxilla. All teeth have no preserved crowns; instead, they are severely worn and have flat occlusal surfaces at the level of the tooth neck (Fig. 5).

#### Vertebrae (Fig. 6)

**Fig. 6:**
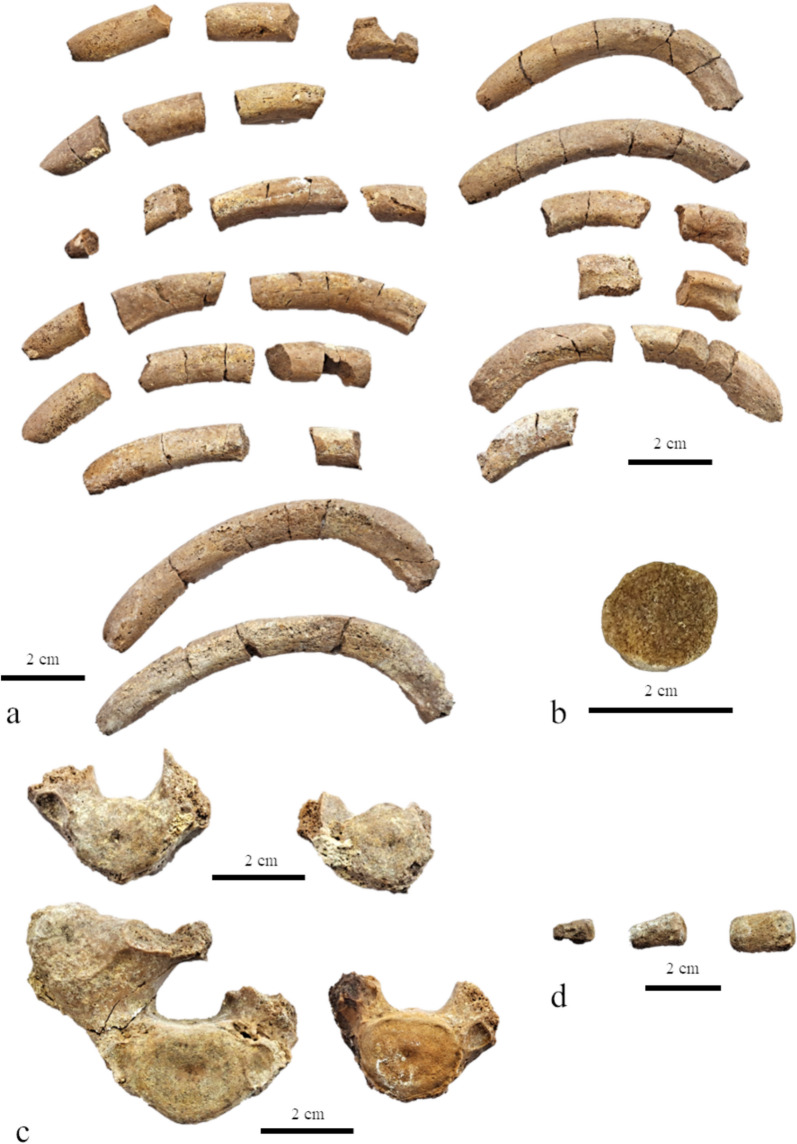
Ribs and vertebrae of *Paratethyphoca libera* MCFFM V-150. **a** Ribs; **b** a cross-section of the rib; **c** a thoracic vertebra; **d** caudal vertebrae. Scale bars equal 2 cm

The middle thoracic vertebrae have massive transverse processes, which are as high as the centra. Among the preserved vertebrae, there are three cervical (embedded in the matrix), eight thoracic (four of which embedded in the matrix), and three caudal vertebrae.

#### Ribs

The bones are thick, up to 25 mm wide, very robust and pachyosteosclerotic, mostly circular at the cross-section in their middle to distal portions (Fig. 6).

#### Scapula (Fig. 7)

**Fig. 7:**
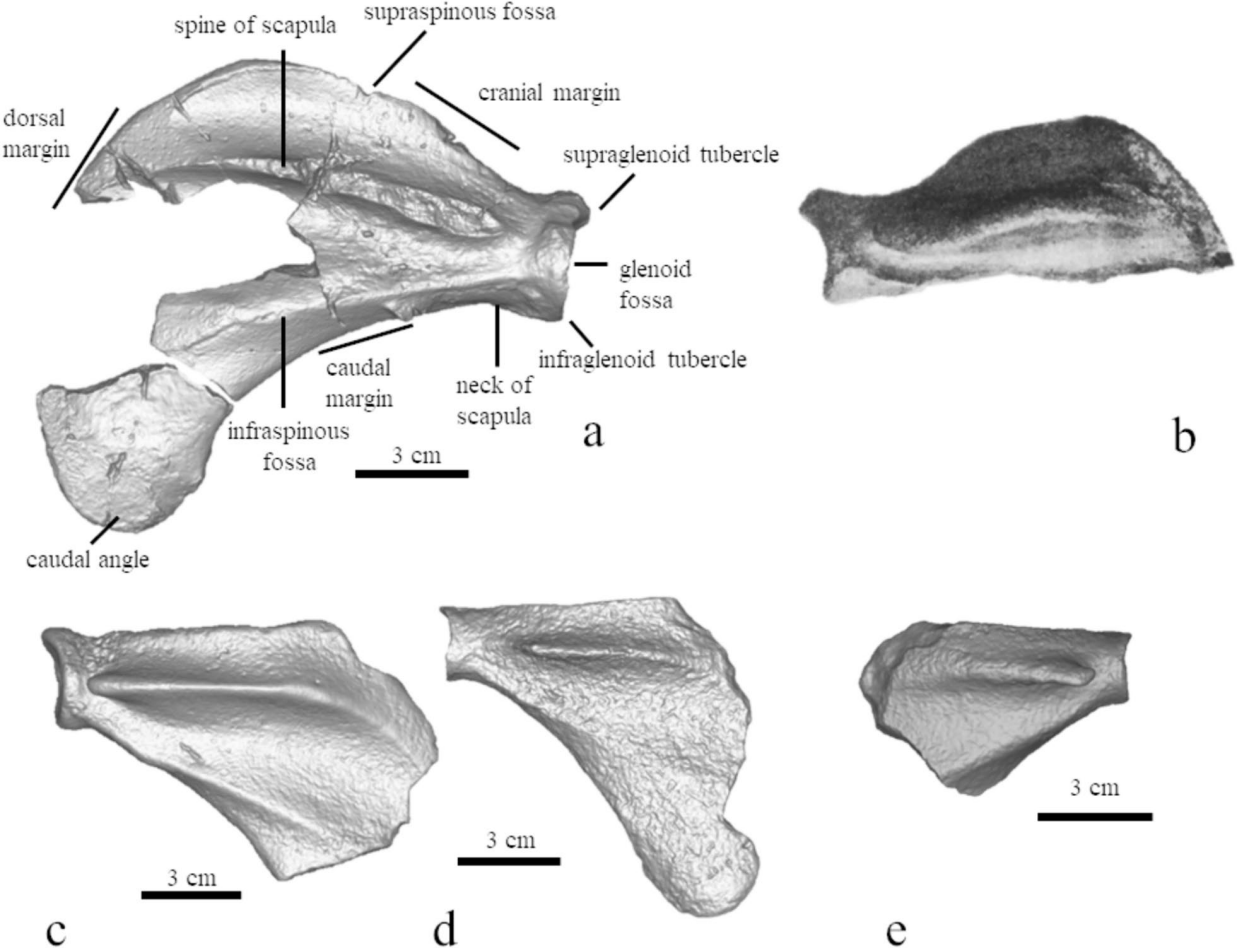
Comparison of seal scapulae, posterior view. **a** MCFFM V-150 *Paratethyphoca libera*; **b** “*Phoca sarmatica*”. From Alekseev, 1924a, 1924b II, **c** NMNHU-P 64–707. **d** NMNHU-P 64–477. **e** MNEIN n/a from Lăpușna 4

The bone is robust and thick. The height of its neck is about 90% of the glenoid fossa. The width of the articulated surface is about 85% of its height. Two scapular ridges on the lateral side of the bone do not join near the glenoid. The supraspinous fossa is low, and its height is about 20% of the bone length from the glenoid fossa to the dorsal margin. The cranial margin of the scapula is slightly convex.

#### Humerus (Figs. 8, 9, 10)

**Fig. 8:**
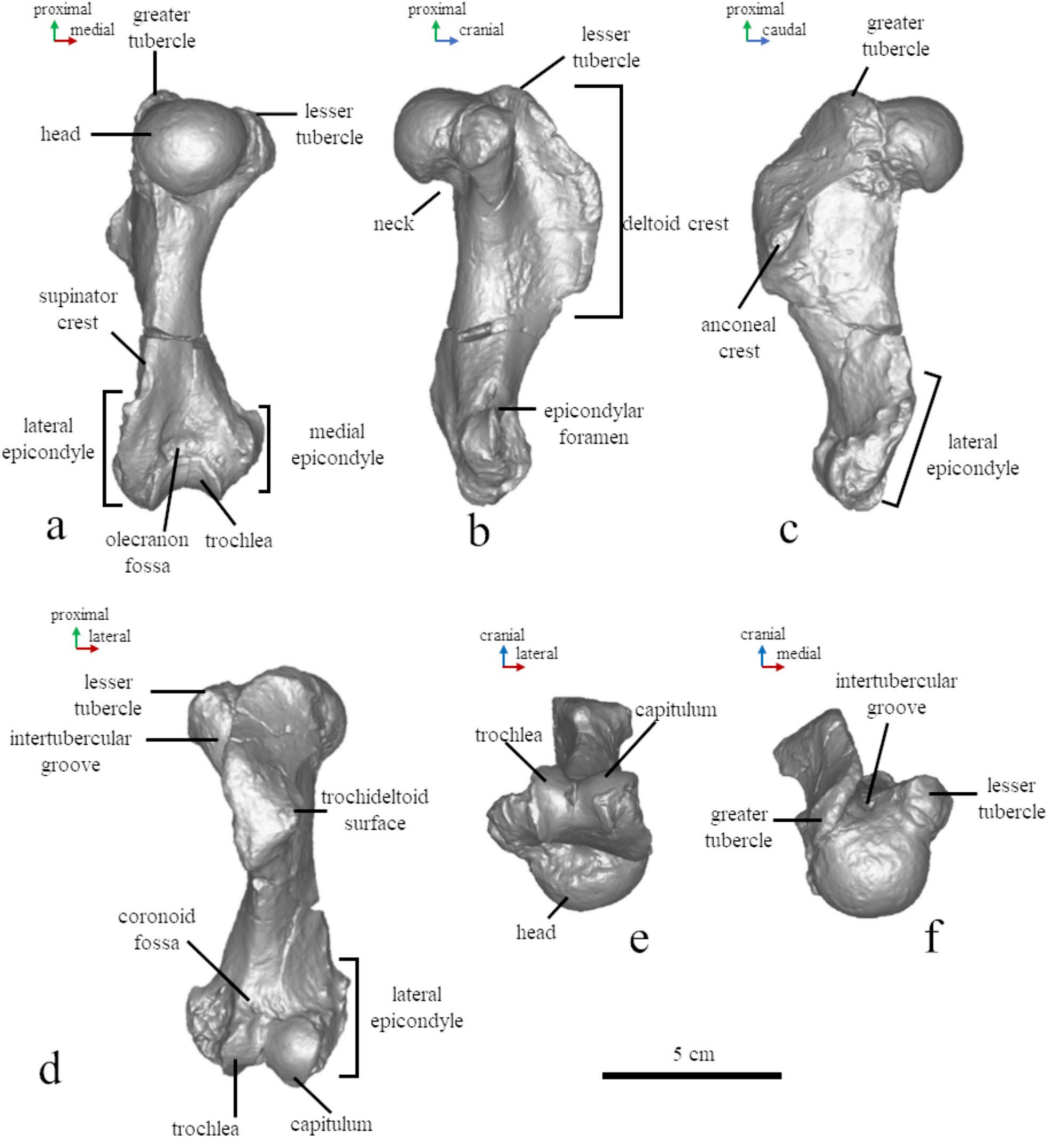
Osteological nomenclature of the humerus (MCFFM V-150) (**a–f**). Digital models of the bones have been used in posterior (**a**), medial (**b**), lateral (**c**), anterior (**d**), inferior (**e**), and superior (**f**) views

**Fig. 9:**
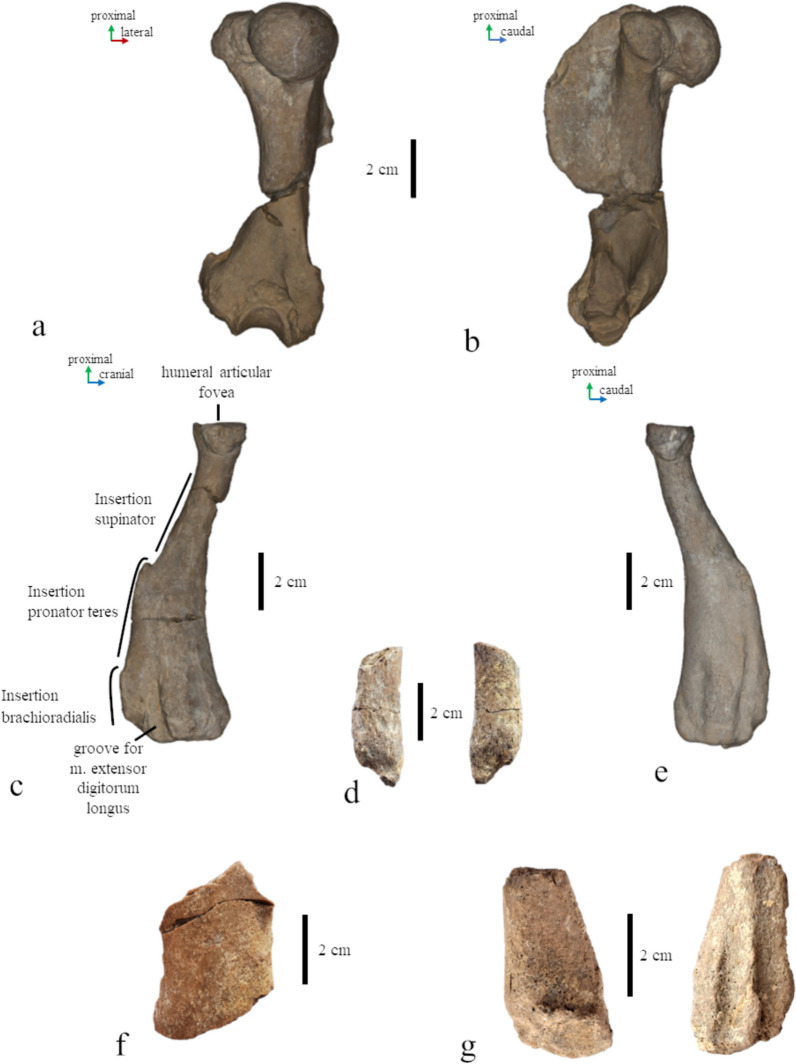
Limb bones of *Paratethyphoca libera*, **a, b** right humerus of MCFFM V-150, in posterior (**a**) and medial (**b**) view; **c** radius of MCFFM V-150, in lateral view; **d** distal part of the ulna MCFFM V-150; **e** radius FN 144–230, in lateral view; **f** femoral diaphysis of MCFFM V-150; **g** distal part of the fibula of MCFFM V-150. Scale bars equal 2 cm

**Fig. 10:**
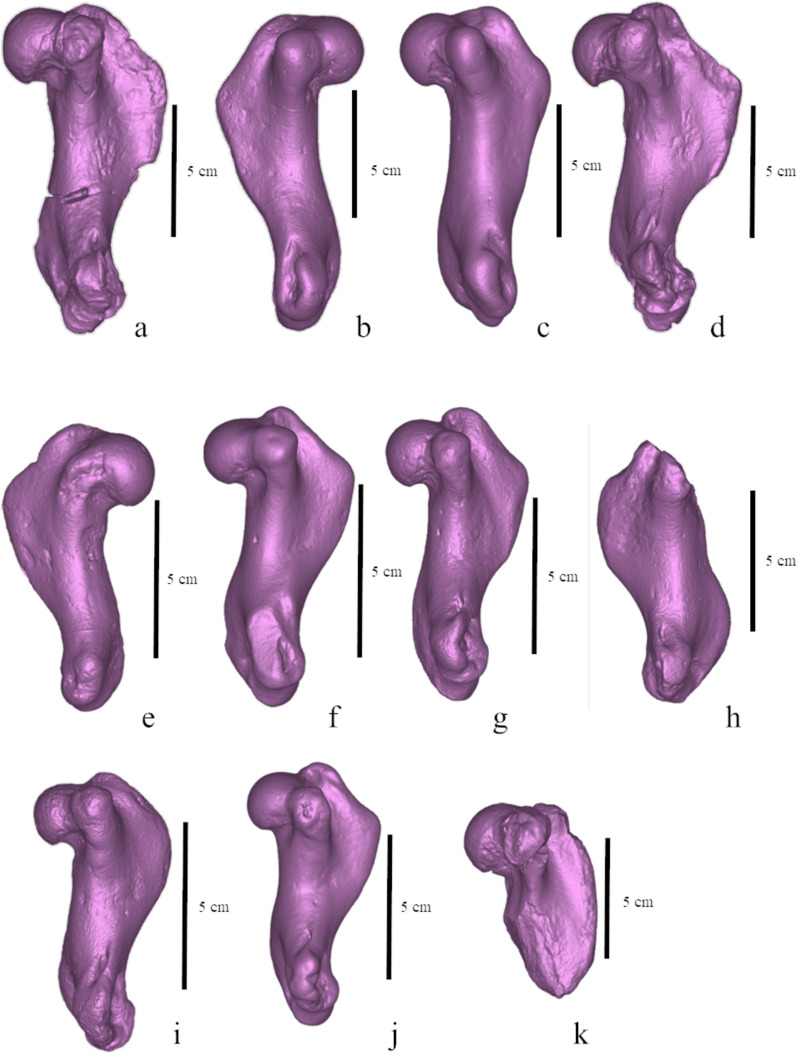
Humeri of pachyosteosclerotic seals from the Paratethys, lateral view. **a** *Paratethyphoca libera* MCFFM V-150; **b** Phocidae NMNHU-P OF 1209; **c** Phocidae ZKM P-492 4; **d** *Pontophoca sarmatica* NMNHU-P 64–1713/10; **e** Phocidae NMNHU-P OF 1208; **f** Phocidae ZKM P-492 17 (1–4); **g** Phocidae ZKM P-470; **h** Phocidae FN 144-n/a. **i** *Pachyphoca ukrainica* NMNHU-P 64–701 (holotype); **j** Phocidae ZKM P-492 (1–4) a; **k** “*Phoca” bessarabica* SF-3 (holotype). Scale bars equal 5 cm

The total length of the humerus reaches 88% of the length of the skull measured from the nasal cavity to the base of the occipital condyle. This is extraordinary because other phocid seals usually have a shorter humerus—less than 80%, and only in *Erignathus barbatus* it measures 83% of the skull length (PO, personal observation). The head of the humerus is slightly flattened proximodistally. The proximal edge of the deltoid crest is slightly convex, and the caudal one is slightly concave. The proximal and caudal edges form an angle of 125°. The deltoid crest has a smooth distal transition to the diaphysis, which begins in the middle of the bone. The highest point of the deltoid crest is as high as the humeral head. The greater tubercle extends by level of the humeral head, and the lesser one is below the head. The intertubercular groove is shallow and wide. The anconeal crest is low. The lateral epicondyle is twice as large as the medial one, and it is about 35% of the humerus length. The supinator crest is slightly bulged caudally. The olecranon fossa is deep and narrow. The trochlea is two-third of the humeral head width (Figs. 8, 9, 10).

#### Radius (Fig. 9)

The radius is generally narrow, and the greatest width of its distal part equals 28% of the bone length. The pronator teres insertion is located proximally. The radial tuberosity is on the posteromedial side of the bone. The **ulna** (Fig. 9) has a distally pointed end of the styloid process.

#### Femur (Figs. 9, 11)

**Fig. 11:**
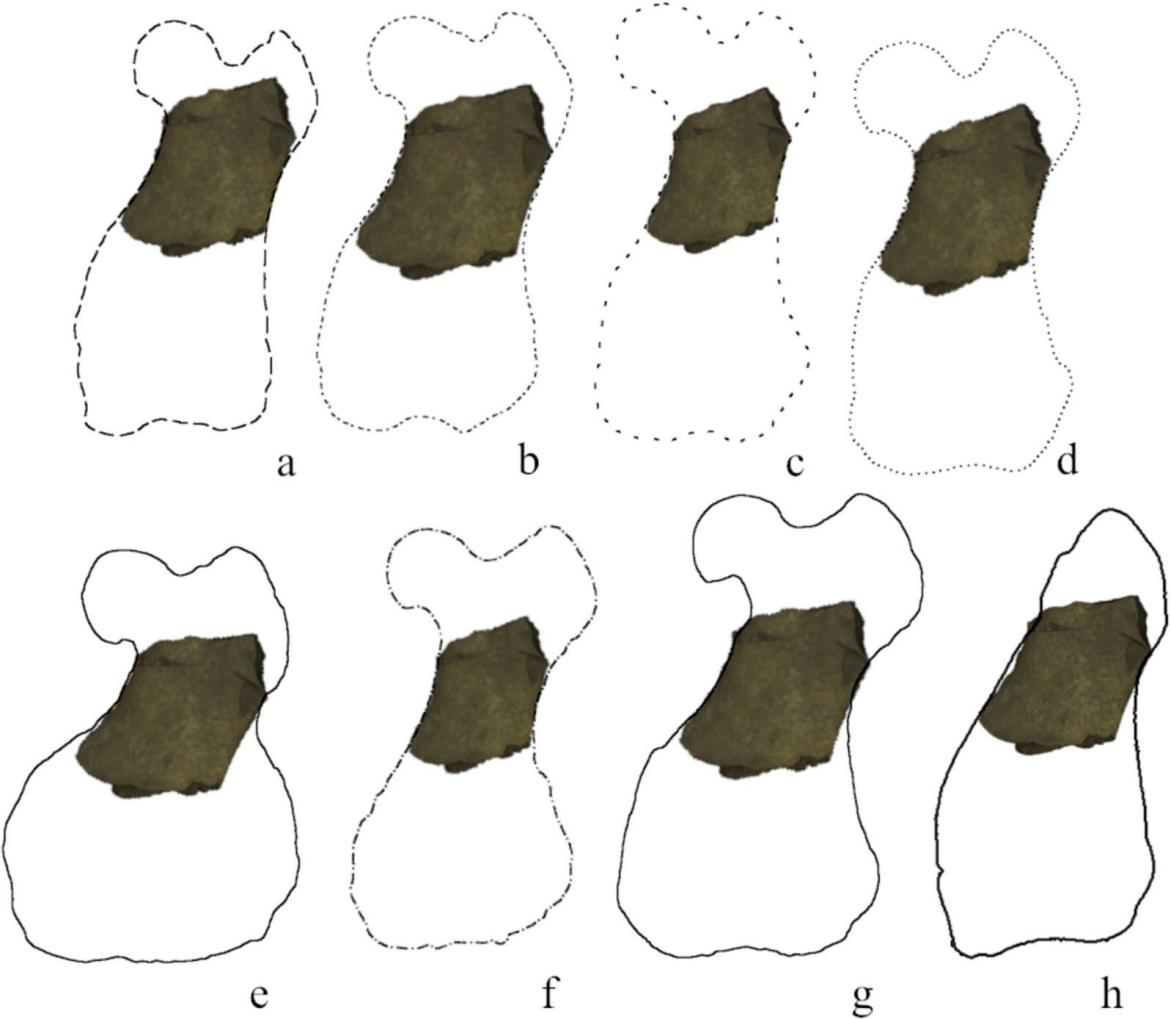
Comparison of the femur *Paratethyphoca libera* MCFFM V-150 with outlines of femora of other Paratethyan seals adjusted to the same width of diaphysis. **a** Phocidae MNEIN FN 54 294; **b** Phocidae NMNHU-P 64–354; **c** *Cryptophoca maeotica* MNEIN FN 144–133 (**d**); Phocidae AICUPM MS-50; **e** *Pontophoca sarmatica* NMNHU-P 64–1713/10; **f** *Cryptophoca maeotica* NMNHU-P n/n; **g** *Cryptophoca maeotica* NMNHU-P 64–1713/23; **h** Phocidae NMNHU-P OF 1210

The diaphysis is the only preserved part of the femur. Its mediolateral width is 29.0 mm, whereas the craniocaudal width is 17.0 mm; the bone is composed of compact bone tissue, and its medial and lateral edges are slightly concave.

#### Cuboid

Its surface has a medial process for the third cuneiform bent distally (Fig. S2).

The measurements of the **manus and pes bones** are shown in Table S4 and Fig. S3.

## Comparison with the possibly closely related Miocene taxa

***Pontophoca sarmatica (***Alekseev, 1924a, 1924b**), NMNHU-P 64–1713/10.** The original description of *Pontophoca sarmatica* includes illustrations of the scapula, coxae, and femur. The low and slightly convex cranial margin of the scapula is similar to that in *Paratethyphoca libera*. The femur was chosen as the lectotype by Koretsky and Ray (1994) based on the presence of autapomorphies (extremely wide medial and lateral epicondyles). However, the location of the material is currently unknown. The humerus and femur NMNHU-P 64–1713/10 have been reported as belonging to the same individual of *Pontophoca sarmatica* Koretsky & Grigorescu, 2002), so they were selected by us for comparison in the present study. The femur has an identical shape as the holotype (femur) of *Pontophoca sarmatica*, originally illustrated in Alekseev (1924b).

The humerus NMNHU-P 64–1713/10 has a very similar shape as *Paratethyphoca libera* (Fig. 10). The only minor difference is that the trochlear crest of *Paratethyphoca libera* is concave between the trochlea and capitulum, whereas it is convex in NMNHU-P 64–1713/10. The trochlea of *Paratethyphoca libera* is as wide as 61% of the humeral head width, whereas it equals 77% in NMNHU-P 64–1713/10. The proximal bifurcation of the deltoid crest is lacking in *Paratethyphoca libera*, while it is slightly developed in NMNHU-P 64–1713/10. The diagnostic value of these differences is unclear.

The femur of *Pontophoca* has extremely large medial and lateral epicondyles, which also strongly impact the shape of the diaphysis. The latter is narrow in its middle portion (in contrast to the wide diaphysis in *Paratethyphoca libera*). The lateral and medial edges of the diaphysis in *Paratethyphoca libera* are slightly concave and almost parallel, whereas the lateral edge of the diaphysis in the femur NMNHU-P 64–1713/10 is strongly concave, and both edges diverge distally (see Fig. 11, S5 with bones outlined for more details). Therefore, there are minor differences between the humeri of *Paratethyphoca libera* (MCFFM V-150) and NMNHU-P 64–1713/10, although they may be important. Contrary to that, differences in the shape of the femur are great, even considering only a fragment is preserved from *Paratethyphoca libera*. Better preserved and articulated skeletons of both species are necessary to understand the range of variation and differences between these taxa. As for now, based on the differences of the femoral morphology, we consider them as members of different genera. Moreover, we suggest *Pontophoca sarmatica* as a valid genus and species name based on strong autapomorphic features of its femur, as suggested by Kretzoi (1941) and Koretsky and Grigorescu (2002).

***Cryptophoca maeotica (***Nordmann, 1860**), NMNHU-P 64–455, 64–530.** A Paratethyan seal known from the Bessarabian of the Eastern Paratethys (Chișinău, Moldova; Kamysh-Burun, Töbeçik, Ukraine) (Koretsky, 2001; Koretsky & Ray, 1994). The holotype is the femur HMZ 1815, which is morphologically identical to NMNHU-P 64–455. The type series also includes the humerus HMZ 1812.

The deltoid crest of the humerus is shorter in *Paratethyphoca libera* and measures a half of the bone length. It is significantly longer (65%) in *Cryptophoca maeotica* NMNHU-P 64–530. The ratio of the lateral epicondyle length to bone length in MCFFM V-150 and *Cr. maeotica* is 28% and 37%–40%, respectively. *Paratethyphoca libera* has a triangular deltoid crest (in lateral view), and its transition to the diaphysis is smooth. In contrast, the deltoid crest in *Cr. maeotica* NMNHU-P 64–530 is rounded, and its transition to the diaphysis is sharper (Fig. 10). The femur of *Paratethyphoca libera* has slightly concave lateral and medial edges, while in *Cr. maeotica* NMNHU-P 64–455, only the lateral edge of the diaphysis is concave (Fig. 11, S5). We suggest that these specimens represent different taxa. However, due to the lack of representative specimens, we cannot conclude if *Cr. maeotica* may be closely related to *Paratethyphoca libera* and if *Cryptophoca maeotica* is a valid name, and leave this question open.

***Phoca bessarabica ***Simionescu, 1925**, AICUPM SF-3, SF-5.** The original description of the type material includes three bones: the proximal part of the left humerus AICUPM SF-3, almost complete pelvis without an ischium, and the fifth metatarsal AICUPM SF-5. This material comes from the Bessarabian deposits exposed near Chișinău (Simionescu, 1925). Both seals were relatively large, being among the largest known Paratethyan seals. *Paratethyphoca libera* has the humeral head wider than high, and its deltoid crest is well-developed and triangular in shape. In contrast, the humeral head of *Phoca bessarabica* AICUPM SF-3 is higher than wide, and its deltoid crest is low and rounded (Fig. 10). The fifth metatarsals of MCFFM V-150 and AICUPM SF-5 do not have clear differences (Fig. S4), although the diagnostic value of this bone is under question. Therefore, we suggest these forms may belong to the same or a closely related taxon at least at the genus level. However, the type material of *Phoca bessarabica* is too fragmentary for a certain conclusion, and currently we agree to consider this name a *nomen dubium* (cf. Rule et al., 2024).

***Pachyphoca ukrainica ***Koretsky & Rahmat, 2013**, NMNHU-P 64–701 (holotype).** The type specimen is the left humerus, with a slightly eroded surface. In addition, based on the description of *Pachyphoca volkodavi* from the north-western Caucasus, also represented by an isolated humerus (Tarasenko & Titov, 2022), we consider it as fully identical to *Pachyphoca ukrainica* and being its junior synonym.

*Paratethyphoca libera* has a longer humerus than *Pp. ukrainica* (115.0 mm vs. 85.0 mm). It also has a greater tubercle of the humerus reaching the level of the humeral head, whereas *Pp. ukrainica* (NMNHU-P 64–701) has a greater tubercle higher than the humeral head. In *Paratethyphoca libera,* the proximal edge of the deltoid crest is slightly convex*,* and the deltoid crest itself is angular. In contrast, *Pp. ukrainica* has a slightly concave proximal edge of the deltoid crest, and the deltoid crest itself is rounded. However, these features of *Pp. ukrainica* could be related to the taphonomic alteration of the specimen. The intertubercular groove is narrow in *Paratethyphoca libera* but wide in *Pp. ukrainica*.

We found some differences between these forms but can hardly distinguished the isolated humerus (holotype) of *Pachyphoca ukrainica* from *Paratethyphoca libera*, as well as from the specimens previously assigned to *Pontophoca sarmatica* and *Cryptophoca maeotica* (Figs. 10, S8). In our opinion, the humeri of this pool of taxa are poorly diagnosable, and their measurements mostly overlap; also, refer to *Generalized Procrustes Analysis* below. Therefore, as suggested by Rule et al. (2024), we agree to consider the names *Pachyphoca ukrainica* and *Pachyphoca volkodavi* and, subsequently the name *Pachyphoca* as *nomina dubia* (Rule et al., 2024). However, we have found close affinities of this seal to *Paratethyphoca libera* possible, considering the shared character states such as the deltoid crest as long as a half of the humerus length, a lesser tubercle below the proximal edge of the humeral head, and a smooth distal transition from the deltoid crest to the diaphysis.

**Other specimens from Khomutove, Ukraine** (*Pachyphoca ukrainica* sensu Koretsky & Rahmat, 2013), ulnae NMNHU-P 64–383, 64–710 (Table S2). In addition to the holotype of *Pachyphoca ukrainica,* only two ulnae are known from the site, and the ulna NMNHU-P 64–710 may belong to the same individual as the holotype humerus NMNHU-P 64–701. The overlap with MCFFM V-150 is limited by the distal part of the bone and not showing differences. However, the ulnae from Khomutove have a short olecranon, a rare characteristic which may help comparing *Paratethyphoca libera* and seals from Khomutove in future.

***Pachyphoca chapskii ***Koretsky & Rahmat, 2013**, NMNHU-P OF 1210 (old number 64–706) (holotype)** (Fig. S6). The specimen is an isolated incomplete femur with a missing head. The medial edge of the diaphysis from the proximal edge of the medial epicondyle to the femoral neck is straight (rather than concave). The lateral and medial edges of the diaphysis are under an angle of 35° to each other (in which it differs from *Paratethyphoca libera,* which has these edges almost parallel). The lateral epicondyle doesn’t protrude laterally with a barely noticeable transition to the bone diaphysis. The suprapatellar fossa is well-pronounced and smoothly transits to the patellar facet. As suggested by Rule et al. (2024), we consider the name *Pachyphoca chapskii* as *nomen dubium* due to the incompleteness of the type specimen, which is hardly comparable to any known seal. However, this seal can also be closely related to *Paratethyphoca libera*. Additionally, the humeri from Zhovtokamianka resemble those of MCFFM V-150 in shape and size (see below). A direct comparison and re-evaluation may be possible after finding better preserved specimens.

**Other specimens from Zhovtokamianka, Ukraine** (*Pachyphoca ukrainica* and *Pachyphoca chapskii* sensu Koretsky & Rahmat, 2013), NMNHU-P 64–707, OF 1208, OF 1209 (Table S2). Pachyosclerotic and robust bones from this locality were assigned by Koretsky and Rahmat (2013) to two species of *Pachyphoca;* the differences between the two alleged morphotypes have been reported mostly in bone size. The scapula of *Paratethyphoca libera* MCFFM V-150 has a higher convex cranial margin, while the cranial margin in NMNHU-P 64–707 is low (not higher than the glenoid) and straight. The humerus of MCFFM V-150 is slightly smaller (113.0 mm) than NMNHU-P OF 1209 (120.0 mm) but larger than NMNHU-P OF 1208 (89.0 mm). The lesser tubercle is below the level of the head in both MCFFM V-150 and NMNHU-P OF 1209, and higher in NMNHU-P OF 1208 (Fig. 10). The deltoid crest is equally short in MCFFM V-150 (50%) and in seals from Zhovtokamianka (55% in OF 1208 and OF 1209). The ulnae of MCFFM V-150 and the specimens from Zhovtokamianka (NMNHU-P 64–711, 64–712, OF 1214, OF 1213) have a distally pointed end of the styloid process. Therefore, we suggest these specimens can be closely related to *Paratethyphoca libera.*

**Seals from Zolota Balka** (*Pachyphoca ukrainica* sensu Koretsky & Rahmat, 2013), NMNHU-P 64–354, 64–477, 64–481, 64–482, 64–702, 64–705 (Table S2, Fig. S6). *Paratethyphoca libera* MCFFM V-150 has a convex cranial margin of the scapula, and the caudal margin is more concave. In NMNHU-P 64–477 and 64–702, the cranial margin is straight and lower than the glenoid fossa, whereas the caudal margin is mostly straight. The radius of MCFFM V-150 is larger (111.0 mm) than that in NMNHU-P 64–481 (75.0 mm) while both bones are narrow. Their maximum width equals 28% of the bone total length. The ulna NMNHU-P 64–705 has a short olecranon as in NMNHU-P 64–710 from Khomutove. Also, the femur of MCFFM V-150 has a similar shape to the femur NMNHU-P 64–354, except for the size difference (Figs. 11, S5).

**Seals from the southern bank of the Kakhovka Reservoir (Zlatopil, Mayachka, Lysa Hora, Skelky), Ukraine** (“*Phoca novorossica*” sensu Gol’din et al., 2012), ZKM P-470, P-492, P 612, P-4950 (Table S2). Most of the seal bones found on the southern bank of the Kakhovka Reservoir are pachyosclerotic and similar (if not identical) to the specimens from Zhovtokamianka and Zolota Balka. The scapula ZKM P 612 has a straight cranial margin, whereas it is convex in *Paratethyphoca libera* MCFFM V-150. The proximal edge of the deltoid crest in *Paratethyphoca libera* MCFFM V-150 is slightly concave, and the deltoid crest is triangular. In seals from the Kakhovka Reservoir, two morphotypes were identified: the deltoid crest with a slightly concave/almost straight distal edge (ZKM-P-492 4; ZKM-P-492 17 (1–4); ZKM-P-470; ZKM-P-(1–4)), and rounded deltoid crest with a slightly convex distal edge (ZKM-P-4950(16)) (Fig. 10). The humerus of *Paratethyphoca libera* (MCFFM V-150) is longer (115 mm) than most of the humeri from the Kakhovka Reservoir, which vary from 83.0 to 91.0 mm, except for ZKM-P-492(4) which is also 115.0 mm. Also, the deltoid crest of *Paratethyphoca libera* MCFFM V-150 equals 50% of the bone length, the same as in ZKM-P-470; ZKM-P-(1–4) and only slightly different from ZKM-P-492(4) (55% of the bone length). Other phocids were also recorded from this locality, so its seal assemblage was relatively diverse (Gol’din et al., 2012).

***“Pachyphoca”*** **from Hulbocica, Lăpușna, Pruncul, Verejeni (Republic of Moldova)** (Obada, 2019), MNEIN FN 144/230b. The scapula MCFFM V-150 has a higher cranial margin compared to that of the Lăpușna specimen, in which it is straight and placed at the level of the glenoid (Fig. 7). The humerus MCFFM V-150 is larger than the humerus MNEIN FN 144/n/a (the length from the distal edge trochlea to the lesser tubercle is 86.0 and 67.0 mm, respectively (Fig. 10). The radii MCFFM V-150 and FN 144/230b are similar in shape, except MCFFM V-150 has the pronator teres process (Fig. 9). Both radii have a maximal width of 28% of the total bone length. In addition, the femur MCFFM V-150 exactly fits the outlines of the femur FN 54 294 from Pruncul and AICUPM MS-50 from Chișinău (Figs. 11, S5).

**A seal from Karagiye** (Kazakhstan, 8 in Fig. 2) (“*Pachyphoca*” sensu Lazarev et al., 2025), NU KDFs_23_1c. A fragmentary specimen coming from the Bessarabian debris at the surface of the section and consisting of coxae and a tarsal bone; it broadly resembles the specimens from the Republic of Moldova and Ukraine previously referred to this taxon.

### Commentary

Comparisons with unidentified isolated specimens and those previously described as *Pachyphoca* spp., *Phoca bessarabica,* or *Phoca novorossica* (the latter one is a *nomen nudum* lacking the holotype) are important, since they share an array of morphological traits and geological context coming mostly from the Upper Bessarabian deposits. We suggest most or even all of these specimens can be closely related to *Paratethyphoca libera* and represent at least the same genus. However, closer affinities of some of them to *Pontophoca sarmatica* and *Cryptophoca maeotica* cannot be ruled out, and the status and phylogenetic relationships of the latter taxon remain unclear.

### Comparison with other fossil taxa

***Devinophoca emryi*** (SNM Z27870–Z27874, SNM PP1502, PP1503)**.** This Paratethyan seal is known from the Upper Badenian (Serravallian, Middle Miocene) of the Central Paratethys (Koretsky & Holec, 2002; Koretsky & Rahmat, 2015; Rahmat & Koretsky, 2016). *Paratethyphoca libera* has a pronounced supraorbital process of the frontal, contrary to *Devinophoca emryi,* which has a reduced process. Its minimum interorbital width is located at the anterior half of the interorbital region (whereas on the posteriormost half of the interorbital region in *D. emryi*; Fig. S7) and is narrower (6% of the skull length vs 10% in *D. emryi*). *Paratethyphoca libera* has a maxillary swelling, which is missing in *D. emryi*. The humerus of *Paratethyphoca libera* has a concave distal transition of the deltoid crest (vs. straight in *D. emryi*). The largest height of the humeral diaphysis is located distally to the head in *Paratethyphoca libera,* whereas it is at the level of the distal edge of the humerus in *D. emryi*. The greater tubercle is lower than the head in *Paratethyphoca libera* (vs. higher in *D. emryi*). The supinator crest is lesser developed in *Paratethyphoca* than in *D. emryi* (Fig. S8).

***Histriophoca alekseevi*** (NMNHU-P 40–121). A Paratethyan seal known from the Bessarabian (“middle Sarmatian limestones”) of the Eastern Paratethys (Chișinău, Moldova) (Alekseev, 1924a; Koretsky, 2001). *Paratethyphoca libera* has a moderately wide nasal bone with a slight expansion in the rostral part. In contrast, based on the original description in Alekseev (1924a), *H. alekseevi* has barely noticeable and extremely narrow nasals expanding fan-like rostrally.

***Monachopsis pontica*** (FFM 10246, TNU CH00-01, humerus NMNHU-P 64–257). A Paratethyan dwarf seal from the Khersonian of the Eastern Paratethys (Kerch Peninsula, Ukraine) (Koretsky, 2001; Otriazhyi et al., 2025). *Paratethyphoca libera* has a pronounced supraorbital process of the frontal and a wider interorbital bridge (at least 7.5 mm wide), whereas *M. pontica* completely lacks the supraorbital process, and the interorbital bridge is narrow (4.5 mm wide). The nasal is wider in *Paratethyphoca libera* (8 mm in the medial section) than in *M. pontica* (2 mm wide). *Paratethyphoca libera* and *M. pontica* share a long snout: similar distances (~ 70 mm) from the nasal cavity to the cranium (Fig. S7). The scapula of *Paratethyphoca libera* has a more concave caudal margin than that in *M. pontica* (Fig. S9). *Paratethyphoca libera* has a humeral head slightly higher than the lesser tubercle, while *M. pontica* has a lesser tubercle higher than the head. The deltoid crest of *Paratethyphoca libera* is as long as half of the bone, while the deltoid crest in *M. pontica* is longer than 70% of the total bone length. *Paratethyphoca libera* has a smooth transition of the deltoid crest in the distal end, whereas this transition in *M. pontica* is sharp. The humeral lateral epicondyle of *Paratethyphoca libera* is shorter than that in *M. pontica* (28% vs. 38% of the bone total length) (Fig. S8).

***Praepusa tarchankutica*** (NMNHU-P 64–468, 64–469). A Paratethyan seal from the Bessarabian deposits of the Eastern Paratethys (Ukraine) (Antonyuk & Koretsky, 1984; Koretsky, 2001). *Paratethyphoca libera* has a pronounced supraorbital process of the frontal, absent in *Pr. tarchankutica*. *Paratethyphoca libera* has a wider interorbital bridge, at least 7.5 mm wide, it measures 3.5 mm whereas in the skull NMNHU-P 64–469 *Pr. tarchankutica* (this skull is larger than that of *Paratethyphoca*, see Fig. S7). The chin prominence is well-developed in *Paratethyphoca*, whereas it is moderately developed in *Pr. tarchankutica*. The lesser tubercle of *Paratethyphoca libera* is lower than the head of the humerus. Its deltoid crest is about half of the bone length, while *Pr. tarchankutica* has a lesser tubercle higher than the head, and the deltoid crest is 65% of the bone length. The distal termination of the humeral deltoid crest is smooth in *Paratethyphoca* and sharp in *Pr. tarchankutica. Paratethyphoca libera* has a short lateral epicondyle (28% of bone length), whereas it is 38% and well-developed caudally in *Pr. tarchankutica* (Fig. S8).

***Planopusa semenovi*** (NMNHU-P 64–709). A Paratethyan seal known from the Bessarabian deposits of the Eastern Paratethys (Hrytsiv, Ukraine) (Koretsky & Rahmat, 2021). *Pl. semenovi* has been described based on the anterior parts of the premaxilla and maxilla. This specimen does not overlap to a great extent with the skull of *Paratethyphoca.* In addition, the precise measurement of the anterior part of *Paratethyphoca libera* is not possible due to its slight lateral compression. However, as preserved, *Paratethyphoca libera* does not show a widening of the rostrum and seems to have a long and gracile snout, whereas *Pl. semenovi* is distinguished by an especially wide and short anterior portion of the rostrum.

***Sarmatonectes sintsovi ***Koretsky, 2001. The holotype of *Sarmatonectes sintsovi* is an isolated femur PIN 1713/140, in which the diaphysis is narrower than that in MCFFM V-150 (22.0 vs. 28.0 mm). In addition, the humerus PIN 1713/146 was assigned to *Sarmatonectes sintsovi* (Koretsky, 2001). The humerus of MCFFM V-150 differs from PIN 1713/146 in a shorter deltoid crest (~ 50% of the bone) and a greater tubercle at the level of the humeral head. In contrast, the deltoid crest in PIN 1713/146 is 65% of the bone length, and its greater tubercle is higher than the humeral head. Based on these differences, we assume these materials belong to representatives of different taxa. Also, until a more complete specimen is found, we agree with the suggestion to consider *Sarmatonectes sintsovi* as *nomen dubium* (Rule et al., 2024).

### Comparison with living taxa

*Paratethyphoca libera* differs from all extant true seals (Phocidae), except for *Cystophora cristata*, in having the supraorbital process of the frontal. The traits shared only by extinct phocines (Dewaele et al., 2022) include a thick and pachyosclerotic scapula, humerus, and femur. The humerus has a smooth transition of the distal end of the deltoid crest to the diaphysis, while it is sharp in most extant phocids, except for some individuals of *Histriophoca fasciata*. The lateral edge of the femoral diaphysis is almost straight, while it is concave in modern phocids (Fig. S8).

***Erignathus barbatus*** (NMW CN958, CN958, 7556). The cranial edge of the nasal of *Paratethyphoca libera* is pointed (vs. rounded in *E. barbatus*). The nasal bone of *Paratethyphoca libera* is narrower: its medial width reaches 10% and the maximum width equals 16% of the total bone length, while in *E. barbatus* they are 18% and 22%, respectively (Fig. S7). The supraspinous fossa of the scapula has smaller maximal height than the height of glenoid fossa in *Paratethyphoca*, while *E. barbatus,* has the maximal height of the supraspinous fossa larger than the height of the glenoid fossa (Fig. S9). *Paratethyphoca libera* has a lesser tubercle lower than the proximal edge of humeral head and a greater tubercle at the same level as the head’s proximal edge, while both tubercles in *E. barbatus* are placed higher than the head. The trochideltoid surface is small in *Paratethyphoca*, while it is well-developed and wide in *E. barbatus* (Fig. S8). *Paratethyphoca libera* has the insertion of the *m. pronator teres* of the radius proximally to the middle of the bone, while it is located distally in *E. barbatus*. The insertion of the *m. brachioradialis* is 24% of the bone length in *Paratethyphoca libera* (vs. 38% in *E. barbatus*).

***Cystophora cristata*** (ZMUC 1265, 1134). *Paratethyphoca libera* has a narrow nasal bone with a pointed cranial edge. The medial width equals 10%, and the maximum width—16% of the bone total length. In contrast, the nasal of *C. cristata* is short and wide, with a rounded cranial edge (Fig. S7): its medial width varies from 11% to 17%, and the maximal width varies from 27% to 33% of the nasal total length). Alveoli of pm2 are well separated in *Paratethyphoca libera* but fused in *C. cristata*. The maxillary part of the snout is long in *Paratethyphoca libera* (23% of the nasal cavity-occipital length), whereas it is short in *C. cristata* (7% of the nasal cavity-occipital base length). The premaxilla-nasal suture is present in *Paratethyphoca libera* and absent in *C. cristata*. The mandible of *Paratethyphoca libera* has a well-developed chin prominence with a concave ventral edge, whereas in *C. cristata* the chin prominence is absent, and the ventral edge is slightly convex. The supraspinous fossa of the scapula is low in *Paratethyphoca libera* (its maximal height is smaller than the height of the glenoid fossa), and the cranial margin is slightly convex. In contrast, this fossa in *C. cristata* is high (its maximal heigth is up to twice as larger as the height of the glenoid fossa), and the cranial margin is convex. *Paratethyphoca libera* has a lesser tubercle lower than the head, whereas in *C. cristata* it extends above the head (Fig. S8).

### Histriophocini

***Pagophilus groenlandicus*** (Skull NMBE 638, ZMUC 154, ZMUC CN 961). The orbital-nasal distance of *Paratethyphoca libera* is longer than that in *Pa. groenlandicus,* making 25% of occipital-nasal cavity length in *Paratethyphoca libera* and 15% in *Pa. groenlandicus* (Fig. S7). The mandible has a well-developed chin prominence in *Paratethyphoca libera*, and it is slightly developed in *Pa. groenlandicus*. The supraspinous fossa of the scapula is low (its maximum height is smaller than the height of the glenoid fossa) in *Paratethyphoca*, whereas it is height in *Pa. groenlandicus* (its maximum height is 1.5 times larger than the height of the glenoid fossa). The lesser tubercle of the humerus is lower than the head in *Paratethyphoca libera* and higher in *Pa. groenlandicus*. The highest point of the lateral epicondyle is located around the midpoint of the epicondyle in *Paratethyphoca*, while in *Pa. groenlandicus* it is located proximally. The trochlea is narrower than the humeral head in *Paratethyphoca libera* and equally wide to the humeral head in *Pa. groenlandicus* (Fig. S8). The radius of *Paratethyphoca libera* is narrow (maximal width 28% of the bone total length), whereas it is wide in *Pa. groenlandicus,* 30% of the bone total length.

***Histriophoca fasciata*** (ZMUC CS-303-67). The maxillary portion of the snout is longer (25% of the skull occipital-nasal cavity length) in *Paratethyphoca libera*, whereas it is 16% in *H. fasciata*. *Paratethyphoca libera* has a supraspinous fossa of the scapula low (its maximum height is smaller than the height of the glenoid fossa vs. the height of the fossa is the same or 1.5 times larger than the height of the glenoid fossa in *H. fasciata* (Fig. S7). The humerus of *Paratethyphoca libera* has a lesser tubercle lower than the humeral head, and the width of the trochlea is 65% of the total humeral head width. In contrast, the lesser tubercle in *H. fasciata* is higher than the head, and the trochlea is about the same width as the humeral head. The intertubercle groove is narrow in *Paratethyphoca libera* and wide in *H. fasciata*.

### Phocini

#### *Phoca vitulina* (NMW 1462, 28587)

The nasals are narrow in *Paratethyphoca libera* (the medial width is 10% of the bone length). In contrast, the nasals in *Ph. vitulina* are wide (20%–23% of bone length). The chin prominence of the mandible is well-developed in *Paratethyphoca* (vs. poorly developed in *Ph. vitulina*; Fig. S7). The supraspinous fossa of the scapula is shallow (its maximum height is smaller than the height of the glenoid fossa) in *Paratethyphoca* and large in *Ph. vitulina* (its maximum height is larger than the height of the glenoid fossa, Fig. S9). The humerus of *Paratethyphoca libera* has lesser tubercles below the level of the head, the proximal bifurcation of the deltoid crest is absent, and the lateral epicondyle slightly protrudes caudally. In contrast, the lesser tubercle in *Ph. vitulina* is above the level of the head, there is a proximal bifurcation in the deltoid crest, and the lateral epicondyle strongly protrudes caudally (Fig. S8).

#### *Halichoerus grypus* (ZMUC 1485, NMW 28539)

From the lateral view, the snout of *Paratethyphoca libera* becomes narrower rostrally. The rostral edge of the premaxilla is concave. In contrast, the nasal is almost parallel to the tooth row, and the rostral edge of the premaxilla is straight in *Ha. grypus*. The nasal bone is narrower in *Paratethyphoca libera* (the medial width is 10% and the maximal width is 16% of the bone total length), while in *Ha. grypus,* they are equal to 15% and 24%, respectively (Fig. S7). The chin prominence is well-developed in *Paratethyphoca libera* and slightly developed in *Ha. grypus*. The maximal height of the supraspinous fossa is smaller than the height of the glenoid fossa in *Paratethyphoca*; contrary to *Ha. grypus*, the height of the supraspinous fossa is larger than the height of the glenoid fossa. Two ridges on the lateral side do not join near the glenoid in *Paratethyphoca* (vs. being joined in *Ha. grypus*; Fig. S9). *Paratethyphoca libera* has the lesser tubercle of the humerus below the level of the head, and the proximal bifurcation of the deltoid crest is absent, while in *Ha. grypus* the lesser tubercle is higher than the head, and the proximal bifurcation in the deltoid crest is present (Fig. S8). The radius of *Paratethyphoca libera* is narrower in lateral view (28% of the bone length in *Paratethyphoca*, and 35% in *Ha. grypus*).

***Pusa hispida*** (ZMUC 803). The rostral length of *Paratethyphoca libera* (from the nasal cavity to the orbit) is 25% of the occipital-nasal cavity length of the skull, while in *Pu. hispida* it makes 20%. *Paratethyphoca libera* has a larger minimal width of the interorbital bridge: 6% of the skull nasal cavity-occipital base length in *Paratethyphoca*; and 3% in *Pu. hispida*. The chin prominence is well-developed in *Paratethyphoca libera* and weakly developed in *Pu. hispida*. *Paratethyphoca libera* has a supraspinous fossa whose maximal height is shallower than the height of the glenoid fossa (vs. higher in *Pu. hispida*). Two lateral ridges do not join near the glenoid in *Paratethyphoca libera*, and they are joined in *Pu. hispida*. The humeral lesser tubercle is below the level of the head in *Paratethyphoca libera* and higher in *Pu. hispida*. The proximal bifurcation of the deltoid crest is absent in *Paratethyphoca libera* and present in *Pu. hispida*. The lateral epicondyle is weakly developed caudally in *Paratethyphoca libera,* and strongly developed in *Pu. hispida*. The radius of *Paratethyphoca libera* is thinner from the lateral view—its maximum width is 28% of the bone’s total length, while it equals 32% in *Pu. hispida*.

***Pusa caspica*** (NMW 66292–66299, GNM 2–2013/988). The nasal cavity-orbital length of the rostrum is longer in *Paratethyphoca libera*, making up 25% of the nasal cavity-base of occipital length in *Paratethyphoca libera* and 20% in *Pu. caspica* (Fig. S7). The mandible of *Paratethyphoca libera* has a well-developed chin prominence, and its ventral edge is strongly concave, while *Pu. caspica* has a moderately developed chin prominence, and the ventral edge of the mandible is slightly concave. The scapula of *Paratethyphoca libera* has a low supraspinous fossa (its maximum height is smaller than the height of the glenoid fossa), and lateral ridges are not joint near the glenoid, whereas in *Pu. caspica,* the maximum height of the supraspinous fossa is larger than the height of the glenoid fossa and the lateral ridges join together near the glenoid (Fig. S9). The humerus of *Paratethyphoca libera* has a lesser tubercle below the level of the head. The proximal bifurcation of the deltoid crest is absent, and the lateral epicondyle is weakly developed in the caudal direction. In contrast, *Pu. caspica* has a lesser tubercle higher than the head, a well-developed proximal bifurcation of the deltoid crest, which in some individuals even joins with the lesser tubercle and the lateral epicondyle is well developed in a caudal direction (Fig. S8). *Paratethyphoca libera* has a thin radius in lateral view; it makes only 28% of the bone length vs. 35% in *Pu. caspica*.

### Phylogeny

**Fig. 12:**
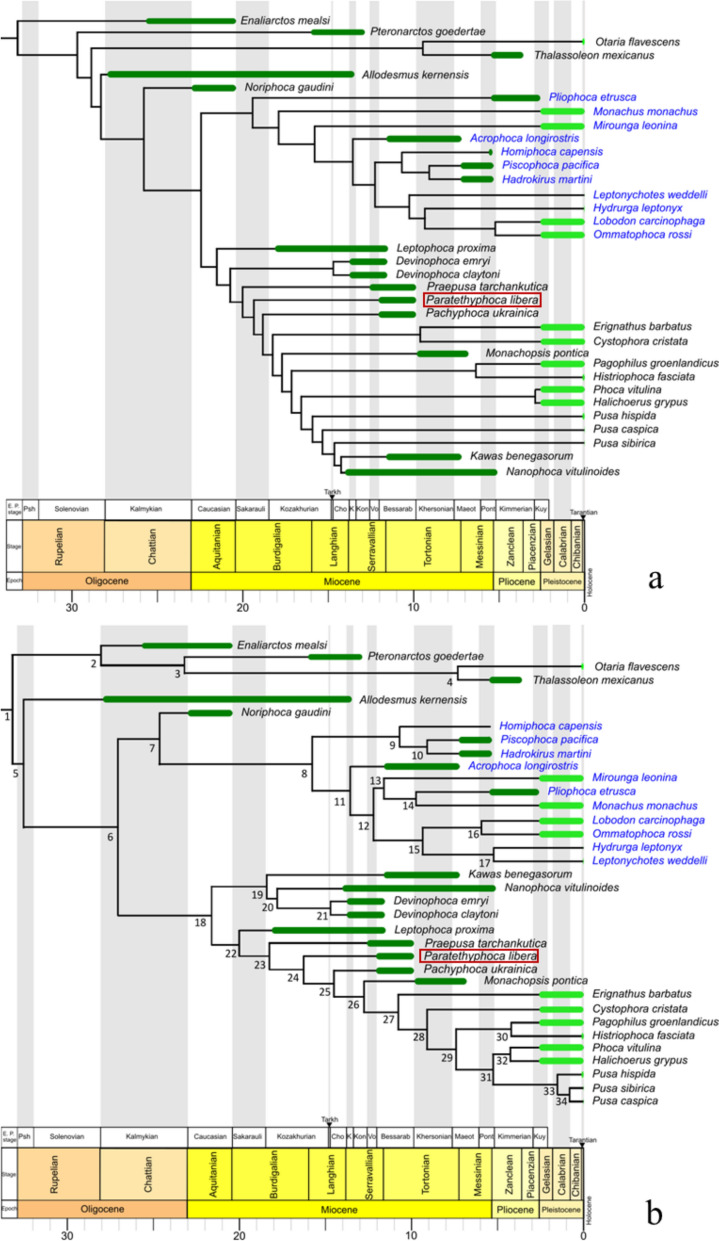
Phylogenetic trees of true seals. **a** Parsimony tree with Implied weighting (k = 3), consistency index 0.352, retention index 0.705; **b** Total evidence tree, age of nodes and 95% highest probability density see in the Table S8. Abbreviations for the regional (sub)stages: Sakarauli—Sakaraulian; Tarkh—Tarkhanian; Cho—Chokrakian; K—Karaganian; Kon—Konkian; Vo—Volhynian; Kherson—Khersonian; Maeot—Maeotian; Pont—Pontian; Kuy—Kuyalnikian

The parsimony tree with implied weighting places *Paratethyphoca libera* in the stem Phocinae, crownwards to most other fossil Phocidae except *Pachyphoca ukrainica, Monachopsis pontica, Kawas benegasorum*, and *Nanophoca vitulinoides* (Fig. 12a)*.* In the total evidence analysis, for most statistics, Effective Sample Size is more than 200. The exceptions (Effective Sample Size more than 100 for the second run) were in the: Diversification rate fossilised birth–death and turn over fossilised birth–death in both runs and prior, tree height, tree length, uncorrelated lognormal relaxed clock mean for morphological partition, and the rate for morphological partition. *Paratethyphoca libera* formed the clade with modern Phocinae and some Paratethyan taxa (*Pp. ukrainica* and *M. pontica*) (Fig. 12b). Based on the analysis, *Paratethyphoca libera* diversified from other Phocinae between 14.57 and 17.46 Ma (95% highest probability density: Table S10). In both trees, it is placed crownwards to *Praepusa tarchankutica.*

### Generalized procrustes analysis

Two analyses were made for both humerus and femur. The first analysis included all available Phocinae, with Paratethyan taxa (*“Pachyphoca”* sp., *Praepusa* sp., *Cryptophoca maeotica*, *Pontophoca sarmatica*, *Monachopsis pontica*). Also, another round of analyses excluded Phocini from the dataset: Phocini have a well-developed proximal bifurcation of the deltoid crest of the humerus—in contrast to other Phocinae (including Paratethyan taxa, which are in the focus of this article), which do not have such a strongly developed bifurcation; therefore, Phocini were found as outliers in the pooled dataset.

In the Generalized Procrustes Analysis of the humerus with Phocini, the PC1 described 25.9% of variation, the PC2—12%; without Phocini the PC1 described 19.1% of variation, the PC2—16.4%. In the Generalized Procrustes Analysis of the femur with Phocini, the PC1 described 24.4% of variation, the PC2—19.5%; without Phocini, the PC1 described 23.2% of variation, and the PC2—19.7%.

**Fig. 13:**
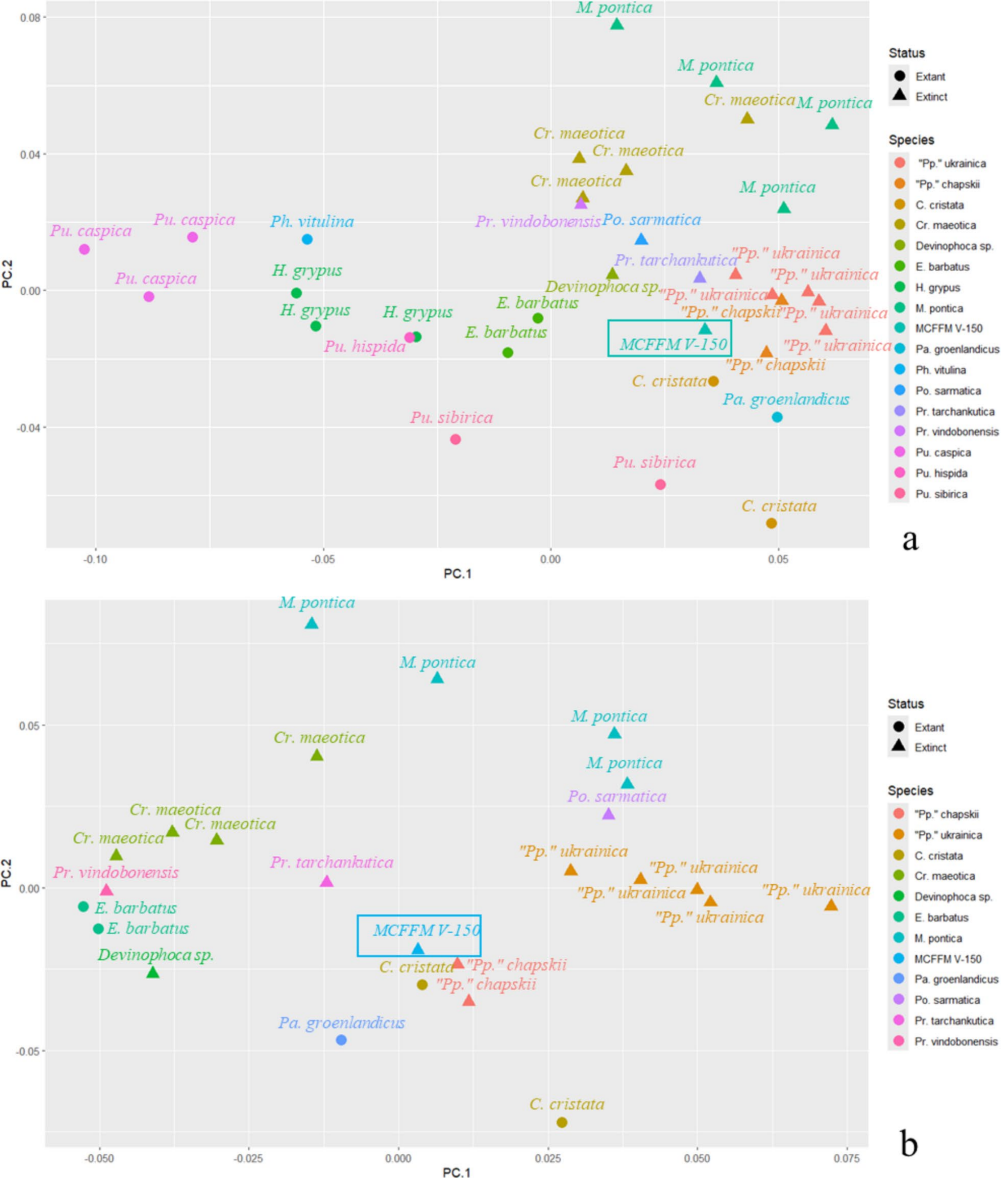
Principal components (PC1 and PC2) of the Generalized Procrustes Analysis of the humerus. With Phocini included in (**a**) and excluded (**b**) from the dataset. The morphological variation along PC1 and PC2 is illustrated in Fig. S11

In the analysis of humeri with Phocini, the latter mostly occupied the negative side of PC1, while the other Phocinae (except *Erignathus barbatus*) were on the positive side of PC1. *Praepusa vindobonensis* NWM SK175, *Cryptophoca maeotica, Monachopsis pontica* were on the top right quarter of the plot, while *Devinophoca emryi*, *“Pachyphoca”* sp.*, Paratethyphoca libera* MCFFM V-150*,* female *Cystophora cristata* and *Pagophilus groenlandicus* formed a cluster on the right*-*middle side of the plot. *Pontophoca sarmatica* NMNHU-P 1713-10 was situated between these groups (Fig. 13a). On PC1, the morphological variation is mostly observed in the shape of the distal extension of the deltoid crest and development of its proximal bifurcation. In negative coordinates of PC1, the distal extension of the deltoid crest is sharp, its proximal bifurcation is well developed, and the medial epicondyle is at the same level as the lateral one. In contrast, in positive coordinates, the distal extension of the deltoid crest is smooth, its proximal bifurcation is absent, and the medial epicondyle is lifted proximally compared to the lateral one. On PC2, the morphological variation is mostly seen in the length of the deltoid crest. In negative coordinates, the crest is short (about half of the bone), whereas in positive coordinates it is long (up to 70% of the bone length), and the humerus is mediolaterally compressed. In both PC1 and PC2 in negative coordinates, the lesser tubercle is large, and it is higher than the proximal edge of the head, whereas in positive coordinates, it is small and situated lower than the head (Fig. S11).

In the analysis of humeri without Phocini, *Erignathus barbatus, Cryptophoca maeotica* (except NMNHU-P 64–530)*, Praepusa vindobonensis* NWM SK175, and *Devinophoca emryi* form a group on the negative half of the PC1. *Paratethyphoca libera* MCFFM V-150, *“Pp.” chapskii* and female *C. cristata* pooled together near the centre. *M. pontica* and *Cr. maeotica* NMNHU-P 64–530 occupied the positive half of the PC2. *“Pp.” ukrainica* was near the 0 value of the PC2, and on the positive half of the PC1, while *Po. sarmatica* NMNHU-P 1713-10 was between *M. pontica* and *“Pp.” ukrainica* (Fig. 13b)*.* On PC1, the morphological variation is mostly observed in the general humerus shape and position of the medial epicondyle. In negative coordinates of PC1, the humerus is gracile: the diaphysis and epiphysis are narrow, the humeral head is small, and the medial epicondyle is located slightly distally than the lateral one. In positive coordinates, the bone is more robust: the humeral head is large, and the diaphysis and epiphysis are wide; the medial epicondyle is located proximally to the lateral one. On PC2, the morphological variation is mostly seen in the length of the deltoid crest: in negative coordinates, it is as short as half of the bone, and in positive coordinates, it is longer than half of the bone, reaching the medial epicondyle. Also, in negative coordinates, the humeral head is proximally directed, and in positive coordinates it is more distally projected (Fig. S11).

**Fig. 14:**
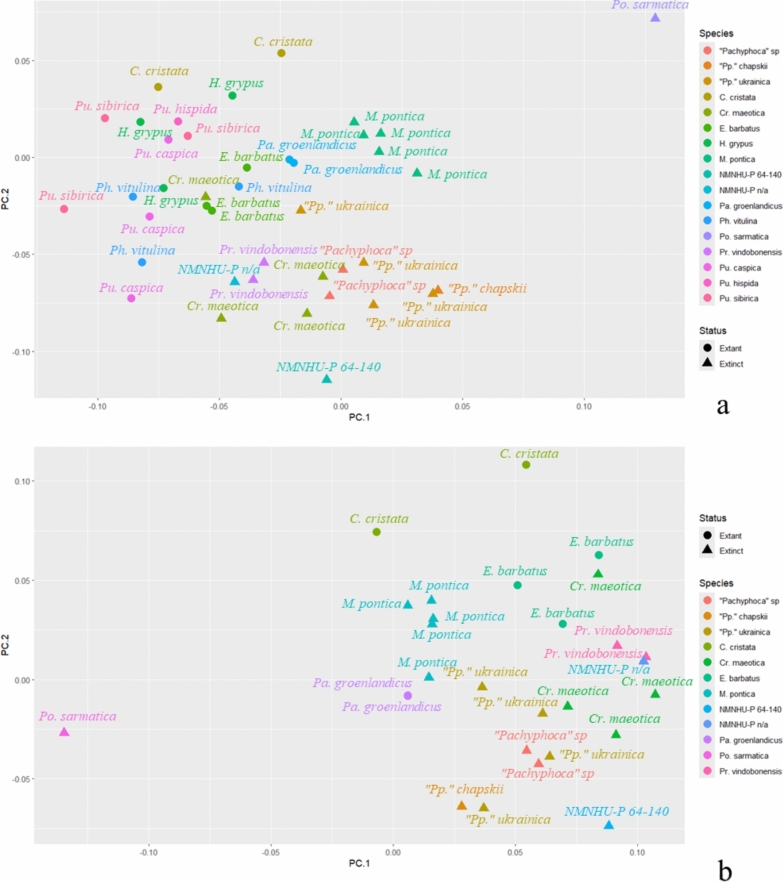
Principal components (PC1 and PC2) of the Generalised Procrustes Analysis of the femur. With Phocini included in (**a**) and excluded (**b**) from the dataset. The morphological variation along PC1 and PC2 is illustrated in Fig. S12

In the analysis of femur with Phocini, datapoints of the modern species formed a group with no distinguishable groups inside. *Pontophoca sarmatica* was separated from the other Phocinae. *Monachopsis pontica* made a compact group near the 0 value of the PC2 and a slightly positive half of the PC1. Another large cluster was located in the negative half of the PC1, near the 0 value of the PC2 and included *Praepusa vindobonensis*, *Cryptophoca maeotica* and “*Pachyphoca*” spp. (Fig. 14a). On PC1, the morphological variation is mostly observed in the sizes of the greater trochanter and medial epicondyle, and in the shape of the lateral edge of the diaphysis. In negative coordinates of PC1, the femur has a large greater trochanter, a short medial epicondyle and a strongly concave lateral edge of the diaphysis. In positive coordinates, the greater trochanter is small, the medial epicondyle is long (reaching the proximal half of the bone), and the lateral edge of the diaphysis is slightly concave. On PC2, the femur varies in general shape: the negative coordinates represent an elongated femur with a narrow distal epiphysis, the greater trochanter and the femoral head are small, and the condyles are similar in size. In positive coordinates, the femur is short and wide with a mediolaterally wide distal epiphysis, a robust greater trochanter, and the lateral condyle is larger than the medial one (Fig. S12).

In the analysis of femur without Phocini, *Pontophoca sarmatica* was located separately from other Phocinae in the negative part of the PC2, and *“Pachyphoca”* spp. and *Cryptophoca maeotica* were on the positive half of the PC1 and negative half of the PC2. *Monachopsis pontica* was situated separately from *Praepusa vindobonensis, Erignathus barbatus,* and *Cystophora cristata* at the positive end of the PC1 (Fig. 14b). In negative coordinates of PC1, the femur is wide with a well-developed medial epicondyle, the femoral neck is long, and the greater trochanter is at the same level as the head. In the positive coordinates, the femur is narrow, its medial epicondyle smoothly connects to the bone diaphysis, the femoral neck is short, and the greater trochanter is higher than the head. On PC2, the femur varies in proportions of the greater trochanter and medial epicondyle and the shape of the lateral edge of the diaphysis. In the negative coordinates of PC2, the femur has a small greater trochanter, a large medial epicondyle (as long as the half of the bone), and the lateral edge of the diaphysis is slightly concave, whereas in positive coordinates the greater trochanter is large, the medial epicondyle is short, and the distal edge of the diaphysis is strongly concave (Fig. S12).

## Discussion

### Phylogenetic analysis

The maximum parsimony and total evidence analyses placed *Paratethyphoca libera* among other Paratethyan taxa, except for *Monachopsis pontica* in parsimony analysis (in which *M. pontica* is placed among the crown group) (Fig. 12a) and *Devinophoca* sp. in total evidence analysis (in which it formed a separate clade with *Nanophoca vitulinoides* and *Kawas benegasorum*) (Fig. 12b). These unresolved topographies could be caused by the lack of material (especially the cranial parts) for some Miocene taxa and the lack of data overlap between some of the species (e.g., *Kawas benegasorum* and *Leptophoca proxima* do not overlap with *Devinophoca claytoni*). However, they do not alter the position of *Paratethyphoca libera* on the phylogenetic tree. In our analysis, *Paratethyphoca libera* and *Pachyphoca ukrainica* do not form a clade with *C. cristata,* unlike in Koretsky and Rahmat (2013). However, adding characteristics of the ilium NMNHU-P OF 1207 from Zhovtokamianka to *Paratethyphoca libera* (this specimen was previously assigned to *Pachyphoca chapskii* (Koretsky & Rahmat, 2013)) changed the position of *Paratethyphoca libera* and *Pp. ukrainica* in such a way that both taxa formed clades with *Cystophora cristata*, as shown in the supplementary tree (Fig. S13). Therefore, their close relatedness to each other and to *Cystophora cristata*, as first suggested by Koretsky and Rahmat (2013), cannot be ruled out. Future findings of the coxae of *Paratethyphoca libera* may be crucial for revealing the evolutionary connection between modern and Paratethyan seals.

### Generalized procrustes analysis (GPA)

The GPA does not show a clear separation between Phocini; however, it was successful in separating Paratethyan seals from other members of the Phocidae. In humeral morphology, Paratethyan taxa are distinguished in the smoother distal end of the deltoid crest than most of the living seals, and most of them have a longer deltoid crest (e.g., *Monachopsis pontica, Cryptophoca maeotica, Praepusa tarchankutica),* and a lesser tubercle lower than the proximal edge of the humeral head*.* In femoral morphology, Paratethyan seals differ from most of the living taxa by a longer medial epicondyle and only a slightly concave lateral edge of the diaphysis. However, there is a very high morphological variation among Paratethyan taxa. Among them, there are those with a short deltoid crest of the humerus (*Paratethyphoca libera*, *Devinophoca sp.*), but there are also species with an extremely long deltoid crest (e.g., *Monachopsis pontica).* In the case of the femur, there are specimens with a short medial epicondyle (like *Monachopsis pontica* NMNHU-P 64–314), and there are taxa with very long (*Paratethyphoca libera*) or extremely wide medial epicondyle (*Pontophoca sarmatica*). The small sample size and uncertain phylogenetic relationships of some species, like *Cryptophoca maeotica* or *Praepusa tarchankutica*, could cause the unresolved separation of some Paratethyan taxa. Nevertheless, *Paratethyphoca libera* was the closest to the specimens that have been referred to as *Pachyphoca chapskii* in previous studies (Koretsky & Rahmat, 2013) and distinguished from other Paratethyan seals.

### Taxonomy

MCFFM V-150 differs from most Paratethyan seals in a few significant characteristics: a relatively short deltoid crest, its smooth distal transition, frontal with supraorbital process, and other differences discussed above. On the other hand, MCFFM V-150 has similarities with fossils assigned to *Pachyphoca* and *Pontophoca.* However, both genera are represented only by species with only isolated bones designated as holotypes.

The holotype for *Pachyphoca ukrainica* is a humerus NMNHU-P 64–701 from Khomutove, with no paratype. The holotype for *Pachyphoca chapskii* is an incomplete femur NMNHU-P 64–706; also, some other bones (including humerus), all originated from the Zhovtokamianka locality, have been assigned to this species with no special evidence, mostly due to their relatively large size (Koretsky & Rahmat, 2013). However, the humerus of *Pp. ukrainica* has also been recorded from Zhovtokamianka. In addition, a specimen similar to *Pp. chapskii* (sensu Koretsky & Rahmat, 2013) was also found among fossils similar to *Pp. ukrainica* at the southern bank of the Kakhovka Reservoir. Since the most obvious difference between the two nominal *Pachyphoca* species is their size, the other differences may result from the within-species variation. In addition, both taxa were considered as *nomina dubia* in recent publications (Rule et al., 2024; Valenzuela-Toro & Pyenson, 2019) due to their fragmentary holotypes, and our data corroborate this assumption. In the context of this study, we cannot assign MCFFM V-150 to *Pachyphoca ukrainica* due to morphological differences in their humeral anatomy. Also, we cannot assign it to *Pp. chapskii,* due to the incompleteness of both the holotype of *Pp. chapskii* and the femur MCFFM V-150. Therefore, we can only suspect a possible close relationship among them and cannot synonymize any of them; also, at present, referring to the nominal *Pachyphoca* species should be limited to their holotypes. The same fully applies to *Phoca bessarabica*, the first described morphologically similar species.

The lectotype of *Pontophoca sarmatica* is an isolated femur with a distinct autapomorphic shape and large epicondyles (Alekseev, 1924b; Koretsky & Grigorescu, 2002). The only preserved part of the femur of MCFFM V-150 is the diaphysis. Still, even this is enough to see the difference between the two femora, since *Pontophoca sarmatica* has strongly concave lateral edges of the femur. In contrast, MCFFM V-150 has only slightly curved ones. Considering this argumentation, we cannot assign MCFFM V-150 to *Pontophoca sarmatica.* However, there are bones assigned to *Pontophoca sarmatica* that share many similarities with MCFFM V-150, such as a humerus NMNHU-P 1713/10 and a scapula from the original description provided by Alekseev (1924a, 1924b). To study this in depth, articulated skeletons of *Pontophoca sarmatica* are needed.

Therefore, here we introduce the specimen MCFFM V-150 as representing a new taxon *Paratethyphoca libera*. Based on the geometric morphometry of the humerus and femur, phylogenetic analysis and overall morphological similarities between MCFFM V-150 and several specimens previously identified as *Pachyphoca ukrainica* and *Pp. chapskii*, we suggest for tentative identification of many materials previously identified as *Pachyphoca*, to the same genus *Paratethyphoca* with a question mark, including the specimens from Zhovtokamianka, Zolota Balka, Khomutove, Lăpuşna, and the Kakhovka Reservoir. We admit the necessity of having more complete specimens from each of these localities for firm conclusions on their taxonomy.

### Tooth wear

The teeth of *Paratethyphoca libera* MCFFM V-150 have almost completely worn crowns. Similar tooth wear also observed in other Paratethyan seals, in particular in *Monachopsis pontica* (Otriazhyi et al., 2025). Among modern Phocidae, such a strong wear sometimes develops in *Erignathus barbatus* due to its glosswear suction feeding (Marx et al., 2023). Tongue motions during such feeding lead to the wearing of the canines, and since canines lose their function as occlusal guides (Mellett, 1985), postcanine teeth get worn. Therefore, it is reasonable to assume that *Paratethyphoca libera* might primarily employ suction feeding as a main strategy for capturing prey.

Considering that *Paratethyphoca*, *Monachopsis pontica* and *Erignathus barbatus* are stem taxa as for other modern Phocinae, we hypothesise that suction prey capture could be widespread among early Phocinae as an important feeding strategy and lost in most of crown groups. To test this hypothesis properly, we need further findings on the dentition of Paratethyan seals.

### Diversity and disparity in anatomy

A high diversity in humeral and femoral morphotypes is characteristic for the Bessarabian seals. It may reflect both taxonomical richness and intraspecies variation. Different morphological forms may represent different taxa of species- or genus-level, but some of them may also represent chronological variation, ecotypes, individual variation or sex and age groups. Among the humeral morphotypes, gracile humeri with a long deltoid crest are assigned to *Cryptophoca* and *Praepusa*; whereas pachyosteosclerotic humeri with a short deltoid crest are typical for *Paratethyphoca* and morphologically similar forms. Morphotypes of femora are represented by gracile bones associated with *Cryptophoca* and *Praepusa,* whereas pachyosteosclerotic femora are characteristic to *Paratethyphoca*; and the femora with large epicondyles to *Pontophoca sarmatica*. In addition, disparity between humeral and femoral morphologies can be observed across these diverse forms: similarity or even sameness in humeral morphology may co-occur with strong differences in femoral morphology and vice versa, so that distributions of forms in the humeral and femoral morphospaces do not correspond to each other. Additionally, the diagnostic value of differences in postcranial morphology is sometimes questionable, as found in previous studies (Churchill & Uhen, 2019; Rule et al., 2024). However, this value varies from case to case. For example, the length of the humeral deltoid crest may be a useful character to distinguish at least some genera, whereas the importance of the length and proximal development of greater and lesser tubercles is uncertain. For example, the humeri of MCFFM V-150 and the specimens from Zolota Balka are very similar, although they differ in the shape of their cranial margins. MCFFM V-150 has a higher and more convex cranial margin, and seals from Zolota Balka have a lower and straighter margin. Thus, a wide screening of such features and the study of their variation in living and extinct species is needed to discover which characters (if any) can be used in taxonomic revisions of Phocidae.

Nevertheless, obvious morphological diversity, even if due to individual variation, is likely evidence for geographical partition, ecological diversification, or the effect of both these processes. Moreover, in addition to seals, other groups such as cetaceans (Gol'din & Startsev, 2017) and teleost fishes (Bratishko et al., 2017, 2023) demonstrate high diversity in the Eastern Paratethys during the Bessarabian. The marine fish fauna was quite rich, and several endemic speciation events were detected during that time (Bratishko et al., 2023). High diversification in fish assemblages could have been a background for the subsequent ecological niche partitioning among the seals. For example, a highly variable femoral morphology suggests different swimming styles and, as a result, different hunting strategies of seals. To test this assumption, complete skeletons of associated hindlimb bones are needed for comparison. A high variation in tooth morphology observed in the Bessarabian seals also supports this assumption, since the tooth size and shape of their crowns can help to comprehend the feeding strategy of seals (Churchill & Clementz, 2015; Ishihara et al., 2024). Among the Bessarabian seals, there are forms with well-developed accessory cusps, like *Praepusa tarchankutica* (Antonyuk & Koretsky, 1984), large main cusps and small accessory cusps, like *Planopusa semenovi (*Koretsky & Rahmat, 2021), and heavily worn tooth crowns, like *Paratethyphoca libera* described herein*.* These features indicate different feeding and prey capture strategies and diet specialization. In conclusion, the remarkable morphological diversity of sympatrically living Paratethyan seals can be partly explained by the feeding diversification, backed by speciation in their prey, and occurred in parallel to speciation in other top predators represented by marine mammals.

## Conclusions

The newly described seal MCFFM V-150 showed autapomorphic cranial features. Although its postcranial elements showed some similarities with previously described Paratethyan seals, the MCFFM V-150 is different from the other taxa. Several previously described nominal taxa have been suggested as *nomina dubia* due to the fragmentary, poorly diagnostic nature of their type material. Generalized Procrustes analysis of geometric morphometry of the humerus and femur do not show a clear separation of some Paratethyan seals, previously referred to as several genera, and suggested grouping these taxa for their potential revision, to be based on articulated skeletons. Here we formally described MCFFM V-150 as a new genus and species *Paratethyphoca libera* and suggested a tentative assignment of some previously known fossils from several localities of the same age to this genus. Phylogenetic analysis showed the stem position of *Paratethyphoca libera* within the subfamily Phocinae; however, its close relationship to a living hooded seal *Cystophora cristata,* cannot be ruled out. The tooth wear of *Paratethyphoca libera* may indicate a suction prey capture strategy shared by a Paratethyan seal *Monachopsis pontica,* and a living bearded seal *Erignathus barbatus.* The overall morphological diversity of the Paratethyan seals of the Bessarabian, even if due not only to speciation but also to individual variation, is evidence for their geographical partition, ecological diversification, or both processes, which were backed by speciation in their prey.

## Supplementary Information

Additional file 1

Additional file 2

Additional file 3

Additional file 4

Additional file 5

Additional file 6

Additional file 7

Additional file 8

Additional file 9

Additional file 10

Additional file 11

Additional file 12

Additional file 13

Additional file 14

Additional file 15

Additional file 16

## Data Availability

The data used and analysed during the current study are included in this published article and its supplementary information files or are available from the corresponding author on reasonable request.

## Abbreviations

AICUPM: Alexandru Ioan Cuza University Department of Geology Collections, Iași, Romania
FFE: Feldman Family Museum, Kharkiv, Ukraine
GNM: Georgian National Museum, Tbilisi, Georgia
HMZ: Museum of Zoology, Helsinki, Finland
IZ NASU (before 1998 known as IZAN): Schmalhausen Institute of Zoology, National Academy of Sciences of Ukraine, Kyiv, Ukraine
MCFFM: Muzeul Complexelor faunistice fosile din Moldova (Museum of Fossil Faunistic Complexes of Moldova), Chișinău, Moldova
MNEIN: National Museum of Ethnography and Natural History, Chișinău, Moldova
NMB: Natural History Museum Basel, Basel, Switzerland
NMBE: Natural History Museum of Bern, Bern, Switzerland
NMNHU-P (before 1998 known as IZAN): National Museum of Natural History, National Academy of Sciences of Ukraine, Kyiv, Ukraine
NWM: Natural History Museum Vienna, Vienna, Austria
NU: Nazarbayev University, Astana, Kazakhstan
ONU: I.I. Mechnikov Odesa National University, Odesa, Ukraine
SNM: Slovak National Museum, Bratislava, Slovakia
TNU: V.I. Vernadsky Taurida National University, Kyiv, Ukraine
USNM: Department of Palaeobiology, National Museum of Natural History, Washington, DC, USA
ZMUC: Natural History Museum of Denmark, Copenhagen, Denmark
ZKM: Zaporizhzhia Regional Museum of Local History, Zaporizhzhia, Ukraine
