# Supplementary material for: A new seal from the Late Miocene of the Eastern Paratethys highlights the past regional diversity of true seals (Phocidae)
Source: Swiss J Palaeontol. 2025 Jun 12;144(1):28. doi: 10.1186/s13358-025-00372-7 (PMC12162803; doi:10.1186/s13358-025-00372-7)
Supplement: Supplementary file 2 — Additional file 2 [file 13358_2025_372_MOESM2_ESM.pdf]

Supplementary Data for:

A new seal from the Late Miocene of the Eastern Paratethys highlights the past regional diversity of true seals (Phocidae)

Pavlo Otriazhyi*[1, 2, 3], Theodor Obadă[4,5], Oleksandr Kovalchuk[6, 7, 8], Davit Vasilyan[2,3], and Pavel Gol'din*[1]

Table S1: The list of comparative material of Phocidae bones.

Collection	Number	Species	Bones
ZMUC	1134	*C. cristata*	Skull, mandible, femur, humerus, radius, ulna.
ZMUC	1265	*C. cristata*	Skull, femur, humerus
NMW	1620	*C. cristata*	Skull, mandible
NMW	4027	*C. cristata*	Skull, mandible
NMW	4028	*C. cristata*	Skull, mandible
NMNHU-P	64-455	*C. maeotica*	Femur
MNEIN	144.67	*C. maeotica*	Femur
NMNHU-P	Nordmann N1	*C. maeotica*	Femur
NMNHU-P	64-530	*C. maeotica*	Humerus
SNM	PP 1502	*D. emryi*	Humerus
SNM	PP1503	*D. emryi*	Ulna
SNM	Z14543	*D. emryi*	Coxae
SNM	Z14544	*D. emryi*	Femur
SNM	Z25507	*D. emryi*	Humerus
SNM	Z27870	*D. emryi*	Skull
SNM	Z27871	*D. emryi*	Mandible
SNM	Z27872	*D. emryi*	Mandible
SNM	Z27873	*D. emryi*	Mandible
SNM	Z27874	*D. emryi*	Radius
NMBE	1061	*E. barbatus*	Skull, mandible
NMBE	1939	*E. barbatus*	Skull, mandible
NMW	1950	*E. barbatus*	Scapula, radius, ulna, humerus, femur, tibia, fibula, coxae,

NMW	4026	*E. barbatus*	Skull, mandible
NMW	7556	*E. barbatus*	Skull, mandible, coxae, tibia, fibula, scapula, humerus, ulna, radius, femur
ZMUC	CN 951	*E. barbatus*	Skull
ZMUC	CN 958	*E. barbatus*	Humerus, femur, radius
ZMUC	CN 1204	*H. fasciata*	Skull
ZMUC	CS-303-67	*H. fasciata*	Skull
ZMUC	1485	*Ha. grypus*	Skull, humerus, femur
NMW	28539	*Ha. grypus*	Skull, mandible, coxae, cuboid, tibia, fibula, sacrum, scapula, humerus, ulna, radius, femur
NMW	31588	*Ha. grypus*	Skull, mandible, coxae, cuboid, tibia, fibula, sacrum, scapula, humerus, ulna, radius, femur
NMW	31589	*Ha. grypus*	Skull, mandible, coxae, cuboid, tibia, fibula, sacrum, scapula, humerus, ulna, radius, femur
NMW	66292	*Pu. caspica*	Skull, mandible
NMW	66293	*Pu. caspica*	Skull
NMW	66294	*Pu. caspica*	Skull
NMW	66295	*Pu. caspica*	Mandible (5 specimens)
NMW	66297	*Pu. caspica*	Coxae
NMW	66298	*Pu. caspica*	Scapula (3 spec.), radius (2 spec.), humerus (4 spec.)
NMW	66299	*Pu. caspica*	Femur (3 specimens), tibia, fibula
GNM	2-2013/988	*Pu. caspica*	Skull
ZMUC	154	*Pa. groenlandicus*	Skull, femur
NMW	416	*Pa. groenlandicus*	Skull, mandible
NMBE	638	*Pa. groenlandicus*	Skull, mandible
ZMUC	CN 961	*Pa. groenlandicus*	Skull, humerus, radius, femur
NMBE	1060	*Pa. groenlandicus*	Skull, mandible
NMW	4021	*Pa. groenlandicus*	Skull, mandible
NMW	7753	*Pa. groenlandicus*	Skull, mandible
NMBE	1023561	*Pa. groenlandicus*	Skull, mandible
ZMUC	803	*Pu. hispida*	Skull, humerus, femur

NMNHU-P	64-468	*Pr. tarchankutica*	Skull, Mandible, humerus, radius
NMNHU-P	64-469	*Pr. tarchankutica*	Skull
NMBE	224	*Ph. vitulina*	Skull
NMW	1462	*Ph. vitulina*	Skull, mandible, coxae, tibia, fibula, scapula, humerus, ulna, radius, femur
ZMUC	1599	*Ph. vitulina*	Skull
NMW	28587	*Ph. vitulina*	Skull, mandible, sacrum, humerus, ulna, radius, coxae, tibia, fibula, cuboid, scapula, femur
NMBE	301/91	*Ph. vitulina*	Femur, humerus
PIN	1713-10	*Po. sarmatica*	Humerus, femur
NMNHU-P	40-121	*H. alrkseevi*	Skull
FFM	10246	*M. pontica*	Rostral part of the skull, mandible, vertebrae, ribs, limb bones
TNU	CH00-01	*M. pontica*	Skull
NMW	SK173	*Pr. vindobonensis*	Femur
AICUPM	SF-3	*"Ph". bessarabica*	Proximal fragment of humerus
AICUPM	SF-5	*"Ph". bessarabica*	Metatarsal V

Table S2. List of specimens previously referred to as *Pachyphoca* spp. and similar bones with indication of their localities.

*Old collection numbers

Taxon	Bone elements	Location
Pachyphoca ukrainica	Humerus NMNHU-P 64-701 (holotype), ulna 64-710, 64-383	Khomutove (Homutovo), Ukraine
Pachyphoca chapskii	Femur NMNHU-P OF 1210 (holotype)	Zhovtokamianka, Ukraine
Pachyphoca	Scapula NMNHU-P 64-707, humeri NMNHU-P OF 1208 (64-522*), OF 1209 (64-523*), ulna 64-711, 64-712, OF 1214 (64-518*), OF 1213 (64-519*), coxae OF 1207 (64-525*), tibia OF 1211 (64-520*), fibula OF1212 (64-521*), sacrum OF 1219 (64-527*)	Zhovtokamianka, Ukraine
Pachyphoca	Scapula NMNHU-P 64-477, 64-702, humeri 64-703 64- 713, radius 64-481, 64-482, Ulna 64-705, femora 64-354, 64-471, tibiae and fibulae 64-712, 64-478, 64-473, coxae 64-479, 64-348	Zolota Balka, Ukraine
Pachyphoca	Humeri ZKM P-470, P-492, P-4950, The scapula ZKM P-612, two proximal parts of two different humeri ZKM P-245 (1) and ZKM P-245 (2)	Southern bank of the Kakhovka Reservoir (Zlatopil, Mayachka, Lysa Hora, Skelky), Ukraine
Pachyphoca	Femur 64-166	Gnylozubove (Kalmius), Ukraine
Pachyphoca	Humeri MNEIN FN 144/n/a, Radius MNEIN FN 144/230b, Ulna 144-231, femur 144-132, 144-134, 144-135, 144-136.	Hulbocica, Moldova
Pachyphoca	Scapula MNEIN n/a	Lăpușna, Moldova
Pachyphoca	Femur MNEIN FN 54 294	Pruncul, Moldova
Pachyphoca	Femur AICUPM MS-50	Chisinau, Moldova
Phocinae	Scapulae ONU 3721 (2, 5, 7 8, 10), humeri ONU 3720 (1, 2, 3, 6, 11, 14, 16, 17), femora ONU 3719 (1, 2, 6, 10, 11, 12, 16).	Vyshneve (Kirovo), Ukraine
Phocinae	Femur NMNHU-P 64-140	Kulykivka (Kulikovka), Ukraine

Table S3: measurements of the mandible of *Paratethyphoca libera* MCFFM V150 (in mm)

Measurement	Mandible height at p3	Mandible height at p4	Mandible height at m1	Diastema c - p1	Diastema p1 - p2	Diastema p2 - p3	Diastema p3 - p4	Diastema p4 - m1
Mandible MCFFM V150	17.7	17.8	16	2.3	1.2	2.3	2.5	1.9
	alveola p1	alveola p2	alveola p3	alveola p4				
Mandible MCFFM V150	4.0	5.2	3.2	2.6				

Table S4: Measurements of the manus and pes bones.

Bone	Length in mm
Metacarpal 2	31.5
Metacarpal 3	27.3
Metacarpal 4	27.6
Metacarpal 5	27.8
Forelimb proximal phalanx 2	22.2
Forelimb proximal phalanx 3	23.1
Forelimb proximal phalanx 4	23.6
Forelimb proximal phalanx 5	23.2
Metatarsal 1	87.6
Metatarsal 3	69.8
Metatarsal 4	65.9
Metatarsal 5	83.8
Hindlimb proximal phalanx 1	78.8
Hindlimb proximal phalanx 2	55.6
Hindlimb proximal phalanx 3	51.5
Hindlimb medial phalanx 1	45.8
Hindlimb medial phalanx 2	44.9
Hindlimb medial phalanx 3	47.9
Hindlimb distal phalanx 1	24.5
Hindlimb distal phalanx 3	24.3

Table S5: measurements of phocid humeri based on the manual by Ericson and Stora (1999) with the additional measurement of the deltoid crest. GL – Greatest length. GLCP – (Greatest) length with the condyles in one plane. GLC – Greatest length from the head. DP – Depth of the proximal end. GDC – Greatest transversal diameter of caput. SDB – The smallest diagonal breadth of the diaphysis. SHD – The smallest height of diaphysis. BD – the (Greatest) breadth of the distal end. BT – the (Greatest) breadth of the trochlea, with condyles in one plane. DHC – The greatest diagonal height of condylus lateralis. DCL - The length of the deltoid crest

Measurement	*Paratethyphoca liberia* MCFFM V150	*E. barbatus* ZMUC CN 958	*C. cristata* ZMUC 1134	*C. cristata* ZMUC 1265	*Pa. groenlandicus* ZMUC CN 961	*Ha. grypus* ZMUC 1485	*Ph. vitulina* NMBE 301/91	*Pu. caspica* NMW 66298
GL	113,7	160	143,9	158	125,25	146,7	114,7	91
GLCP	114,8	152	139,5	154.6	126,8	144	109,4	86,1
GLC	120,08	147	137,5	148,8	120,2	132,4	105,7	84,6
DP	61,1	76	66	74,5	63,5	64	56,4	36,7
GDC	32,3	32	31,14	41	28,2	35,5	28,85	19,12
SDB	20	23,85	23,4	28,9	16,5	24,3	17,4	13,15
SHD	21,1	29,66	29	36	26	30,8	24	15,3
BD	44,8	60,2	43	55	43,4	53,5	42,2	30,6
BT	28,8	39,6	37	40	32,4	34,1	29,9	20,6
DHC	35,9	29,1	25	28	24	24,75	20,17	14,4
DCL	57,4	83.9	71.8	76.9	70.2	78.7	58.5	44.2

Table S6: Nucleotide sequences used in phylogenetic analysis.
[a] Fulton & Strobeck 2009, [b] Arnason et al 2006

	FLVCR1	Prepronociceptin	Recombinase activating protein 1	Recombinase activating protein 2
C. cristata	GU167790.1 [a]	GU167825.1 [a]	GU167840.1 [a]	GU167855.1 [a]
Erignathus barbatus	GU167791.1 [a]	GU167826.1 [a]	GU167841.1 [a]	GU167856.1 [a]
Halichoerus grypus	GU167793.1 [a]	GU167828.1 [a]	GU167843.1 [a]	GU167858.1 [a]
Hydrurga leptonyx	GU167800.1 [a]	GU167835.1 [a]	GU167850.1 [a]	GU167865.1 [a]
H. fasciata	GU167798.1 [a]	GU167833.1 [a]	GU167848.1 [a]	GU167863.1 [a]
Lobodon carcinophaga	GU167801.1 [a]	GU167836.1 [a]	GU167851.1 [a]	GU167866.1 [a]
Leptonychotes weddellii	GU167802.1 [a]	GU167837.1 [a]	GU167852.1 [a]	GU167867.1 [a]
Mirounga leonina	-	XM035023169.1 [b]	-	-
Monachus monachus	GU167449.1 [a]	GU167516.1 [a]	-	GU167569.1 [a]
Ommatophoca rossii	GU167799.1 [a]	GU167834.1 [a]	GU167849.1 [a]	GU167864.1 [a]
Pu. caspica	GU167797.1 [a]	GU167832.1 [a]	GU167847.1 [a]	GU167862.1 [a]
Pa. groenlandicus	GU167792.1 [a]	GU167827.1 [a]	GU167842.1 [a]	GU167857.1 [a]
Pu. hispida	GU167794.1 [a]	GU167829.1 [a]	GU167844.1 [a]	GU167859.1 [a]
Ph. vitulina	GU167795.1 [a]	GU167830.1 [a]	GU167845.1 [a]	GU167860.1 [a]
Pu. sibirica	GU167437.1 [a]	GU167504.1 [a]	GU167529.1 [a]	GU167557.1 [a]

Table S7: True landmarks of the humerus.

№	Description of the landmark
1	The lateral most point of the head
2	The cranial most point of the head
3	The medial most point of the head
4	The distal most point of the head
5	Dot of the cross between lines landmark1-landmark3 and Landmark2-landmark4
6	The most proximal point of the lesser tubercule
7	Medial point of the narrowest width of diaphysis
8	Distal most point of the lateral epicondyle
9	Cranial most point of the lateral epicondyle
10	Medial most point of the medial epicondyle
11	Distal most point of the medial epicondyle
12	Proximal-lateral most point of the trochlea (caudal side)
13	Proximal-medial most point of the trochlea (caudal side)
14	Distal-lateral most point of the capitulum
15	Point on the distal edge between the trochlea and the capitulum
16	Distal-medial most point of the trochlea
17	Proximal-medial most point of the trochlea (cranial side)
18	Point on the proximal edge between the trochlea and the capitulum
19	Proximal-lateral most point of the capitulum

Table S8: True landmarks of the femur.

№	Description of the landmark
1	Proximal-medial most point of the medial condyle
2	Proximal-lateral most point of the medial condyle
3	Proximal-medial most point of the lateral condyle
4	Proximal-lateral most point of the lateral condyle
5	Distal-medial most point of the medial condyle
6	Distal-lateral most point of the medial condyle
7	Distal-medial most point of the lateral condyle
8	Distal-lateral most point of the lateral condyle
9	Proximal-medial most point of the patellar facet
10	Proximal-lateral most point of the patellar facet
11	The most distal point of the patellar facet
12	The most distal point of greater trochanter
13	The most caudal point of intertrochanteric line
14	The most proximal point of greater trochanter
15	Point where greater trochanter transits to the femoral neck
16	The cranial most point of the head
17	The lateral most point of the head
18	The caudal most point of the head
19	The distal most point of the head

Table S9: Materials used for Generalized Procrustes Analysis

* Phocini

Species	Number	Humerus	Femur
C. cristata	ZMUC 1134	+	+
C. cristata	ZMUC 1265	+	+
E. barbatus	ZMUC CN 958	+	+
E. barbatus	NMW 1950	+	+
E. barbatus	NMW 7556		+
H. grypus *	NMW 31588	+	+
H. grypus *	NMW 31589	+	+
H. grypus *	ZMUC 1485	+	+
Pu. caspica *	NMW 66298 1	+	
Pu. caspica *	NMW 66298 2	+	
Pu. caspica *	NMW 66298 3	+	
Pu. caspica *	NMW 66299 1		+
Pu. caspica *	NMW 66299 2		+
Pu. caspica *	NMW 66299 3		+
Pa. groenlandicus	ZMUC CN 961	+	+
Pa. groenlandicus	ZMUC 154		+
Pu. hispida *	ZMUC 803	+	+
Pu. sibirica *	NMW 38399	+	+
Pu. sibirica *	NMW 38400	+	+
Pu. sibirica *	NMW 40895		+
Ph. vitulina *	NMBE 301/91	+	+
Ph. vitulina *	NMW 1462		+
Ph. vitulina *	NMW 28587		+
Cr. maeotica	NMNHU-P 64-530	+	
Cr. maeotica	NMNHU-P Nordmann (4)	+	
Cr. maeotica	MNEIN 144.219	+	
Cr. maeotica	MNEIN 144.7	+	
Cr. maeotica	NMNHU-P 64-455		+
Cr. maeotica	NMNHU-P Nordmann N1		+
Cr. maeotica	MNEIN 144-133		+
Cr. maeotica	MNEIN 144.67		+
Devinophoca sp.	SNM Z25507	+	
M. pontica	NMNHU-P 64-257	+	
M. pontica	NMNHU-P OF 1008	+	
M. pontica	NMNHU-P OF 1010	+	
M. pontica	NMNHU-P 64-256		+
M. pontica	FFM 10246 left		+
M. pontica	FFM 10246 right		+
M. pontica	NMNHU-P OF 1005		+
M. pontica	NMNHU-P OF 1007		+
Phocinae gen. sp. from Hrytsiv	NMNHU-P n/a		+
Phocinae gen. sp. from Kulykivka	NMNHU-P 64-140		+
"Pp." ukrainica	ZKM 4950(16)	+	

"Pp." ukrainica	NMNHU-P 64-701	+	
"Pp." ukrainica	ZKM p-470	+	
"Pp." ukrainica	ZKM p-492 (1-4)	+	
"Pp." ukrainica	ZKM P-492 19 (3)	+	
"Pachyphoca" sp	AICUPM MS-50		+
"Pachyphoca" sp	MNEIN 144-132		+
"Pp." chapskii	NMNHU-P OF 1209	+	
"Pp." chapskii	ZKM P-492 4	+	
"Pp." chapskii	NMNHU-P OF 1210		+
"Pp." ukrainica	MNEIN 55-294		+
"Pp." ukrainica	NMNHU-P 64-166		+
"Pp." ukrainica	NMNHU-P 64-158		+
"Pp." ukrainica	MNEIN 144-134		+
Paratethyphoca	MCFFM V-150	+	

Table S10: approximate date of the node received Bayesian analysis with 95% highest probability density range, and posterior probability.

**Node number**	Age in Ma	95% highest probability density	Posterior Probabilities
1	38.13	30.8 – 46.80	1
2	28.09	28.09 – 28.09	0.12
3	23.25	20.39 – 27.65	0.55
4	7.24	7.24 – 7.24	0.18
5	33.15	28.10 – 39.48	0.46
6	27.09	23.01 – 32.97	0.93
7	24.71	22.99 – 27.99	0.36
8	15.51	12.41 – 19.33	1
9	11.42	8.39 – 15.11	0.92
10	9.28	7.56 – 11.54	0.82
11	13.73	11.61 – 16.38	0.54
12	11.32	8.80 – 14.35	0.87
13	10.42	8.22 – 12.95	0.32
14	9.19	7.59 – 11.20	0.47
15	6.79	4.56 – 9.36	1
16	5.36	3.76 – 7.29	0.99
17	3.46	1.21 – 5.87	1
18	21.57	18.51 – 25.59	0.86
19	18.38	15.45 – 21.94	0.33
20	17.76	14.89 – 21.06	0.44
21	14.50	13.79 – 15.97	0.92
22	20.04	18.09 – 22.55	0.22
23	18.26	15.89 – 21.05	0.34
24	16.25	14.58 – 18.37	0.83
25	14.49	14 .49 – 14.49	0.25
26	12.72	10.46 – 15.22	0.97
27	10.73	8.00 – 13.35	0.44
28	9.09	6.35 – 11.79	0.88
29	7.37	5.16 – 9.87	0.91
30	4.13	2.75 – 5.79	0.99
31	5.17	3.50 – 6.98	0.80
32	4.20	3.9 – 5.56	1
33	1.42	0.43 – 2.66	1
34	0.68	0.10 – 1.43	0.96

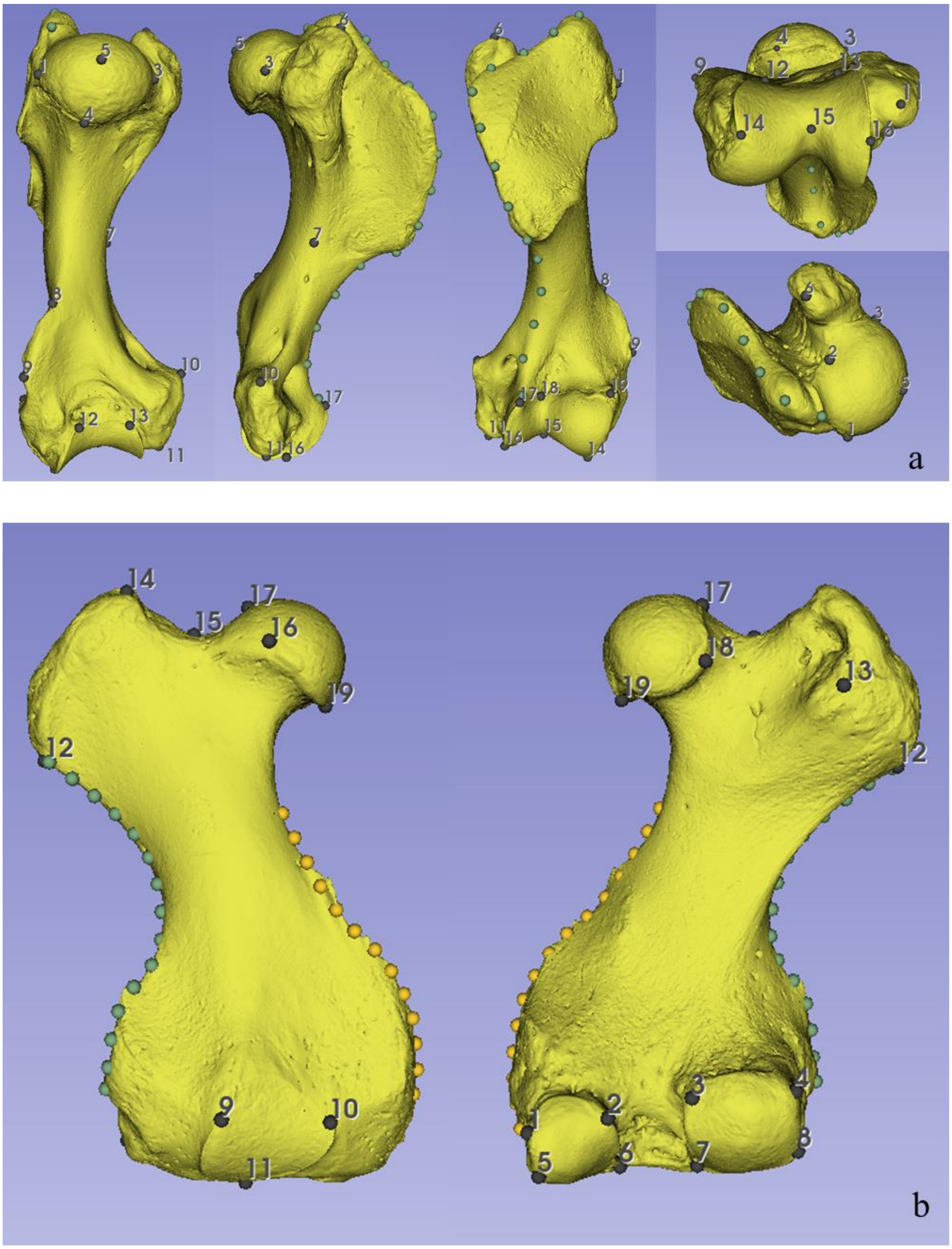

Figure S1: Landmarks of the humerus (a) and femur (b) used in Generalized Procrustes Analysis

(a) Grey landmarks – 19 true landmarks marking the main anatomical features of the bone; Green landmarks – 15 semilandmarks equally spread on the curve from the greater tubercle through the deltoid crest and to the trochlea.

(b) Grey landmarks – 19 true landmarks marking the main anatomical features of the femur, green landmarks – 15 semilandmarks equally spread on the lateral edge from the distal edge of the greater trochanter to the distal end of the lateral epicondyle. Yellow landmarks – 15 semilandmarks equally spread on the medial edge of the femur from the narrowest point of the diaphysis till the distal end of the medial epicondyle.

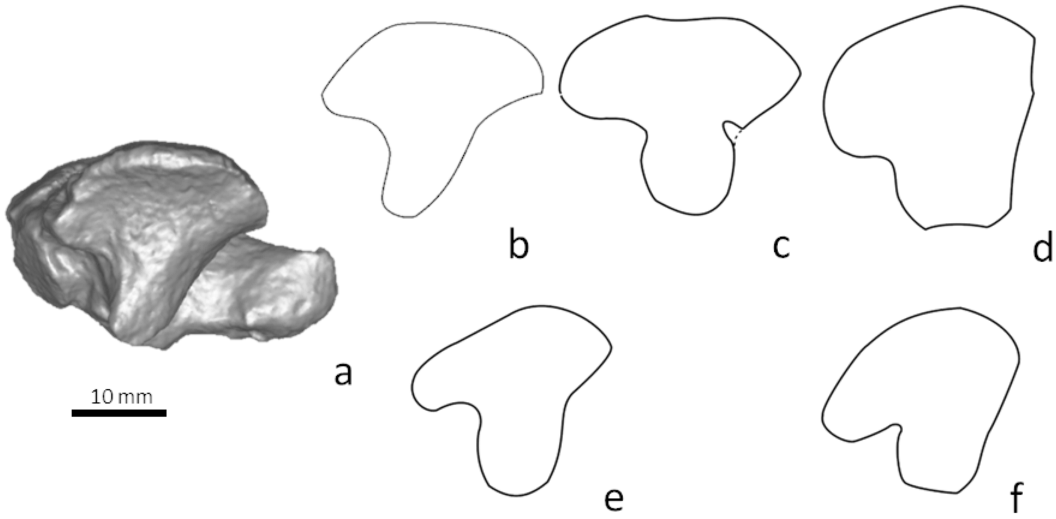

Figure S2: Comparison of cuboids.

(a) – 3D model of *Paratethyphoca libera* MCFFM V-150 cuboid; (b-e) – edges of surface for 3rd cuneiform; (b) *M. pontica* CH05; (c) – *Cystophora cristata* USNM-550317; (d) – *Erignathus barbatus* UWBM-34220; (e) – *Halichoerus grypus* USNM-504481. Species c-e, redrawn from virtual.imnh.iri.isu.edu.

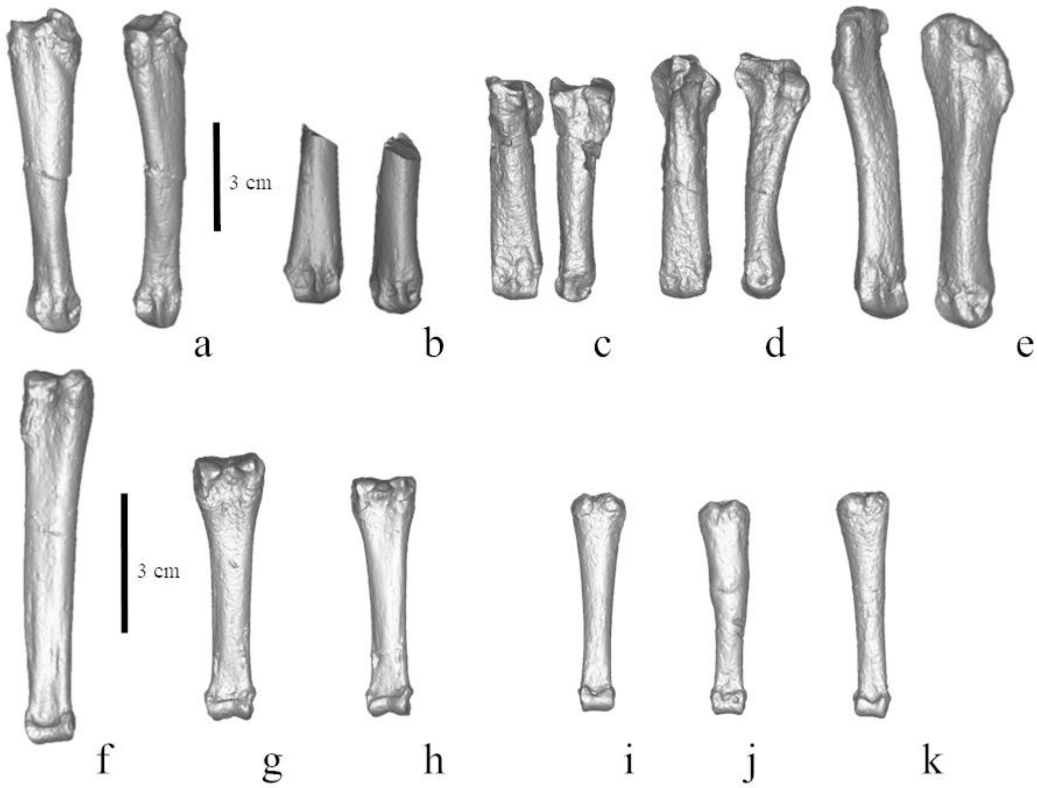

Figure S3: Flipper bones of *Paratethyphoca libera* MCFFM V-150

(a) – Metatarsal I; (b) – Metatarsal II; (c) – Metatarsal III; (d) – Metatarsal IV; (e) – Metatarsal V; (f) – proximal phalanx I; (g) – proximal phalanx II; (h) – proximal phalanx III; (i) – medial phalanx I; (j) – medial phalanx II; (k) – medial phalanx III.

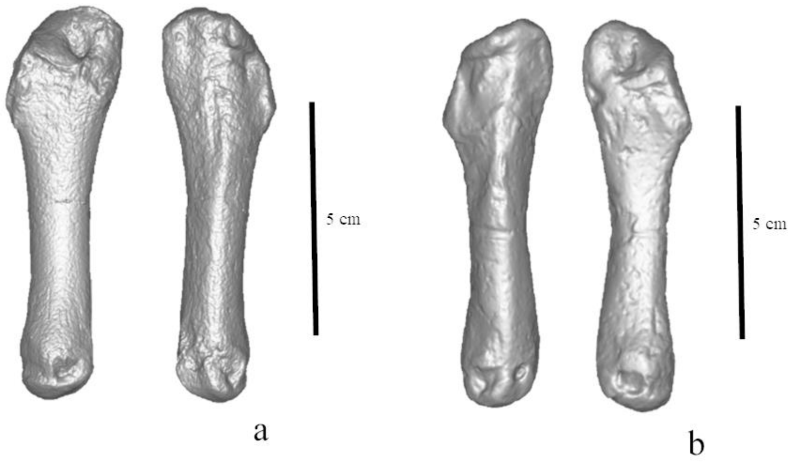

Figure S4. Metatarsal 5 of *Paratethyphoca libera* MCFFM V-150 (a) and "*Phoca*" *bessarabica* SF-7 (b)

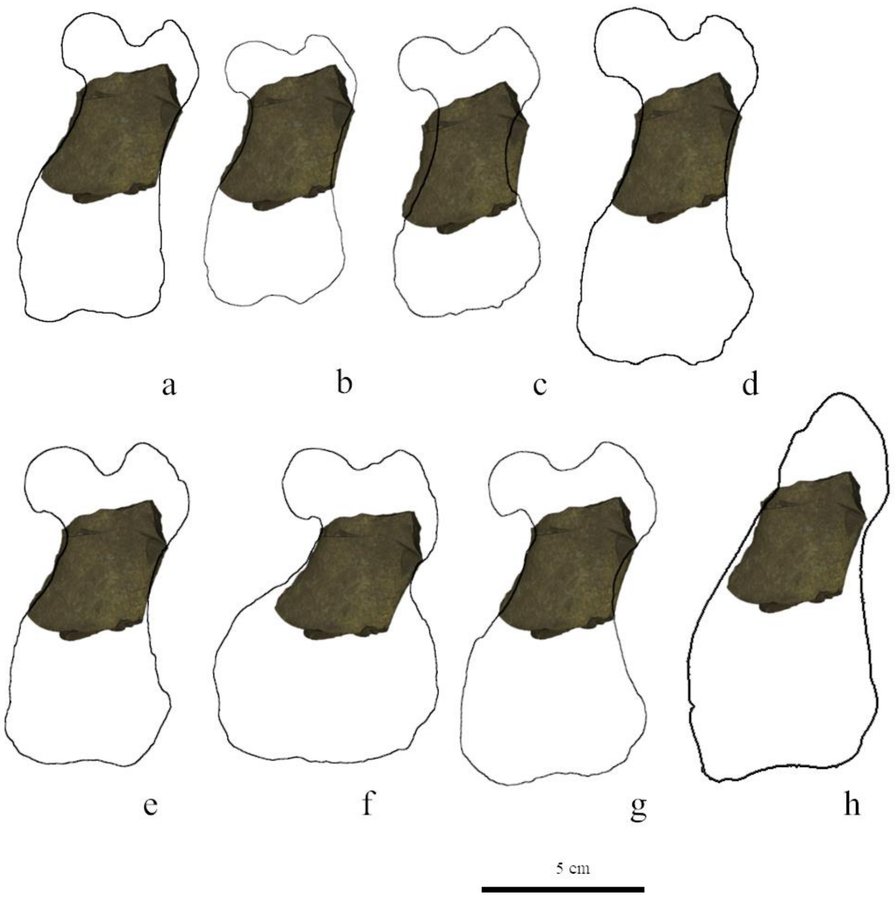

Figure S5: Comparison of the femur *Paratethyphoca libera* MCFFM V-150 with outlines of other Paratethyan seals.

(a) – Outline of Phocidae MNEIN FN 54 294; (b) – outline of Phocidae NMNHU-P 64-354; (c) – outline of *Cryptophoca maeotica* NMNHU-P n/a; (d) – outline of *Cryptophoca maeotica* MNEIN FN 144-133; (e) – Outline of Phocidae AICUPM MS-50; (f) – outline of "*Po*". *sarmatica* NMNHU-P 1713/10; (g) – outline of *Cr. maeotica* NMNHU-P 1713 23; (h) – Outline of "*Pp." chapskii* NMNHU-P 1210.

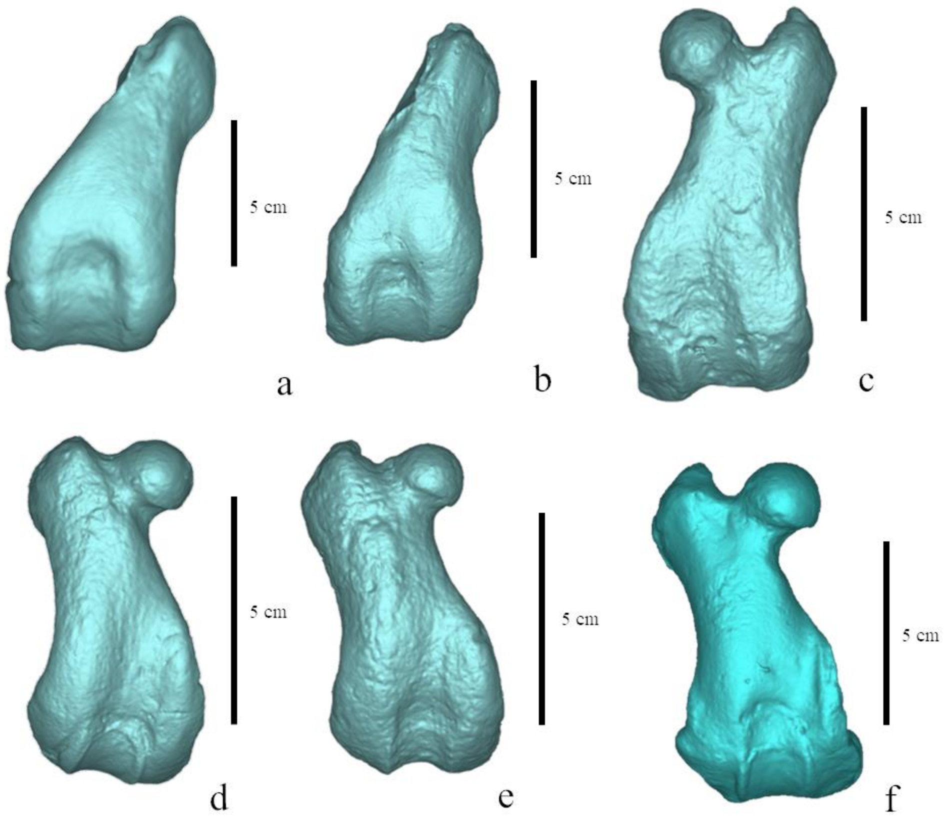

Figure S6: Comparison of pachyosclerotic femora of Phocidae from the Eastern Paratethys

(a) – *“Pp.” chapskii* NMNHU-P OF 1210; (b) – *”Pachyphoca”* sp. FN 144-136; (c) – *”Pachyphoca”* sp. FN 55-294; (d) – *“Pp.” ukrainica* NMNHU-P 64-166; (e) – *“Pp.” ukrainica* NMNHU-P 64-354; (f) – *”Pachyphoca”* sp. MS 50.

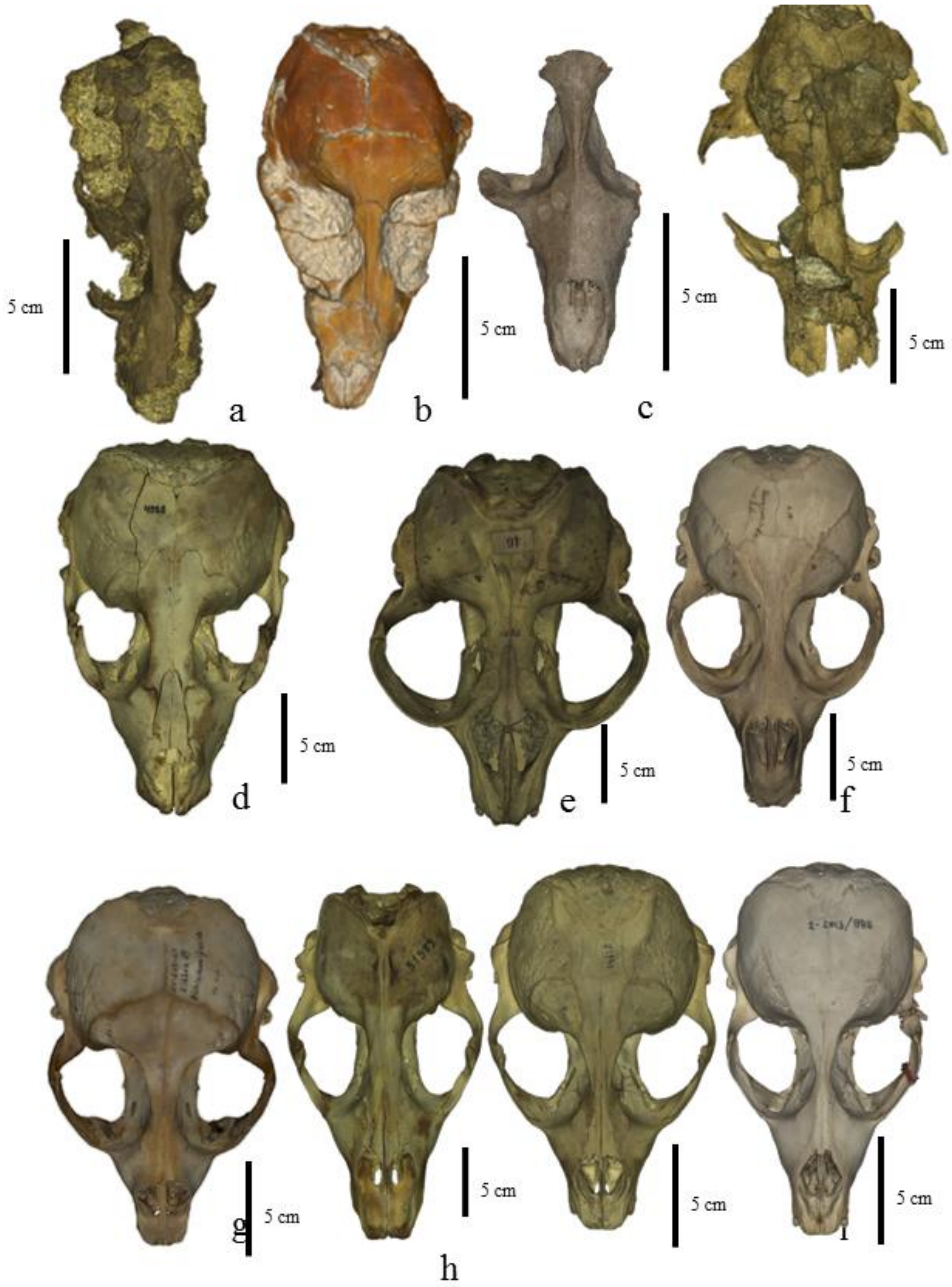

Figure S7: Comparative anatomy of seal skulls, dorsal view

(a) – *Paratethyphoca libera* MCFFM V-150; (b) – *Pr. tarchankutica* NMNHU-P 64-468; (c) – *M. pontica* TNU CH00-01; (d) – *D. emryi* SNM Z 25507; (e) – *E. barbatus* NWM 4026; (f) – *C. cristata* NWM 1620; (g) – *Pa. groenlandicus* NMBE 638; (h) – *H. fasciata* ZMUC CS-303-67; (i) – *Ha. grypus* NWM 31588; (j) – *Ph. vitulina* NWM 1462; (k) – *Pu. caspica* GNM 2-2013/98

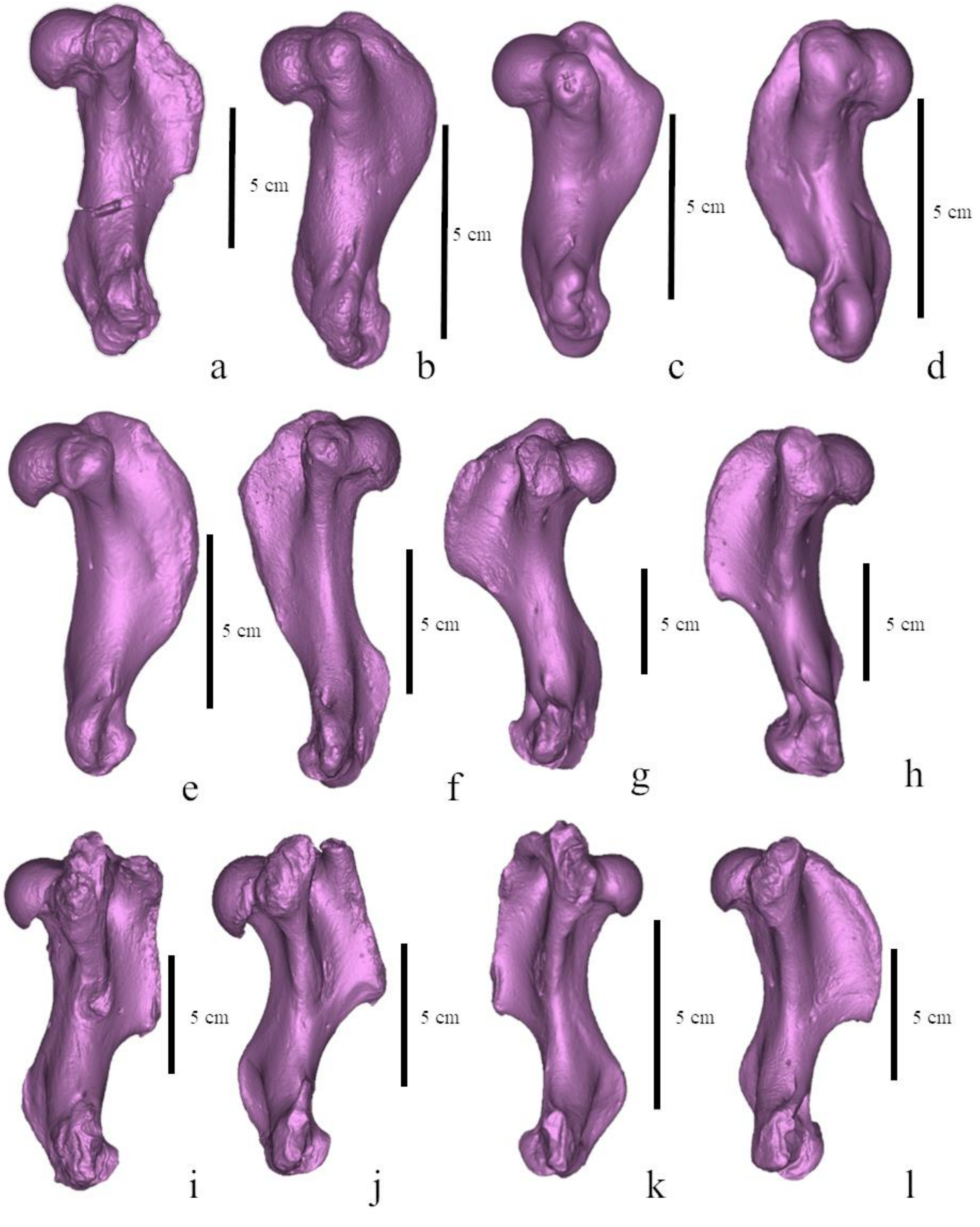

Figure S8: Comparison of humeri in medial view.

(a) – *Paratethyphoca libera* MCFFM V-150; (b) – *“Pp.” ukrainica* NMNHU-P 64-701; (c) – *“Pp.” ukrainica* ZKM P-492 (1-4) a; (d) – *M. pontica* NMNHU-P 64-257; (e) – *C. maeotica* NMNHU-P 64-530; (f) – *D. emryi* SNM Z 25507; (g) – *E. barbatus* CN 958 (NHMD); (h) – *C. cristata* 1134 (NHMD); (i) – *Ha. grypus* 1485 (NHMD); (j) – *Ph. vitulina* NMBE 301-91; (k) – *Pu. caspica* NMW 66298; (l) – *Pa. groenlandicus* CN 961 (NHMD). Bars are 5 cm.

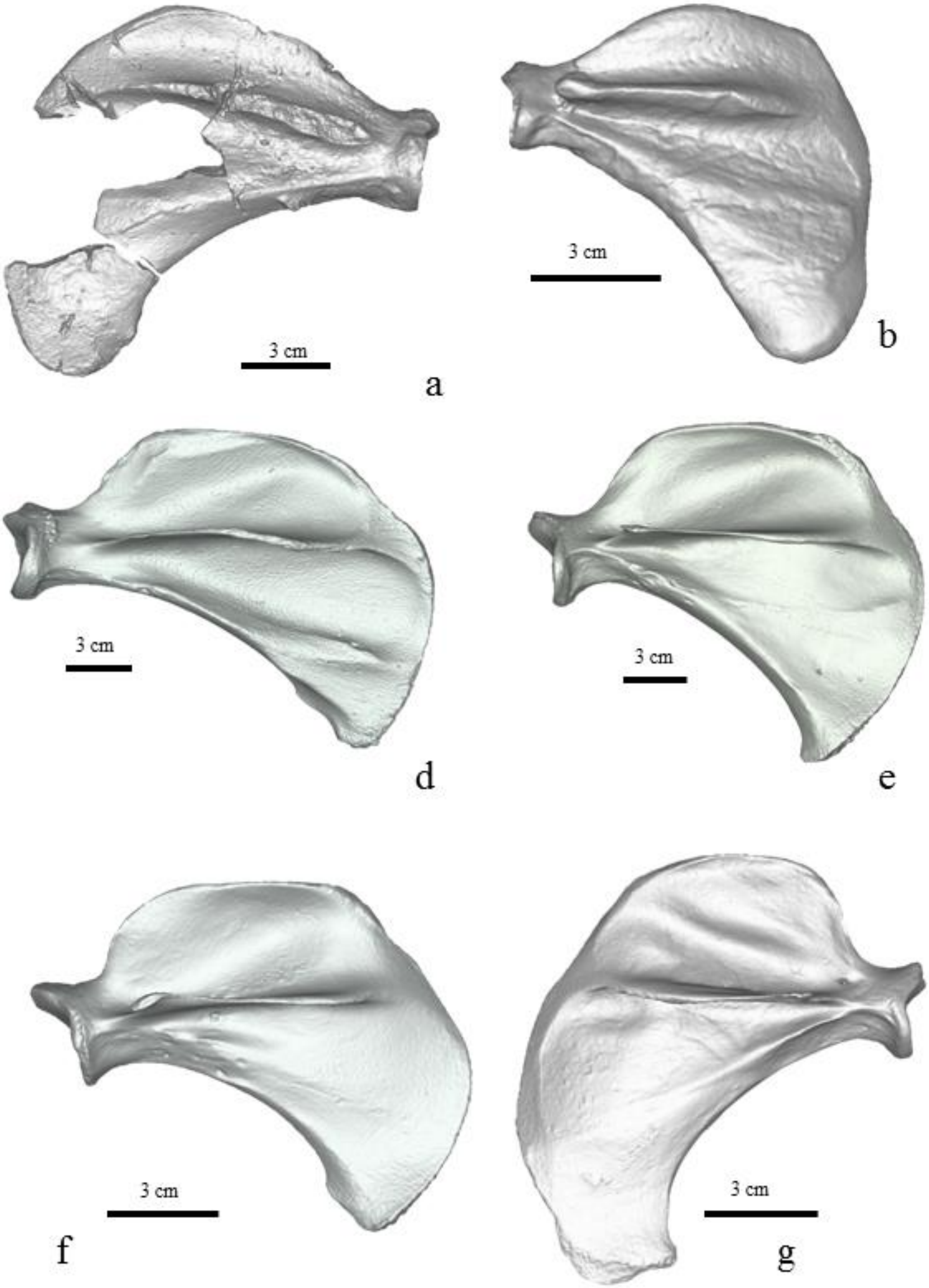

Figure S9: Comparison of seal scapulae, posterior view

(a) – *Paratethyphoca libera* MCFFM V-150; (b) – *M. pontica* FFM 10246; (c) – *E. barbatus* NWM 1950; (d) – *Ha. grypus* NWM 31589; (e) – *Ph. vitulina* NWM 1462; (f) – *Pu. caspica* NWM 66298.

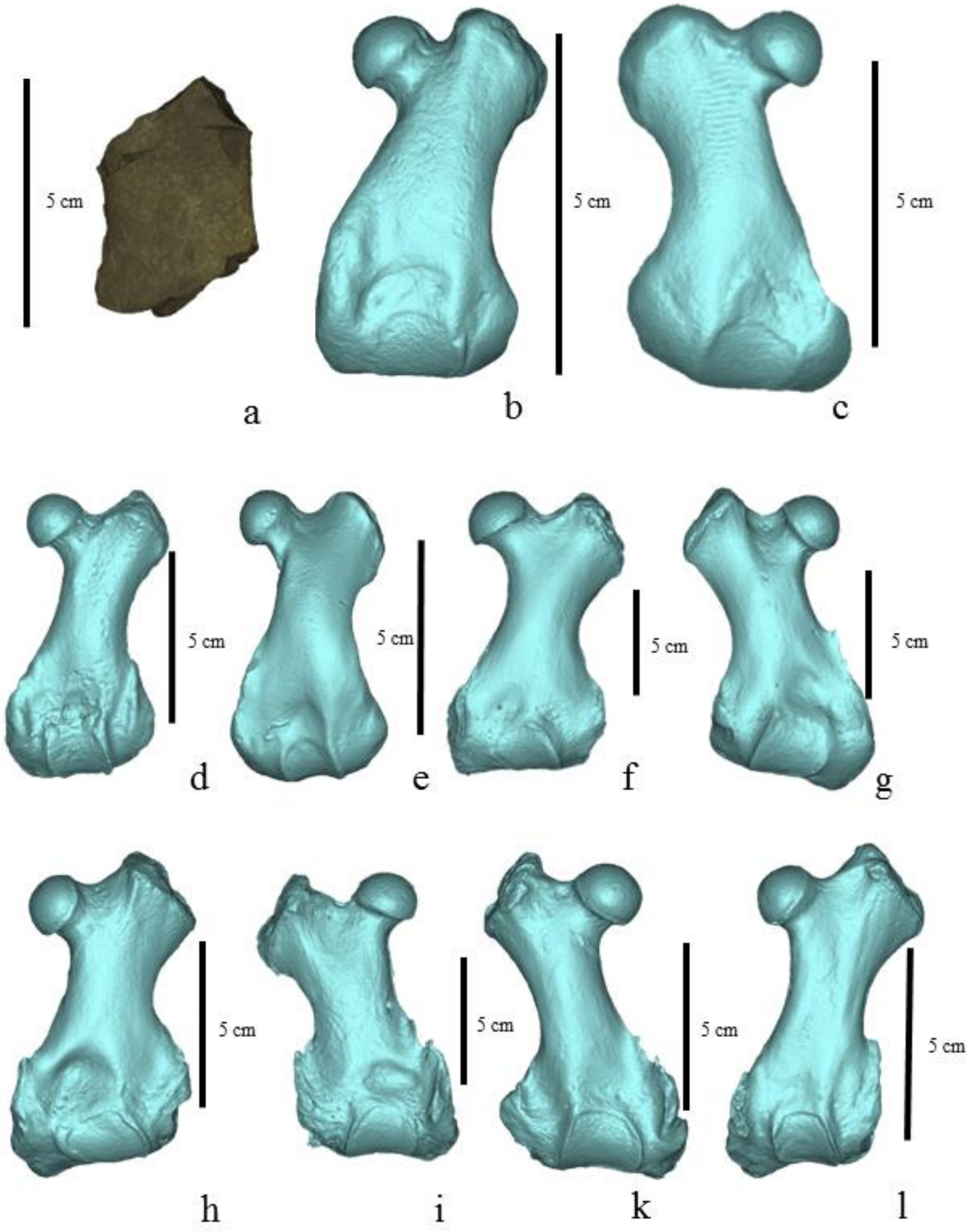

Figure S10: Comparison of femora of true seals (Phocidae) in cranial view.

(a) – *Paratethyphoca libera* MCFFM V-150; (b) – *M. pontica* NMNHU-P 64-256; (c) – *M. pontica* NMNHU-P 64-314; (d) – *C. maeotica* NMNHU-P n/a; (e) *C. maeotica* NMNHU-P 64-455; (f) – *E. barbatus* CN 958 (NHMD); (g) – *C. cristata* 1134 (NHMD); (h) – *Pa. groenlandicus* 154 (NHMD); (i) – *Ha. grypus* 1485 (NHMD); (k) – *Ph. vitulina* NMBE 301 91; (l) – *Pu. caspica* NMW 66299

Bars are 5 cm

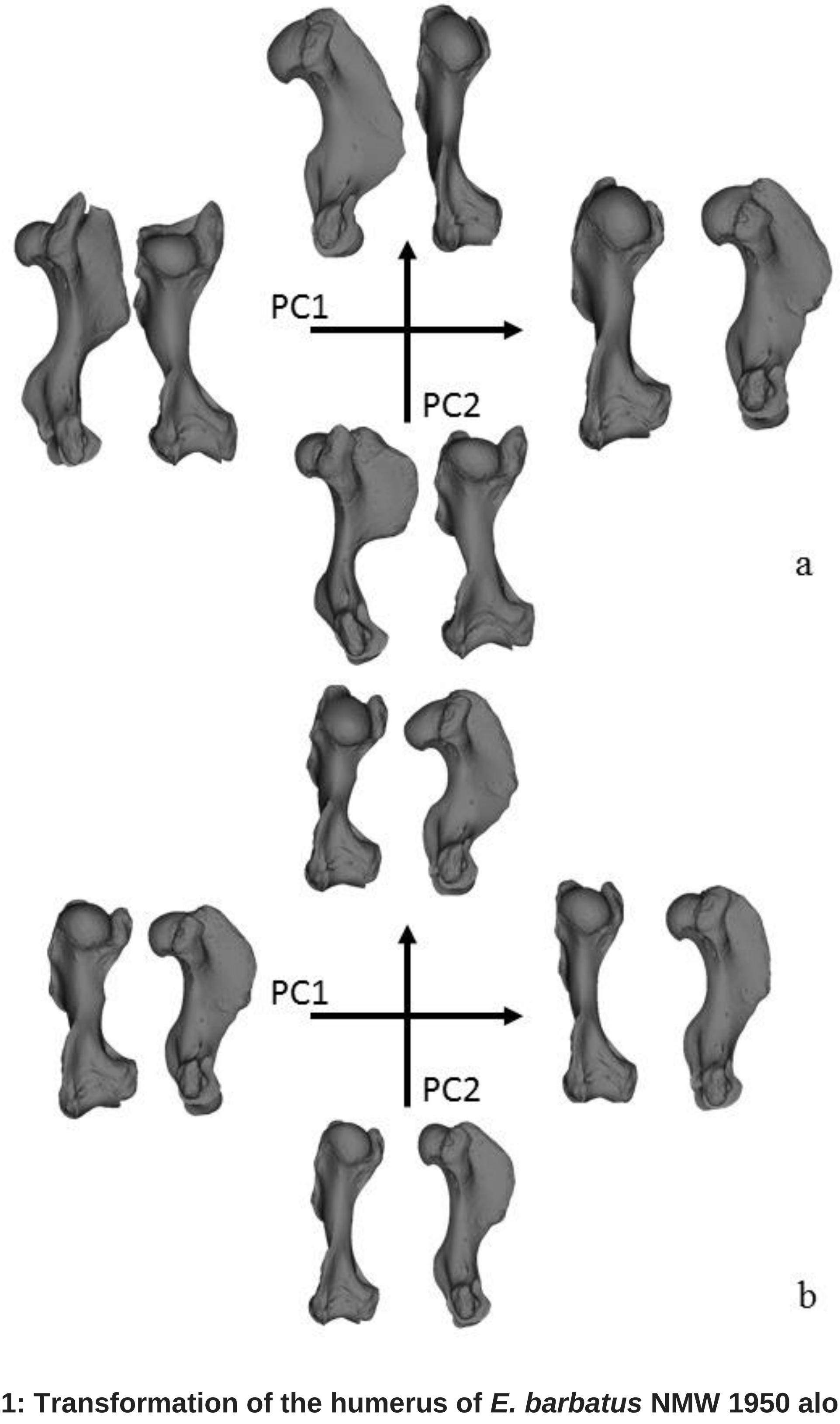

Figure S11: Transformation of the humerus of *E. barbatus* NMW 1950 along PC1 and PC2 using interactive 3D visualization in SlicerMorph module for 3DSlicer.

(a) – With Phocini included in the dataset; (b) – Phocini excluded from the dataset.

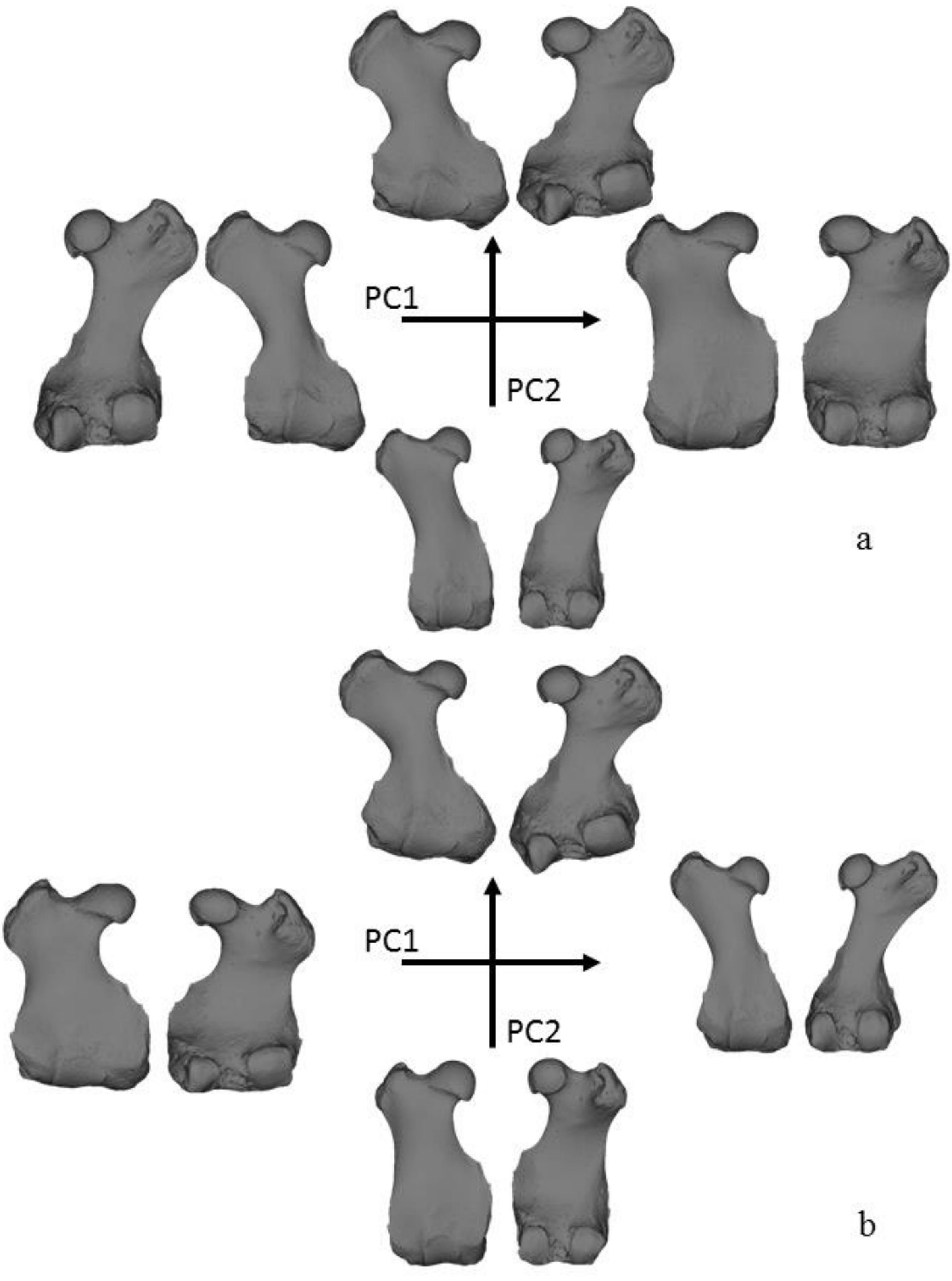

Figure S12: Transformation of the femur of *E. barbatus* NMW 1950 along PC1 and PC2 using interactive 3D visualization in SlicerMorph module for 3DSlicer

(a) – With Phocini included in the dataset; (b) Phocini excluded from the dataset.

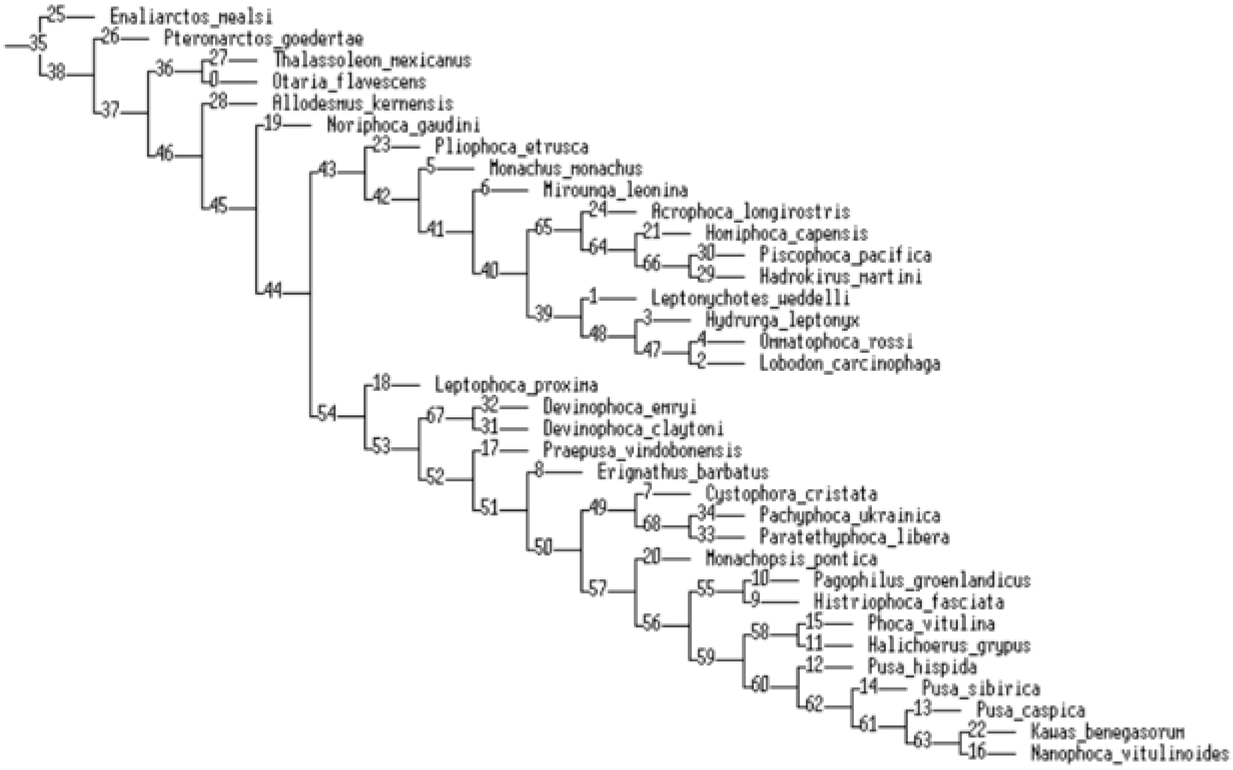

Figure S13: Parsimony tree of Phocidae, *Paratethyphoca libera* MCFFM V-150 combined with ilium NMNHU-P OF 1207

Implied weighting (k=3), Consistency index 0.352, retention index 0.705.

Supplementary files: titles and legends/captions

Title: The list of morphological characters (DOCX)

Legend/Caption: The list and description of morphological characters used for phylogenetic analysis

Title: Parsimony.tnt

Legend/Caption: The file includes morphological matrix and constrained tree of modern taxa for phylogenetic analysis in TNT 1.6

Title: Supplement Parsimony with illium.tnt

Legend/Caption: The file includes morphological matrix and constrained tree of modern taxa for phylogenetic analysis in TNT 1.6. The morphological matrix of *Paratethyphoca libera* also includes ilium NMNHU-P OF 1207.

Title: Seals_morph.nex

Legend/Caption: The file includes matrix of morphological characters for phylogenetic analysis in BEAST 2.5. Morphological matrix based on Dewaele et all (2017), Dewaele et all (2018), and Koretsky (2001).

Title: FLVCR1.nex

Legend/Caption: The file includes molecular matrix of FLVCR1 for phylogenetic analysis in BEAST 2.5 (see Table S4 for details and references).

Title: PNOC.nex

Legend/Caption: The file includes molecular matrix of prepronociceptin PNOC for phylogenetic analysis in BEAST 2.5 (see Table S4 for details and references).

Title: RAG1.nex

Legend/Caption: The file includes molecular matrix of Matrix of recombinase activating protein 1 RAG1 for phylogenetic analysis (see in BEAST 2.5 Table S4 for details and references).

Title: RAG2.nex

Legend/Caption: The file includes molecular matrix of recombinase activating protein 2RAG2 for phylogenetic analysis in BEAST 2.5 (see Table S4 for details and references).

Title: Paratethyphoca final 1.xml

Legend/Caption: The setting for the Bayesian analysis 1 made using BEAUti for further using in BEAST 2.5

Title: Paratethyphoca final 2.xml

Legend/Caption: The setting for the Bayesian analysis 2 made using BEAUti for further using in BEAST 2.5

Title: Paratethyphoca final 1.tree

Legend/Caption: the summarised tree obtained from .trees file which was obtained from the first iteration of the phylogenetical analysis in BEAST 2.5

Title: Paratethyphoca final 2.tree

Legend/Caption: the summarised tree obtained from .trees file which was obtained from the second iteration of the phylogenetical analysis in BEAST 2.5

Title: pcScores Humerus 1

Legend/Caption: Results of generalized Procrustes analysis results for the humerus

Title: pcScores Humerus 2

Legend/Caption: Results of generalized Procrustes analysis results for the humerus (no Phocini)

Title: pcScores Femur 1

Legend/Caption: Results of generalized Procrustes analysis results for the femur

Title: pcScores Femur 2

Legend/Caption: Results of generalized Procrustes analysis results for the femur (no Phocini)

Dewaele, L., Lambert, O., & Louwye, S. (2017). On *Prophoca* and *Leptophoca* (Pinnipedia, Phocidae) from the Miocene of the North Atlantic realm: redescription, phylogenetic affinities and paleobiogeographic implications. *PeerJ*, *5*, e3024. https://doi.org/10.7717/peerj.3024

Dewaele, L., Lambert, O., & Louwye, S. (2018). A critical revision of the fossil record, stratigraphy and diversity of the Neogene seal genus *Monotherium* (Carnivora, Phocidae). *Royal Society Open Science*, *5*, 171669. http://dx.doi.org/10.1098/rsos.17166

Koretsky, I. A. (2001). Morphology and systematics of Miocene Phocinae (Mammalia: Carnivora) from Paratethys and the North Atlantic region. *Geologica Hungarica Series Palaeontologica*, *54*, 1–109.
