# Supplementary material for: A new seal from the Late Miocene of the Eastern Paratethys highlights the past regional diversity of true seals (Phocidae)
Source: Swiss J Palaeontol. 2025 Jun 12;144(1):28. doi: 10.1186/s13358-025-00372-7 (PMC12162803; doi:10.1186/s13358-025-00372-7)
Supplement: Supplementary file 16 — Additional file 16 [file 13358_2025_372_MOESM16_ESM.docx]

1 'Premaxilla-nasal suture: (0) extensive; (1) weakly reduced; (2) strongly reduced. (3) absent'

2 'Premaxilla-maxilla suture: 0) entirely lateral to nasal cavity; 1) anterior portion partially included in nasal cavity; (2) entirely within nasal cavity.’,

3 'Nasals, shape of posterior edge: (0) pointed; (1) rounded or square; (2) frontals insert between nasals.’,

4 'Facial angle: (0) snout more anteriorly than dorsally (angle 45° or more); (1) snout opening more dorsally than anteriorly (angle 45° or less).’,

5 'Lateral border of the opening of the nasal cavity in lateral view: (0) rectilinear or weakly concave; (1) strongly concave.’,

6 'Position of posterior end of nasals: (0) anterior to maxilla-frontal suture; (1) posterior to maxilla-frontal suture but greatly anterior to the level of the jugal-squamosal suture; (2) almost reaches the level of the jugal-squamosal suture.’,

7 'Maxilla swelling: (0) not present; (1) present.’,

8 'Alveolar process of maxilla: (0) facing ventrally; (1) facing anteroventrally posterior to P1’,

9 'Maxillary process of jugal (at level of the anteroventral border of the orbit) in lateral view: (0) thin and low and increasing progressively posteriorly; (1) thick and high and increasing abruptly posteriorly.’,

10 'Position of anterior opening of infraorbital foramen in ventral view: (0) anterior to M1; (1) level or posterior to M1.’,

11 'Jugal, anterior end in dorsal view: (0) lateral to infraorbital foramen; (1) above or medial to the lateral margin of the infraorbital foramen.’,

12 'Jugal, direction of arch of anterior portion: (0) downwards; (1) flat, no distinct arch; (2) upwards.’,

13 'Squamosal-jugal articulation: (0) splintlike; (1) mortised. (adapted from Berta & Wyss, 1994)’,

14 'Ventral edge of the zygomatic arch, in anterior view: (0) higher than alveolar plane; (1) level with the alveolar plane (or very close to).’,

15 'Supraorbital process of frontal: (0) absent or weakly developed; (1) strongly developed.’,

16 'Orbital vacuities: (0) absent; (1) present. (Berta & Wyss, 1994)’,

17 'Interorbital, least width: 0) in posteriormost portion of interorbital septum; 1) in anterior half of the interorbital septum.’,

18 'Interorbital least width: braincase width ratio: (0) high (i.e., much more than 20% of width of skull); (1) moderate (i.e., approximately 20%); (2) low (i.e., much less than 20%); (3) very low (i.e., 5% or less).’,

19 'Major axes of glenoid fossae: (0) sub-parallel; (1) slightly convergent posteriorly.’,

20 'Orientation of medial margins of tympanic bullae: (0) diverging posteriorly; (1) parasagittal.’,

21 'Lateral end of tympanic bulla: (0) medial to level of mid-width of glenoid fossa; (1) lateral to level of mid-width of glenoid fossa.’,

22 'Inflation of tympanic bulla: (0) weak; (1) moderate; (2) strong.’,

23 'Carotid canal, posterior opening: (0) visible in ventral view (i.e., at least partially facing ventrally); (1) not visible in ventral view (i.e., opening having very little ventral aspect).’,

24 'Posterior opening of the carotid canal and posterior lacerate foramen: (0) clearly separated; (1) coalescent.’,

25 'Mastoid: 0) not visible in dorsal view; 1) visible (Berta and Wyss 1994).’,

26 'Heavily pachyosteosclerotic mastoid: (0) absent; (1) present.’,

27 'Relation of paroccipital process to mastoid: (0) connected by a low and discontinuous ridge; (1) connected by a high and continuous ridge; (2) well separated.’,

28 'Pterygoid process: (0) rounded with convex lateral margin; (1) flat with concave lateral margin.’,

29 'Alisphenoid canal: (0) present; (1) absent.’,

30 'Direction of occipital condyles in occipital view: (0) ventral; (1) diverging dorsally.’,

31 'Tooth rows: (0) parallel; (1) diverging posteriorly.’,

32 'Upper incisors: (0) three; (1) two; (2) one (ordered).’,

33 'Lower incisors: (0) three; (1) two; (2) one (ordered).’,

34 'Lateral incisor, relative size: (0) incisiform; (1) intermediate shape; (2) caniniform.’,

35 'Upper incisor, roots: (0) strongly transversely compressed; (1) moderately transversely compressed.’,

36 'P2-4, p2-4, roots: (0) at least one triple-rooted; (1) double-rooted; (2) single-rooted (according to Berta and Wyss, 1994).’,

37 'Postcanine teeth, crowns: (0) prominently multi-cusped; (1) single-cusped or weakly multi-cusped.’,

38 'Postcanine teeth, lingual cingulum: (0) well developed; (1) not or poorly developed’,

39 'M1 and m1, roots: 0) double-rooted or triple-rooted; 1) single-rooted.’,

40 'M2: (0) present; (1) absent.’,

41 'p4, size comparison m1: (0) about equal in size; (1) p4 larger than m1.’,

42 'Atlas, transverse foramen: (0) visible in posterior view; (1) at least partially visible in dorsal view.’,

43 'Atlas, direction of transverse process in lateral view: (0) oblique; (1) sub-vertical.’,

44 'Scapula: (0) two ridges on lateral side do not join near glenoid; (1) two ridges on lateral side join near glenoid.’,

45 'Humerus, lesser tubercle and head: (0) head higher or at same level as lesser tubercle; (1) tubercle higher.’,

46 'Humerus, greater tubercle height: (0) below level of head; (1) at level of head or slightly above; (2) above level of head.’,

47 'Humerus, supinator: (0) strongly developed; (1) poorly developed.’,

48 'Humerus, deltopectoral crest; (0) smooth distal termination; (1) sharp distal termination.’,

49 'Humerus, deltoid crest proximal bifurcation: (0) present, either distinct or slight; (1) absent, crest has smooth edge.’,

50 ''Humerus, length of deltoid crest: (0) shorter than or subequal to one-half length of the bone, confined to the proximal half of the bone; (1) longer than one-half length of the bone’,

51 'Humerus, intertubercular groove: (0) narrow and deep; (1) intermediate state; (2) broad and shallow’,

52 'Humerus, transverse bar in bicipital groove: (0) absent; (1) present.’,

53 'Humerus, entepicondylar foramen: (0) absent; (1) present’,

54 'Humerus, diameter of trochlea: (0) same as diameter of distal head; (1) considerably larger than distal head. (Berta & Wyss, 1994) ‘,

55 'Radius, location radial tuberosity: 0) medial side; 1) posteromedial side.’,

56 'Radius, pronator teres process: 0) present, proximal; 1) present, distal (adopted from Berta and Wyss).’,

57 'Ulna, distal end of styloid process: 0) distally pointed; 1) flattened.’,

58 'Metacarpal I, length: (0) slightly longer than mcII; (1) much longer.’,

59 'Metapodials, head: (0) keeled with trochleated phalangeal articulations; (1) smooth, with phalanges flat, articulations hingelike.’,

60 'Sacrum, number of fused vertebrae: (0) three; (1) four.’,

61 'Innominate, anterodorsal iliac spine: (0) dorsal to anteroventral iliac spine; (1) posterodorsal to the anteroventral iliac spine’,

62 'Innominate, posteroventral iliac spine (=iliac tuberosity): (0) small or absent; (1) large and strongly protruding.’,

63 'Innominate, iliopectineal eminence: (0) strongly developed; (1) moderately well developed; (2) small or absent.’,

64 'Innominate, ilium: (0) shallow gluteal fossa; (1) deep gluteal fossa.’,

65 'Innominate, ilium: (0) weakly everted wing; (1) moderately everted; (2) strongly everted wing.’,

66 'Innominate, ilium: (0) long, compared to postacetabular region; (1) short.’,

67 'Innominate, ischial spine: (0) unenlarged; (1) enlarged.’,

68 'Femur, lesser trochanter: (0) present; (1) absent (Berta and Wyss 1994:54).’,

69 'Femur, neck, relative size: (0) thick; (1) narrow.’,

70 'Femur, collo-diaphyseal angle: (0) high, head oriented more medially than proximally; (1) low, head oriented more proximally than medially.’,

71 'Femur, distal condyles: (0) roughly similar in size or slight size difference; (1) large size difference.’,

72 'Femur, epiphyses: (0) distal epiphysis wider than proximal; (1) widths of proximal and distal epiphyses about equal; (2) proximal epiphysis wider than distal one.’,

73 'Femur, diaphysis: (0) minimum width less than or about equal to two-thirds width of proximal epiphysis; (1) minimum width more than two-thirds width of proximal epiphysis.’,

74 'Femur, head and greater trochanter: (0) head reaches higher than greater trochanter; (1) both reach same level; (2) greater trochanter reaches higher than head.’,

75 'Femur, trochanteric fossa: (0) little reduced; (1) strongly reduced or absent.’,

76 'Femur, intertrochanteric crest: (0) strongly reduced; (1) relatively pronounced.’,

77 'Femur, suprapatellar fossa: (0) absent; (1) present.’,

78 'Femur, orientation of fossa for m. peroneus longus: (0) lateral; (1) anterolateral’,

79 'Tibia and fibula: (0) proximal epiphyses not fused; (1) proximal epiphyses fused.’,

80 'Tibia, development post-tibial fossa: (0) weak; (1) strong’,

81 'Astragalus, calcaneal process: (0) absent; (1) poorly developed; (2) well developed (ordered).’,

82 'Sustentacular facet of the astragalus: (0) oval-shaped and narrowed at contact with cuboid facet; (1) long (at least twice longer than wide), slender and strongly bent medially; (2) short and tongue-like with no narrowing at contact with cuboid facet.’,

83 'Calcaneum, articular surface for fibula: (0) absent or very reduced; (1) well developed.’,

84 'Metatarsal I, articular surface for metatarsal II: (0) oriented laterally; (1) oriented dorsolaterally; (2) inconspicuous.’,

85 'Metatarsal III, length: (0) less than 50% shorter than metatarsal I; (1) approximately 50% shorter (or more) than metatarsal I.'

86 'Shape of head of malleus: (0) broad and circular; (1) slender and elliptic.',

87 'Premolars: (0) parallel to toothrow axis; (1) obliquely oriented in toothrow.',

88 'Diastema between P4 and M1: (0) large; (1) reduced.',

89 Palatal groove: (0) present; (1) absent.
90 Palatal process of maxilla: (0) flattened; (1) convex.

91 supraorbital process: (0) absent, (1) present

92 Chin prominence: (0) developed, (1) reduced
